# Developing organoboranes as phase transfer catalysts for nucleophilic fluorination using CsF†

**DOI:** 10.1039/d2sc00303a

**Published:** 2022-02-09

**Authors:** Sven Kirschner, Matthew Peters, Kang Yuan, Marina Uzelac, Michael J. Ingleson

**Affiliations:** EaStCHEM School of Chemistry, The University of Edinburgh David Brewster Road Edinburgh EH9 3FJ UK michael.ingleson@edinburgh.ac.uk

## Abstract

Despite the general high fluorophilicity of boron, organoboranes such as BEt_3_ and 3,5-(CF_3_)_2_C_6_H_3_–BPin are shown herein for the first time, to our knowledge, to be effective (solid to solution) phase-transfer catalysts for the fluorination of certain organohalides with CsF. Significant (up to 30% e.e.) chiral induction during nucleophilic fluorination to form β-fluoroamines using oxazaborolidine (pre)catalysts and CsF also can be achieved. Screening different boranes revealed a correlation between calculated fluoride affinity of the borane and nucleophilic fluorination reactivity, with sufficient fluoride affinity required for boranes to react with CsF and form Cs[fluoroborate] salts, but too high a fluoride affinity leading to fluoroborates that are poor at transferring fluoride to an electrophile. Fluoride affinity is only one component controlling reactivity in this context; effective fluorination also is dependent on the ligation of Cs^+^ which effects both the phase transfer of CsF and the magnitude of the [Cs⋯F-BR_3_] interaction and thus the B–F bond strength. Effective ligation of Cs^+^ (*e.g.* by [2.2.2]-cryptand) facilitates phase transfer of CsF by the borane but also weakens the Cs⋯F–B interaction which in turn strengthens the B–F bond – thus disfavouring fluoride transfer to an electrophile. Combined, these findings indicate that optimal borane mediated fluorination occurs using robust (to the fluorination conditions) boranes with fluoride affinity of *ca.* 105 kJ mol^−1^ (relative to Me_3_Si^+^) under conditions where a signficant Cs⋯F–B interaction persists.

## Abstract 2

Simple boranes with the optimal fluoride ion affinity are effective as catalysts for phase transfer nucleophilic fluorination with CsF.

## Graphical abstract

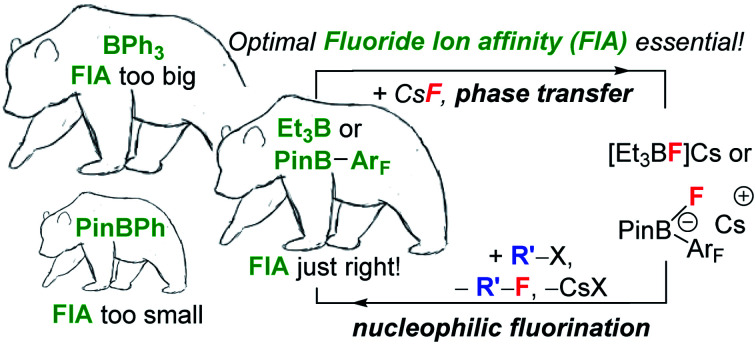

## Introduction

**Fig. 1:**
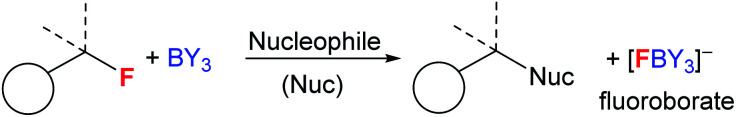
Established reactivity of boranes as fluorophilic Lewis acids.^4^

Boranes are ubiquitous in chemistry and most commonly utilised for their Lewis acidic character. The established dogma is that boranes (BY_3_) are strong Lewis acids towards fluoride, with the derived fluoroborates, [F–BY_3_]^−^, being highly stable towards loss of fluoride.^1^ Many of the most widely used boranes, such as BX_3_ (X = halide) and B(C_6_F_5_)_3_, are indeed strong Lewis acids towards fluoride and form robust fluoroborates,^2^ with [BF_4_]^−^ being an archetypal weakly coordinating anion.^1^ Furthermore, boranes such as B(C_6_F_5_)_3_, and even HBR_2_,^3^ are increasingly applied in defluorinative functionalisation of fluorocarbons, with fluoride abstraction by the borane to form a fluoroborate anion a key step (Fig. 1).^4^ However, by controlling the relative Lewis acidity of the carbon and boron electrophiles it is possible to effect fluoride transfer from fluoroborates to carbon electrophiles. One classic example is [BF_4_]^−^ reacting as a stoichiometric fluoride source in the Balz–Schiemann reaction, but this requires a highly reactive aryl^+^ electrophile.^5^ To expand the utility of fluoroborates in nucleophilic fluorinations it is highly desirable to: (i) use sub-stoichiometric fluoroborate and stoichiometric MF, *i.e.* use boranes as MF solid to solution phase transfer catalysts; (ii) fluorinate carbon electrophiles less reactive than *e.g.* aryl^+^.

To expand the electrophile scope amenable to fluorination with fluoroborates requires an understanding of the factors controlling the fluoride ion affinity (FIA) of boranes, thereby enabling its rational modulation. Analysis of calculated FIA values reveals that borane fluorophilicity can be attenuated by: (i) the presence of significant B=Y multiple bond character; (ii) reducing the partial positive charge localised at boron using less electron withdrawing substituents, and (iii) increasing the pyramidalisation energy at boron.^6^ The first two points combined explains the trend in the fluoride affinity of the simple (herein simple refers to facile to make or commercially available and inexpensive) boranes: BF_3_ (most Lewis acidic, FIA = 258 kJ mol^−1^) ≫ trialkylboranes (FIA of BMe_3_ = 132 kJ mol^−1^) > B(OH)_3_ (FIA = 106 kJ mol^−1^, FIA values relative to Me_3_Si^+^).^6^ Despite the facile ability to tune fluoride affinity at boron there are no reports, to the best of our knowledge, that utilise low FIA boranes as catalysts for MF phase transfer fluorination. Due to the importance of fluorinated molecules in pharmaceuticals and agrochemicals^7^ and the attractive nature of using metal fluoride (MF) salts and simple boranes to effect nucleophilic fluorination, we sought to: (i) demonstrate that low fluoride affinity boranes can be used as MF phase transfer catalysts and (ii) develop the structure activity relationships key to enabling this reactivity.

**Fig. 2:**
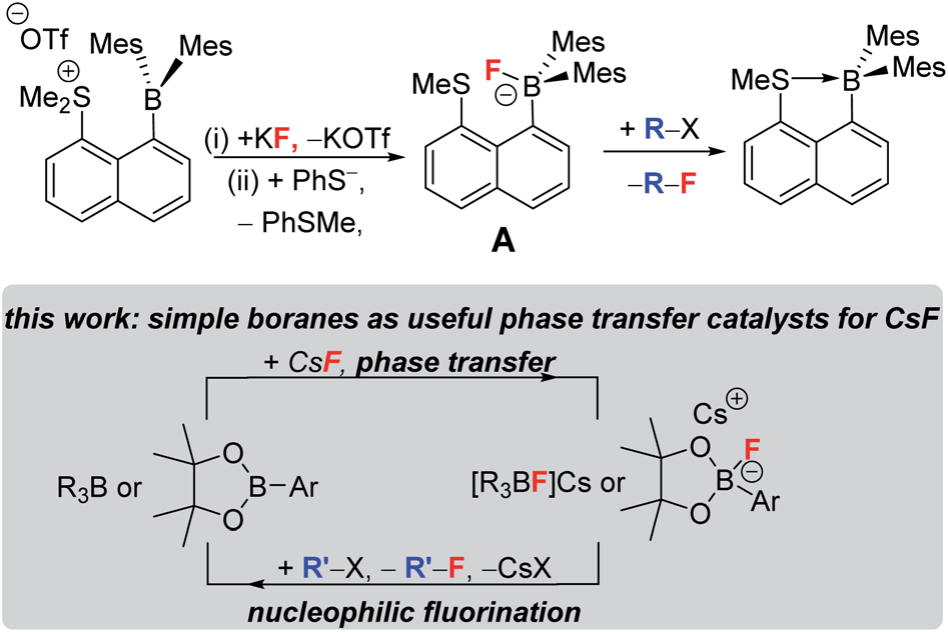
Top stoichiometric fluorination using a dative bond donor functionalised borane. Bottom, this work using simple boranes as CsF phase transfer catalysts.

Phase transfer catalysts are well established in the field of nucleophilic fluorination as the very low solubility of MF in non-protic solvents (required for sufficient fluoride nucleophilicity) necessitates their use.^8,9*a*^ Established phase transfer agents include metal chelators (*e.g.* cryptands), organic cations (*e.g.* [R_4_N]^+^),^9^ Lewis acids that weakly bind fluoride (*e.g.* in hypercoordinated silicates) and compounds that function as multiple hydrogen bond donors to fluoride, *e.g.* bis-ureas.^8,9^ Highly notable recent work using the latter class also achieved excellent (>85% e.e.) enantioselectivity during phase transfer nucleophilic fluorination of certain alkylhalides (*e.g.* β-haloamines) with MF.^8^ Boranes with low FIA (relative to BF_3_) have been largely overlooked in this area. Even the stoichiometric use of fluoroborates derived from lower fluoride affinity boranes in nucleophilic fluorination is rare, with the very limited exceptions including: the use of PinBF in the ring opening fluorination of epoxides;^10^ the use of fluoroborate A (Fig. 2, top) to fluorinate a range of organic electrophiles;^11^ the use of Mes_2_B(aryl) compounds to bind, and on addition of [CN]^−^, to release fluoride.^12^ Note, when using compound A (or when adding an exogenous nucleophile to [Mes_2_B(aryl)F]^−^), the formation of a B←SR_2_ dative bond (or a B–CN bond) contributes to making fluoride transfer from boron to carbon thermodynamically favourable. This factor will be absent using Lewis base free conditions/boranes in MF phase transfer/nucleophilic fluorination cycles (Fig. 2, bottom).

Herein we demonstrate that simple (and Lewis base free) boranes are useful CsF phase transfer fluorination catalysts. Furthermore, we have elucidated important factors controlling the effectiveness of low FIA boranes as CsF phase transfer fluorination catalysts. Demonstrating that simple boranes can act as CsF phase transfer fluorination catalysts opens the door to using the plethora of readily synthesised enantioenriched boranes^13^ in enantioselective nucleophilic fluorination.

## Results and discussion

Initially we sought to determine if the fluoroborates derived from low fluoride affinity triorganoboranes will transfer fluoride to weaker (than aryl^+^) carbon electrophiles, as suggested by previous computational studies.^14^ For these initial studies [NMe_4_]^+^ salts were used to minimise any complications associated with strong interactions between anion and cation. In contrast, significant R_3_B–F⋯M (M = group 1 metal cation) interactions are expected, particularly in weakly coordinating solvents, which could modify fluorination reactivity using M[R_3_BF] salts. [NMe_4_][FBPh_3_] was synthesised by combination of BPh_3_ and [NMe_4_][F] and combined with [Ph_3_C][B(C_6_F_5_)_4_]. This resulted in fluoride transfer from boron to carbon as indicated by ^11^B (change in *δ*_11B_ from 3.4 for [FBPh_3_]^−^ to 60.5 for BPh_3_) and ^19^F NMR spectroscopy (Ph_3_CF observed as the major product, *δ*_19F_ = 126.6). The use of the ethyl congener, [NMe_4_][FBEt_3_], resulted in an analogous outcome (BEt_3_ and Ph_3_CF formation). Therefore in contrast to [BF_4_]^−^ (which does not transfer fluoride to Ph_3_C^+^), these [R_3_BF]^−^ anions do transfer fluoride to Ph_3_C^+^ (note Ph_3_C^+^ is a significantly weaker carbon electrophile than the aryl^+^ species fluorinated in the Balz–Schiemann reaction by [BF_4_]^−^).

To guide subsequent studies and identify other boranes with potential as phase transfer fluorination catalysts we calculated fluoride ion affinity values using a closely related method to that reported by Greb *et al.*^6^ These values are a useful initial indicator of utility in this context, as sufficient fluoride affinity is required for the borane to react with MF and form the fluoroborate salt, but if the FIA is too great then subsequent transfer of fluoride from the fluoroborate to an electrophile will be disfavoured. Therefore the borane with the lowest fluoride affinity value that enables phase transfer of a MF salt was our initial target as this should have the maximum fluorination scope as it will form the most nucleophilic fluoroborate (*i.e.* the fluoroborate with the weakest B–F bond).

**Fig. 3:**
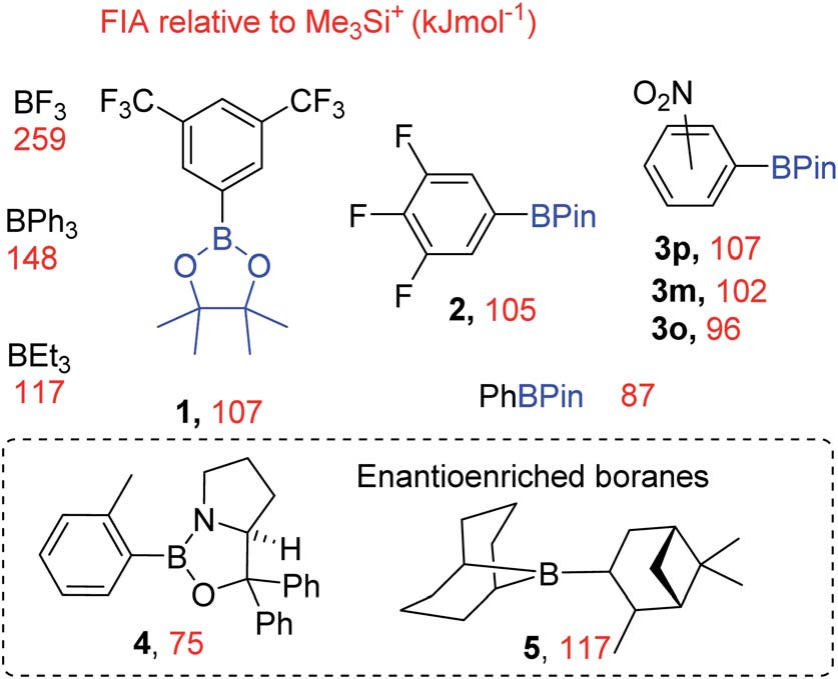
Boranes employed as phase transfer catalysts in this study and their respective calculated (at the DSD-BLYP-D3(BJ)/def2TZVP level with SMD CH_2_Cl_2_) fluoride ion affinity (FIA, red). 3o, 3m and 3p = the *ortho*, *meta* and *para* isomers.

These calculations (Fig. 3) enabled us to identify commercially available boranes (including two enantioenriched examples) spanning a range of fluoride affinity values for study, with the value for BF_3_ at this level provided for comparison. The calculations were consistent with the expected outcomes *e.g.* electron withdrawing groups (in 1-3) increase fluoride affinity (relative to PhBPin). While increased multiple bond character, *e.g.* B=NR_2_ double bond character being greater than B=OR double bond character, leads to CBS (Corey–Bakshi–Shibata) oxazaborolidine catalyst 4 being a weaker Lewis acid towards fluoride than PhBPin. Several boranes with very similar calculated fluoride affinity values also were identified to probe the effect different functional groups (*e.g.* NO_2_*vs.* CF_3_ in 1 and 3x), *ortho vs. meta vs. para* substitution (in 3x) and substituent size (*e.g.* BEt_3_*vs.*5) have on borane reactivity towards MF and the subsequent reactivity of the fluoroborate. This is important as in contrast to [R_4_N]^+^, solvation of M^+^ and F^−^ needs to be considered along with the effect of strong interactions between M^+^ and the fluoride of the fluoroborate persisting in solution.

### Nucleophilic fluorination with CsF

Fluorination of 6 to form β-fluoroamine, 7, using MF (M = K or Cs) catalysed by boranes was explored as a test reaction to determine if there is any correlation between borane fluoride affinity and phase transfer/nucleophilic fluorination reactivity (Table 1). Attempts to perform the fluorination of 6 with KF (with 1 or BEt_3_ as catalyst) led to no fluorination in CHCl_3_, thus all further fluorination studies were performed using CsF. The disparity between KF and CsF is attributed to the greater lattice energy of KF relative to CsF effecting the energetics of the reaction with borane (*vide infra*). It is noteworthy that the use of ground CsF led to substantial rate enhancements *versus* reactions using as received CsF. This is consistent with an increase in surface area facilitating the phase transfer reaction between solid CsF and the dissolved borane. Ground and dried CsF is used throughout this study. With both BEt_3_ and ArBPin based boranes haloalkane solvents gave better outcomes than other solvents, *e.g.* MeCN, thus only results in DCM or chloroform are discussed in depth. Anhydrous conditions are essential, as the presence of water (either using non-purified chloroform, or a 99.5 : 0.5 chloroform/H_2_O volume ratio) led to a significant retardation in the rate of fluorination of 6 using 1. The use of protic additives was not explored with BR_3_ species due to their propensity to undergo protodeboronation with ROH. Finally, a control in the absence of borane led to no fluorination of 6 with CsF in chloroform.

**Table 1:** Outcome of fluorination depending on the borane catalyst^a^

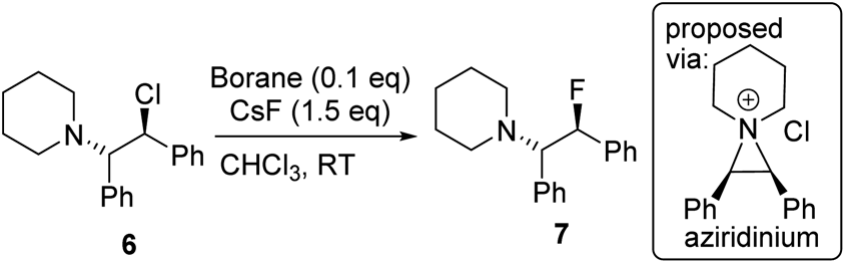			
Borane	FIA (kJ mol^−1^)	Time (h)	Conversion (%)
B(C_6_F_5_)_3_	254^a^	24	<5^b^
BPh_3_	148	24	40^b^
BEt_3_	117	24	88^c^
1	107	8	99^b^
2	105	18	73^c^
3p	107	24	17^b^
3m	102	8	54^b^
3o	96	8	93^b^
PhBPin	87	24	26^b^

a Reaction conditions: 6 (0.2 mmol), borane (10 mol%), CsF (0.3 mmol), CHCl_3_ (anhyd., 5 mL), room temperature, 1000 rpm. a: value from ref. 6; b: conversion (by ^1^H NMR integration of 7*vs.*6); c: isolated yield.

From this borane scoping, phase transfer fluorination of 6 using CsF was most effective with 10 mol% BEt_3_ and 1. This demonstrates that borane phase transfer catalysts can be used to access important fluorinated molecules.^8^ As expected the identity of the borane is all important, with weaker Lewis acids *e.g.* PhBPin, and stronger Lewis acids (*e.g.* BPh_3_) both giving poorer outcomes. The former is consistent with a minimum fluoride affinity being required to form the Cs[fluoroborate] salt, while the latter indicates that if the fluoride affinity is too high then this disfavours transfer of fluoride from boron in the fluoroborate to the electrophile (fluoroborate formation is observed with the higher FIA boranes). However, there are additional factors beyond fluoride affinity controlling fluorination using boranes, as 3p was a relatively poor catalyst despite having an identical calculated fluoride affinity to 1. Furthermore, the *meta* and *ortho* derivatives, 3m and 3o were more active than 3p, despite similar FIA values. Finally, a Hammett analysis (see Fig. S5†) using a range of 4-Y–C_6_H_4_–BPin (Y = MeO, H, F, Cl, Br, CF_3_, NO_2_) boranes led to effectively no correlation, indicating other effects are impacting the fluorination outcome (*vide infra*).

**Scheme 1:**
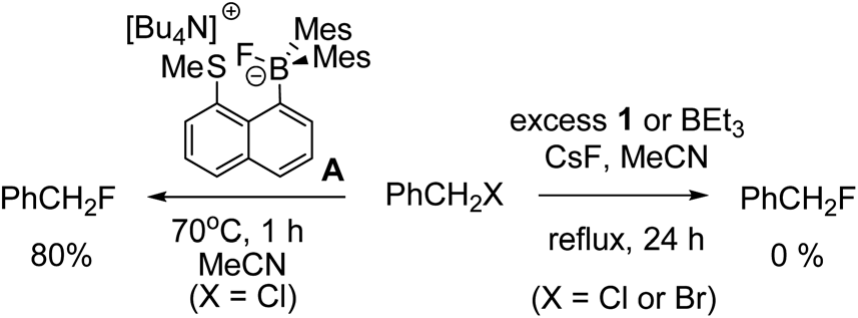
Disparate outcomes in the fluorination of benzyl halides with boranes.

A brief electrophile scoping study was performed using BEt_3_ and 1 as catalysts and this revealed the fluoroborates derived from these boranes to be poorer sources of fluoride relative to the Lewis base incorporated borate A. For example, no fluorination of octyl bromide or benzyl halides was observed even after prolonged periods refluxing with excess borane/CsF (Scheme 1). In contrast, using two eq. of A generated high yields of PhCH_2_F,^11^ demonstrating the positive effect the B←SR_2_ dative bond has in enhancing fluoride transfer ability.

**Fig. 4:**
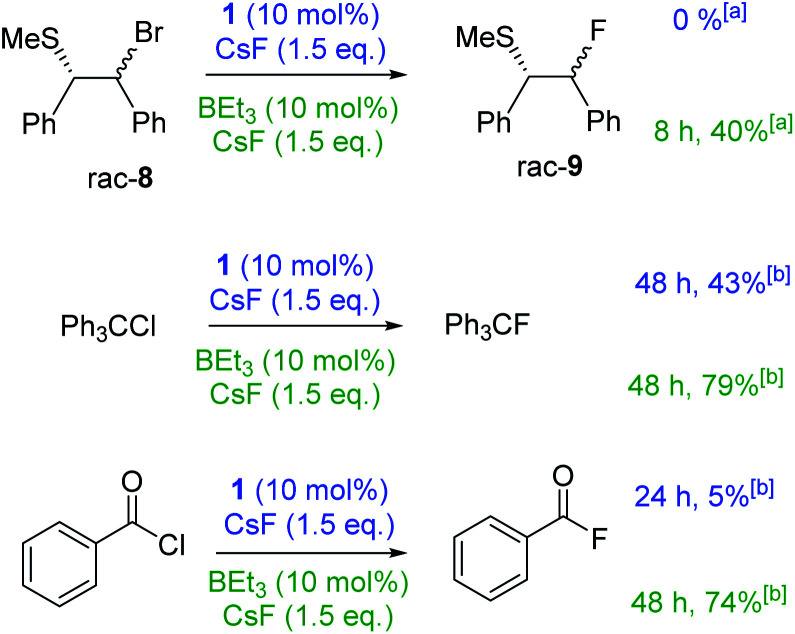
Scope of the borane catalysed fluorination reaction. Conditions: substrate (0.2 mmol), CsF (0.3 mmol), borane (10 mol%), CHCl_3_ (anhyd., 5 mL), room temperature, 1000 rpm. (a) Reaction performed in DCM/*n*-hexane = 6 : 1; (b) conversion gauged by ^19^F qNMR *vs.* 1,2-difluorobenzene as internal standard.

Stronger electrophiles (than PhCH_2_Br) did undergo fluorination with CsF using 1 or BEt_3_ as catalysts. Reaction of β-bromo sulphide 8 with CsF with either BEt_3_ or 1 as catalyst in CHCl_3_ led to significant formation of stilbene (mixture of *cis*–*trans* isomers) with only traces of 9 formed. Serendipitously, we found that the outcome of this reaction is effected dramatically by solvent. Using DCM/*n*-hexane (6 : 1) as the reaction medium, stilbene formation was negligible (*ca.* 3%) and 9 could be formed in moderate yield using BEt_3_ (Fig. 4). We attribute this disparity to the solvent effecting the equilibrium position between 8 and the thiiranium cation essential for fluorination.^8^ Notably, the use of the more soluble (than CsF) fluoride source [NMe_4_]F (in the absence of any borane) under identical conditions led to significant stilbene formation (2 : 1 ratio of stilbene : 9) in contrast to the outcome using CsF/BEt_3_. The reaction of Ph_3_CCl with CsF in CHCl_3_ catalysed by either BEt_3_ or 1 proceeded in moderate to good yield. Benzoyl chloride proved to be more challenging, with 1 as the catalyst fluorination proceeded to only *ca.* 5% conversion. However, using 10 mol% BEt_3_ benzoyl fluoride was formed in good yield.

### Enantioselective fluorination studies

**Scheme 2:**
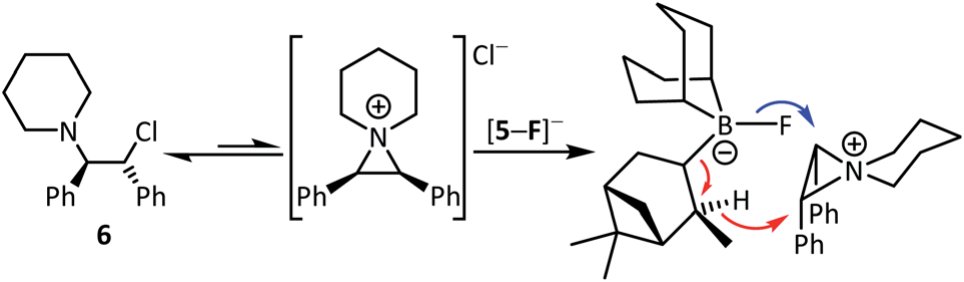
Fluorination of 6 (and 8) with Cs[5-F] (blue arrow) competes with Midland type reduction (red arrows).

One attractive feature of using boranes as CsF phase transfer fluorination catalysts is the ready accessibility of many enantioenriched boranes.^13^ Herein in proof of principle studies commercially available 4 and 5 were assessed in the enantioselective fluorination of 6 and 8 (which proceed *via* ring opening of the *meso* aziridinium and thiiranium cations, respectively).^8^ While 5 was ineffective as a catalyst in halocarbon solvents, it did function in the presence of MeCN. However, the use of stoichiometric Cs[5-F] in DCM/MeCN mixtures while leading to formation of 7 and 9, resulted in no e.e. being observed by chiral HPLC analysis. Furthermore, significant amounts of hydrodehalogenation also was observed using Cs[5-F] alongside formation of 7/9, possibly *via* a mechanism related to the Midland reduction (Scheme 2).^13*c*^

**Fig. 5:**
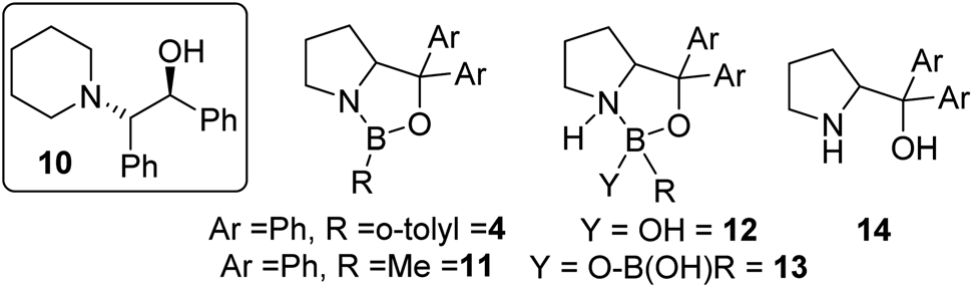
Inset left, amino alcohol 10, middle and right, structures of compounds present in commercial sourced CBS catalyst.

The use of commercially available CBS catalyst 4 (0.5 M in toluene) also was explored as it is not prone to loss of hydride. Surprisingly (given its low calculated fluoride affinity), as received 4 effectively catalysed fluorination of 6 with CsF and led to appreciable e.e. in 7 (maximum e.e. observed using commercial 4 was in CHCl_3_ at 20 °C = 30% e.e.).^15^ In addition to 7, *ca.* 5% of the β-amino-alcohol, 10 (inset Fig. 5), was formed at early stages of the reaction, attributed to the presence of low quantities of water that leads to hydroxide transfer to 6.^16^ A range of CBS catalysts were bought or made (see ESI†) and used as crude mixtures (as per CBS-catalysed hydroboration procedures). However, none gave better e.e. than commercial 4 in the catalytic fluorination of 6 with CsF. Notably, commercial CBS catalyst 11, supplied as a solid, only enabled fluorination after an induction period. Due to this disparity detailed analysis of the commercial batches of 4 and 11 was performed. This revealed a number of impurities present at significant levels (up to 30% by ^11^B NMR spectroscopy), including resonances consistent with products derived from reaction of 4/11 with water as previously reported (*e.g.*12/13/14; Fig. 5).^17^

**Fig. 6:**
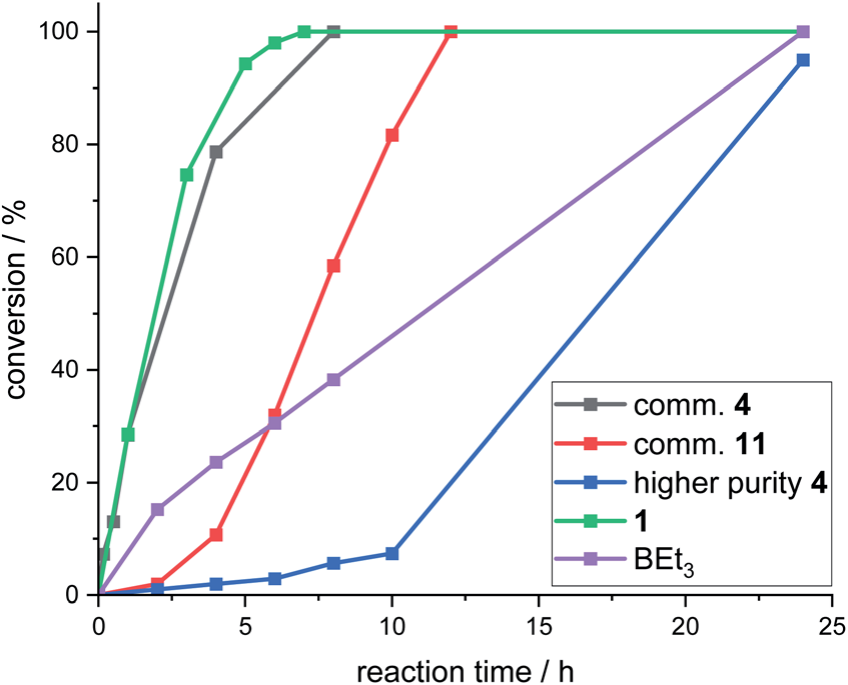
Plots of conversion (by ^1^H NMR integration of 7*vs.*6) *vs.* time for the fluorination of 6 with CsF catalysed by either 10 mol% 1, BEt_3_, 4 (commercial and independently synthesised) and 11 (commercial).

Attempts were made to isolate high purity CBS catalysts for further studies. This proved challenging, but the formation of several in significantly higher purity (*ca.* 90–99% purity) than the commercial material was achieved.^18^ These higher purity CBS catalysts gave worse outcomes than using commercial batches of 4 in the fluorination of 6 with CsF. In addition, all >90% purity CBS catalysts (including independently synthesised 4, termed “higher purity 4”) displayed an induction period before significant fluorination occurred (Fig. 6). This indicated that CBS catalysts are actually pre-catalysts for phase transfer fluorination. It should be noted that 1 and BEt_3_ did not display induction periods during the fluorination of 6 under identical conditions. Attempts were made to elucidate the structure of the catalytically active species derived from CBS pre-catalysts under fluorination conditions, however this study was inconclusive, and these results can be found in the ESI.†

While this work with CBS (pre)catalysts provides proof of principle that enantioselective borane phase transfer fluorination catalysis is feasible, the ill-defined and complex mixtures produced using CBS (pre)catalysts under these conditions is a complicating factor presumably contributing to the maximum e.e. being 30%, despite using multiple CBS (pre)catalyst structures. This highlights the importance of using borane catalysts that are robust under these conditions to allow for rational control of reactivity (note under these fluorination conditions both 1 and BEt_3_ show no observable decomposition, *e.g.* by protodeboronation or BPin hydrolysis).

### MF binding studies

To understand why only certain borane/MF combinations are effective fluorination catalysts, their ability to form M[fluoroborate] salts was explored. With BEt_3_ and with 1/2 no change to the NMR spectra (including the amount of borane observed in solution *vs.* an internal standard) was observed on addition to KF suspended in CHCl_3_, consistent with the higher lattice enthalpy of KF relative to CsF (KF = 194.4 kcal mol^−1^ and CsF = 178.7 kcal mol^−1^).^19^ The absence of any fluoroborate formation is presumably why there is no fluorination of 6 using these boranes and KF. In contrast, combining BEt_3_ with CsF formed the fluoroborate in a range of solvents (Table 2). Notably, the NMR spectra for Cs[FBEt_3_] were significantly different in DCM/CDCl_3_ (entries 1 and 2) compared to those in MeCN (entry 3), with this solvent dependence attributed to a different aggregation of the Cs[FBEt_3_] salt. This is supported by DOSY NMR studies which indicated [FBEt_3_]^−^ was a monomer in MeCN, but exists in larger aggregates in DCM ([Cs(FBEt_3_)]_*n*_ with *n* >1, *vide infra*). This is attributed to MeCN being more effective at ligating Cs^+^ than halocarbon solvents, breaking up Cs_*n*_(μ-F)_*n*_ (*n* >1) units. A related process would explain the addition of [2.2.2]-cryptand (1.25 eq.) to Cs[FBEt_3_] in halocarbon solvents resulting in a considerable shift in fluoroborate resonances (compare entries 2 and 4). The cryptand by strongly binding Cs^+^ will weaken the B–F⋯Cs interaction which will increase the B–F bond strength (*vide infra*).

**Table 2:** Select ^11^B and ^19^F chemical shifts (in ppm) of mixtures of boranes with CsF in various solvents. Crypt = [2.2.2]-cryptand; n.r.: not resolved; n.d.: not detected

#	Conditions	*δ*_11B_	*δ*_19F_	^1^ *J*_BF_/Hz
1	BEt_3_/CsF/CDCl_3_	11.2	−148.3	n.r.
2	BEt_3_/CsF/DCM	9.8	−148.3	n.r.
3	BEt_3_/CsF/MeCN	5.4	−178.9	63
4	BEt_3_/CsF/DCM/crypt	5.2	−192.0	89^a^
5	BEt_3_/CsF/MeCN/crypt	4.5	−190.2	88
6	5/CsF/MeCN	4.1	−153.6	80
7	1/CsF/CDCl_3_	n.d.	n.d.	n.d.
8	1/CsF/MeCN	7.4	−130.2	72
9	1/CsF/CDCl_3_/crypt	2.9	−144.4	n.r.

a No ^1^*J*_BF_ resolved when run in CDCl_3_, thus data in DCM reported.

As expected, [2.2.2]-cryptand more strongly ligates Cs^+^ than MeCN (confirmed by addition of [2.2.2]-cryptand to a MeCN solution of Cs[FBEt_3_] causing a shift from *δ*_19F_ = −178.9 to *δ*_19F_ = −190.2 (entry 3 *vs.* 5) indicating displacement of MeCN from Cs^+^ by cryptand). The different chemical shifts and coupling constants observed suggests significantly different B–F bond strengths in these systems, presumably due to different Cs⋯F–B interactions. Therefore Cs^+^ ligation will effect not just the energetics of solid to solution phase transfer of CsF using boranes, but also the ability of the formed Cs[FBR_3_] to act as a nucleophilic source of fluoride. The NMR data indicate that CsF/BR_3_ in halocarbon solvents (*e.g.* entries 1/2) should be the most nucleophilic source of fluoride using BEt_3_ as catalyst, due to the downfield shifted ^11^B resonance (which is generally associated with less electron density located at boron which would correlate with a weaker B–F bond in this context). This is consistent with the catalytic fluorination results where halocarbon solvents gave better outcomes than using MeCN.

Borane 5 also was studied as it is a triorganoborane with the same calculated fluoride affinity as BEt_3_ but a different environment around the boron centre, which significantly impacts its performance in catalysing nucleophilic fluorination (*vide supra*). Compound 5 showed no propensity to bind CsF in halocarbon solvents (by NMR spectroscopy) in contrast to BEt_3_, consistent with the disparate catalytic nucleophilic fluorination performance observed in DCM. This further confirms that calculated fluoride affinity values must be used with caution for predicting reactivity when there is a coordinating cation present. Using DCM/MeCN mixtures or neat MeCN did enable formation of the fluoroborate, Cs[5-F] (Table 2 entry 6), consistent with the observation of fluorination using this borane in these solvents. This again indicates that interaction of Cs^+^ with MeCN provides a significant contribution to the solubilisation of CsF.

**Fig. 7:**
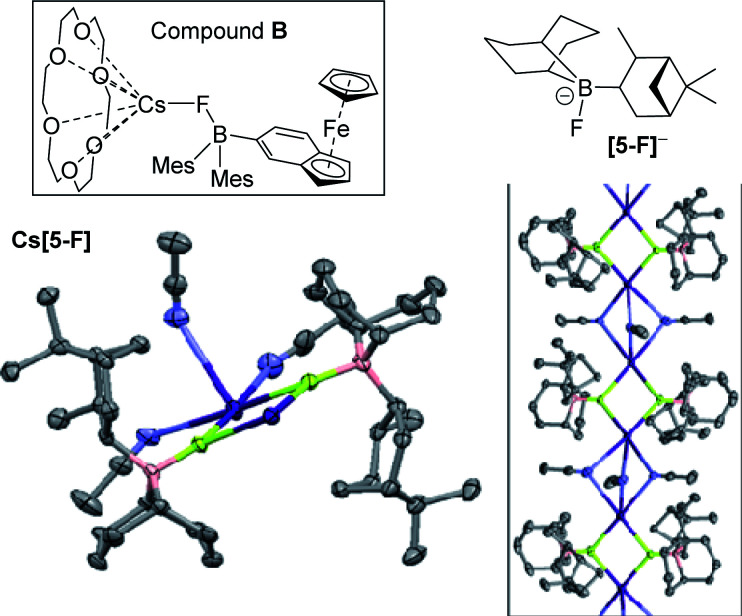
Top, compounds B and [5-F]^−^. Bottom left, one Cs_2_(FBR_3_)_2_ unit. Inset right, the extended 1D polymeric structure of MeCN solvated Cs[5-F]. Yellow = F, pink = B, purple = Cs, blue = N, grey = C. Selected bond distances (Å) and angles (°) in Cs[5-F]: B–F = 1.524(5) and 1.526(6); Cs–F = 2.862(3)–2.945(3); Cs–N = 3.190(5)–3.245(5); B–C = 1.616(9)–1.650(8); F–Cs–F = 73.97(8)–75.19(8); Cs–F–Cs 104.32(9)–106.51(9). Sum of C–B–C angles = 335.69 and 336.09.

Single crystals of Cs[5-F] were obtained from a saturated MeCN solution at −25 °C with its solid state structure consisting of {Cs_2_(FBR_3_)_2_} units propagated into a 1D-coordination polymer by three acetonitrile molecules bridging two adjacent caesium centres (Fig. 7, inset right). In Cs[5-F] each Cs^+^ cation is interacting with only five Lewis base donor atoms. Note the only other close contacts involving Cs^+^ in the extended structure of Cs[5-F] are C–H⋯Cs^+^ interactions with the shortest being 3.133 Å, these are presumably significantly weaker interactions than those involving N⋯Cs^+^/F⋯Cs^+^/O⋯Cs^+^. Solid state structures of Cs[FBR_3_] salts are rare, but Aldridge and co-workers have reported a monomeric example, (18-crown-6)Cs–F-Baryl_3_ (B; Fig. 7), in which Cs^+^ is interacting with seven Lewis base donor atoms.^20^ A comparison of the two structures is informative with different degrees of aggregation/Cs^+^ ligation significantly effecting key bond distances, in B: B–F = 1.496(5) Å and Cs⋯F = 3.034 Å, whereas in Cs[5-F]: B–F = 1.524(5) Å and Cs⋯F = 2.945(3) Å. This is consistent with: (i) the presence of a more Lewis acidic caesium centre more strongly interacting with the B–F unit, thereby reducing the B–F bond strength; (ii) the observed impact of caesium ligation (*e.g.* with cryptands – *vide infra*) on the ability of fluoroborates to transfer fluoride from boron to carbon electrophiles. The low formal coordination number of Cs^+^ in Cs[5-F] may explain the disparity in reactivity between 5 and BEt_3_ towards CsF, particularly in halocarbon solvents. The larger hydrocarbyl groups in 5 (relative to Et in BEt_3_) may prevent additional interactions to Cs^+^ (*e.g.* formation of higher Cs_*n*_F_*n*_ aggregates containing additional Cs⋯FB interactions) thus leading to unfavourable solvation energetics (and thus no reaction) when 5 is combined with CsF in halocarbon solvents. This again emphasises that appropriate ligation of caesium in Cs[F–BR_3_] is vital alongside the appropriate borane fluoride affinity in enabling borane catalysed phase transfer fluorinations.

Moving to dioxaborolanes, with ArBPin/CsF combinations only the free ArBPin was visible by NMR spectroscopy in halocarbon solvents, although solid is present in these reactions. Assessing these mixtures by NMR spectroscopy using an internal standard revealed a significant decrease in the intensity of ArBPin resonances on addition of CsF for 1 (and 2). This indicates the formation of poorly soluble (in halocarbons) fluoroborate salts derived from 1 (and 2). Thus 1 does react with CsF consistent with its ability to catalyse fluorination. In contrast, no evidence for formation of the fluoroborate was observed on combining CsF/PhBPin (by NMR spectroscopy *versus* an internal standard which showed no decrease in the amount of PhBPin present in halocarbon solutions). The disparity can be attributed to the lower fluoride affinity of PhBPin which will disfavour reaction with CsF and is presumably why PhBPin is a poor catalyst for nucleophilic fluorination of 6.

**Fig. 8:**
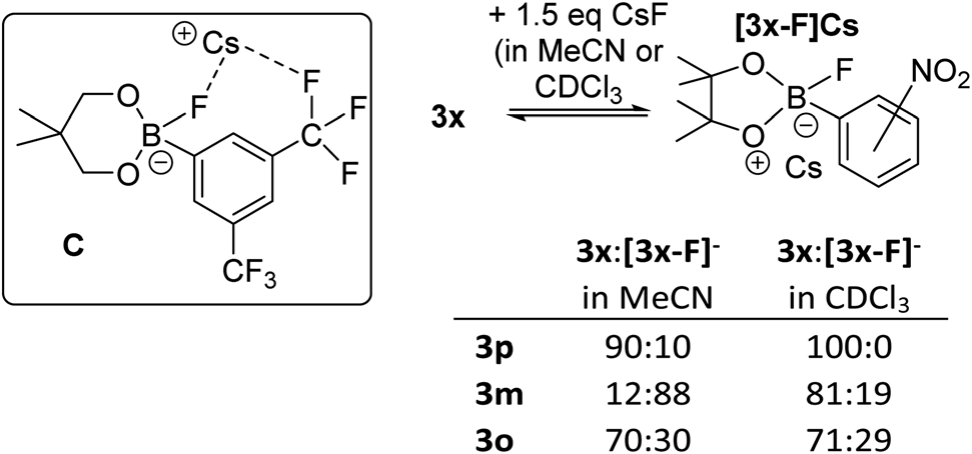
Inset top left: compound C highlighting the intramolecular ArCF_3_⋯Cs interaction. Right, the different propensity to react with CsF for the 3x series.

Notably, the *para*-nitro derivative, 3p, also showed no reaction with CsF in CDCl_3_ (by NMR spectroscopy *versus* an internal standard), despite 3p having an effectively identical calculated fluoride affinity to that for 1. This is consistent with the relatively poor catalytic performance of 3p in the fluorination of 6 (Table 1). Furthermore, in MeCN while 1 is converted significantly to soluble fluoroborates on reaction with CsF (*e.g.*Table 2, entry 8), combining 3p with excess CsF in MeCN led to only *ca.* 10% of Cs[3-F], with 3p being the dominant boron containing species observed. Thus despite a similar calculated fluoride affinity to 1, borane 3p is much less disposed to react with CsF in a range of solvents. We propose that this is due to a sufficiently different (to effect reactivity) interaction with the Cs^+^ cation in the fluoroborates derived from 1 and 3p. This is attributed to intramolecular ArCF_3_⋯Cs^+^ interactions using *meta* substituted 1 persisting in solution, in contrast intramolecular ArNO_2_⋯Cs^+^ contacts are not feasible in *para* substituted [3p-F]Cs (as Cs⋯F–B contacts are expected to be preferred based on the structures of B and C). Multiple short ArCF_3_⋯Cs contacts are present in the solid-state structure of the closely related salt Cs[FB(neop)(*m*-C_6_H_3_(CF_3_)_2_)] (C; inset Fig. 8),^21^ including intramolecular ArCF_3_⋯Cs contacts. The latter may persist to some extent in halocarbon solution and effect the strength of the interaction between the borane and CsF for *ortho* and *meta* substituted, but not *para* substituted aryl boronate systems.

The importance of intramolecular ligation of Cs^+^ was further indicated by the improved performance of 3o and 3m relative to the *para* derivative 3p in phase transfer fluorination (Table 1). This was consistent with the NMR studies with 3m and 3o forming *ca.* 20% and 30% of the fluoroborate in chloroform, respectively, and *ca.* 88% and 30% formation of the fluoroborate in acetonitrile, respectively (Fig. 8, right). This is despite the slightly lower fluoride affinity values for 3m and 3o relative to 3p (Table 1). Again this indicates that the FIA is only one of several factors that need to be considered for identifying effective borane based MF phase transfer fluorination catalysts. The ability of borane substituents to interact with Cs^+^ being another important factor enabling phase transfer, particularly for lower FIA boranes (*e.g.* compare the reactivity of 3o and PhBPin). A similar effect also was observed when comparing the *ortho* and *para* isomers of ((CF_3_)C_6_H_4_)BPin, 15o and 15p. Borane 15o was significantly more active as a catalyst in the fluorination of 6 with CsF (conditions as per Table 1, 43% 7 formed after 8 h) compared to 15p (*ca.* 10% 7 formed after 8 h). This is consistent with 15o forming *ca.* 15% [15o-F]Cs in chloroform whereas 15p displayed no propensity to bind CsF under identical conditions. Note, 15o and 15p have effectively identical calculated FIA values (97 and 96 kJ mol^−1^, respectively) again indicating that the ability of borane substituents to ligate Cs^+^ plays an important role facilitating CsF phase transfer. While intramolecular ligation of Cs^+^ in the Cs[fluoroborate] salt is clearly beneficial for enhancing the phase transfer of CsF by boranes with *ortho*/*meta* CF_3_ and NO_2_ groups, stronger Lewis basic *ortho* substituents actually lead to poorer outcomes. For example, using ((*o*-NH_2_)C_6_H_4_)BPin led to much slower fluorination of 6 (68% 7 formed after 48 h).

**Scheme 3:**
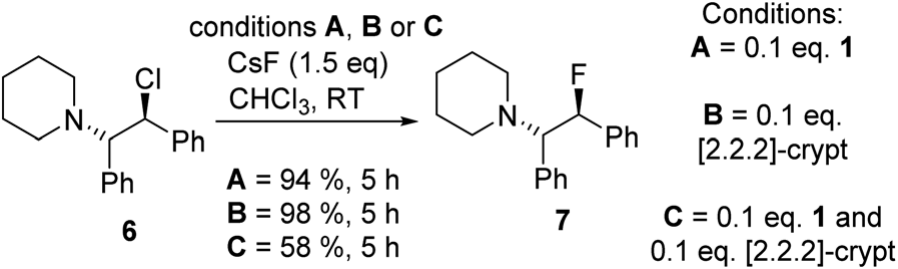
Effect of cryptand/borane on phase transfer fluorination with CsF.

To probe the consequences of caesium ligation in the BPin systems further, the effect of [2.2.2]-cryptand on Cs[fluoroborate] reactivity was explored. A mixture of 1/[2.2.2]-cryptand and excess CsF gave a halocarbon soluble product (Table 2, entry 9), with *δ*_11B_ = 2.9 and *δ*_19F_ = −144.4, albeit both resonances being broad with no resolved B–F coupling. The upfield shift (relative to entry 8) in *δ*_11B_ suggests adding cryptand leads to stronger B–F binding, presumably by weakening the Cs⋯F–B interaction. This should disfavour nucleophilic fluorination by the fluoroborate, which indeed is what was observed. Specifically, the use of a 1 : 1 combination of 1/[2.2.2]-cryptand retarded fluorination of 6 with CsF (relative to fluorination of 6 using just 1 or using just [2.2.2]-cryptand, Scheme 3) despite CsF phase transfer being observed to form the fluoroborate in all cases. Thus [2.2.2]-cryptand more effectively sequesters Cs^+^ leading to a relatively strong B–F bond in the fluoroborate that is a poorer nucleophilic source of fluoride. This clearly highlights that careful control of caesium ligation is vital to enable binding of CsF (favoured by stronger binding of Cs^+^) but also to maintain a significant Cs⋯F–B interaction that labilises the B–F bond (favoured by weaker binding of Cs^+^).

## Conclusions

Despite the high fluorophilicity of boron, certain organoboranes and boronate esters can be employed as CsF phase-transfer nucleophilic fluorination catalysts. Chiral induction during fluorination with borane catalysts also was demonstrated as proof of principle (up to 30% e.e.), however limited catalyst stability under these reaction conditions precluded realising high e.e. with CBS systems, highlighting the importance of using boranes robust to fluorination conditions. Regarding the factors controlling effective catalysis, as expected, nucleophilic fluorination reactivity is impacted by B–F bond strength, which is dependent on borane Lewis acidity towards fluoride. Sufficient fluoride affinity favours the borane reacting with CsF, however if fluoride affinity is too high the resultant fluoroborate does not effectively transfer fluoride to electrophiles. Importantly, nucleophilic fluorination is most effective under conditions that provide sufficient ligation of Cs^+^ to enable solid to solution phase transfer. However, avoiding too effective a ligation of Cs^+^ is also vital, as good ligation of Cs^+^ weakens the Cs⋯F–B interaction, strengthening the B–F bond and thereby leading to less reactive fluoroborates. In terms of predictability, boranes with calculated fluoride affinity of 95–120 kJ mol^−1^ (*vs.* Me_3_Si^+^) appear to be suitable candidates as nucleophilic fluorination catalysts, with the caveat that other factors (*e.g.* borane stability under the reaction conditions/forming the correct fluoroborate aggregation/Cs^+^ ligation level in solution) are also important to consider. Finally, weak intramolecular ligation of Cs^+^ by borane substituents appears an effective method to enable lower FIA boranes to achieve CsF phase transfer and nucleophilic fluorination. When the various prerequisites are met, simple boranes are effective catalysts for nucleophilic fluorination using CsF, including to access useful products (*e.g.* β-fluoroamines).

## Data availability

Full experimental procedures, NMR spectra, DFT and crystallographic details/data are provided in the ESI.†

## Author contributions

MI and SK conceived the research concept and aims and analysed all data. SK performed the majority of the synthetic work and the majority of the analytical components of this project. MP performed preliminary investigations on commercial CBS, BEt_3_ and 5 catalysed fluorinations. KY performed all the computational investigations. MU collected and solved the crystal structure. SK and MI drafted, reviewed and edited the manuscript.

## Conflicts of interest

There are no conflicts to declare.

## Supplementary Material

SC-013-D2SC00303A-s001

SC-013-D2SC00303A-s002
