# Supplementary material for: Developing organoboranes as phase transfer catalysts for nucleophilic fluorination using CsF
Source: Chem Sci. 2022 Feb 9;13(9):2661–8. doi: 10.1039/d2sc00303a (PMC8890113; doi:10.1039/d2sc00303a)
Supplement: SC-013-D2SC00303A-s001 [file SC-013-D2SC00303A-s001.pdf]

Electronic Supplementary Material (ESI) for Chemical Science.
This journal is © The Royal Society of Chemistry 2022

Electronic Supporting Information for

Developing organoboranes as phase transfer catalysts for nucleophilic fluorination using CsF.

Contents

General Remarks 2

Reaction of $[Me_4N]$[fluoroborate]s with $[Ph_3C][B(C_6F_5)_4]$ 3

Syntheses of $[Me_4N][FBR_3]$ 3

Fluorination of $[Ph_3C][B(C_6F_5)_4]$ with $[Me_4N][FBR_3]$ 3

Nucleophilic Fluorination with CsF 7

Fluorination of **6** with BEt_3 or aryl–Bpins 7

Fluorination of activated haloalkanes, general procedure: 7

Screening of conditions 7

Catalyst optimisation experiments: 8

Enantioselective Fluorination Studies 10

Synthesis of chiral B-based phase-transfer catalysts 10

CBS catalysts as phase-transfer fluorination catalysts 12

Syntheses of CBS catalysts 12

Fluorination of **6** under CBS phase transfer catalysis 16

MF binding studies 20

DOSY studies on $Cs[FBEt_3]$ 20

Complexation of **1** with F^- 22

Crystallographic Data 23

Computational Details 24

Plots of NMR spectra 48

Plots of HPLC traces 74

References 77

General Remarks

Handling of air- and moisture-sensitive reagents was carried out under an inert atmosphere using either standard Schlenk techniques or an *MBraun* glovebox (< 0.1 ppm H_2O/O_2).

Solvents were obtained from an *Inert PureSolv MD5* solvent purification system. $CHCl_3$ was distilled over CaH_2 and stored over 3 Å molecular sieves protected from air and direct sunlight.

Reagents were, unless otherwise stated, purchased from commercial sources and used as received. CsF was obtained from *Sigma Aldrich* and dried at 120 °C for 6 h *in vacuo*. The dried material was then finely ground and stored in the glovebox. KF was obtained from *Honeywell* (>99%, spray dried), finely ground and stored in the glovebox without any further purification.

NMR spectra (^{1}H, ^{2}H, $^{11}B\{^{1}H\}$, ^{11}B, $^{13}C\{^{1}H\}$, $^{19}F\{^{1}H\}$, and ^{19}F NMR) were recorded on *Bruker Avance III 400*, *Bruker Avance III 500 MHz* or *Bruker PRO 500 MHz* spectrometers. Chemical shifts are reported as dimensionless *δ* values and are frequency referenced relative to residual protio-solvent signals in the NMR solvents for ^{1}H and $^{13}C\{^{1}H\}$, while ^{11}B and ^{19}F shifts are referenced relative to external $BF_3{\cdot}Et_2O$ and C_6F_6, respectively. It should be noted that the very broad signals observed at ca. 0 ppm in the ^{11}B NMR spectra are due to the use of borosilicate glass NMR tubes and boron containing parts in the NMR cavity. These are not coincident with broad compound signals. Coupling constants *J* are given in Hertz (Hz) as positive values regardless of their real individual signs. Unless otherwise stated NMR spectra were recorded at 20 °C.

Mass spectrometry was performed by the *Scottish Instrumentation and Resource Centre for Advanced Mass Spectrometry* (*SIRCAMS*) of the University of Edinburgh using either electron impact (EI) or electrospray ionisation (ESI) techniques. Accurate masses are calculated using the most abundant isotopes of each element.

Column chromatography was performed on a *Teledyne Isco CombiFlash 100* instrument using *Advion-Interchim* columns (spherical silica, 25 µm; or C18 silanised silica, 30 µm, for RP chromatography).

Enantioselective HPLC analysis was performed on an *Agilent 1100 Series* instrument using a *Daicel Chiralpak IC* analytical column. Solvents used were of *HPLC grade*.

Karl Fischer titration was performed semi-automatically on a *Metrohm 899* coulometer using a generator cell without diaphragm. *Hydranal™ Coulomat AG* was used as reaction medium. Unless otherwise stated, samples were analysed neat and experiments were run in triplicates.

Yields of known compounds refer to material of >95% purity by ^{1}H NMR spectroscopy.

Reaction of $[Me_4N]$[fluoroborate]s with $[Ph_3C][B(C_6F_5)_4]$

Syntheses of $[Me_4N][FBR_3]$

Synthesis of $[Me_4N][FBEt_3]$

To an ampoule charged with anhydrous $[Me_4N]F$ (500 mg, 5.36 mmol) was added dry THF (20 mL) and BEt_3 (1M in hexane, 5.90 mL, 5.90 mmol). The colourless suspension slowly turned into a homogeneous solution and was stirred at room temperature for 2 h. All volatiles were removed *in vacuo* and $[Me_4N][FBEt_3]$ was obtained as a colourless powder.
Yield: 870.8 mg (4.56 mmol, 85%)

^{1}H NMR (400 MHz, THF) δ = 3.23 (s, 12 H), 0.66 (t, J = 7.8 Hz, 9H), 0.03 (q, J = 7.7 Hz, 6H).
^{11}B{^{1}H} NMR (128 MHz, THF) δ = 7.2 (br).
^{19}F{^{1}H} NMR (376 MHz, THF) δ = –183.4 (br).

Synthesis of $[Me_4N][FBPh_3]$

To an ampoule charged with anhydrous Me_4NF (93 mg, 1 mmol) was added dry MeCN (4.0 mL) and a suspension was obtained. To the suspension was added BPh_3 (4 mL, 0.25 M in THF) and the suspension turned into a homogeneous solution right after the addition. The solution was stirred at room temperature for 30 minutes. Then the solvent was removed *in vacuo* and $[NMe_4][FBPh_3]$ was obtained as a colourless solid.
Yield: 200 mg (0.60 mmol, 60%)

^{1}H NMR (400 MHz, MeCN/THF 1:1) δ = 7.36 (dd, J = 7.8, 1.5 Hz, 6H), 6.98 (t, J = 7.4 Hz, 6H), 6.89 – 6.79 (m, 3H), 2.75 (s, 12H).
^{11}B{^{1}H} NMR (128 MHz, THF) δ = 3.8 (d, J_{BF} = 74.1 Hz).
^{19}F{^{1}H} NMR (376 MHz, THF) δ = –196.9 (m).

Fluorination of $[Ph_3C][B(C_6F_5)_4]$ with $[Me_4N][FBR_3]$

General Procedure

$$[FBR_3][NMe_4] + [Ph_3C][B(C_6F_5)_4] \xrightarrow[\text{DCM}]{} Ph_3CF + BR_3 + [NMe_4][B(C_6F_5)_4]$$

An NMR tube with a J. Young's valve was charged with $[Me_4N][FBR_3]$ (1 eq) and $[Ph_3C][B(C_6F_5)_4]$ (1 eq). After addition of dry DCM (1.0 mL), the mixture was agitated for 1 h, after which progress of the reaction was controlled by NMR spectroscopy.

Reaction with $[Me_4N][FBEt_3]$

Starting from $[Me_4N][FBEt_3]$ (11 mg, 58 µmol) and $[Ph_3C][B(C_6F_5)_4]$ (53 mg, 58 µmol).

$^{11}B\{^1H\}$ NMR (128 MHz, CH_2Cl_2) δ = 86.7 (s; BEt_3), –16.8 (s; $B(C_6F_5)_4$)
$^{19}F\{^1H\}$ NMR (376 MHz, CH_2Cl_2) δ = –126.6 (s; Ph_3CF), –133.1 (ψd), –163.6 (ψt), –167.6 (ψt) (all $[B(C_6F_5)_4]^-$). Data for Ph_3CF in accord with literature.[S1,S2]

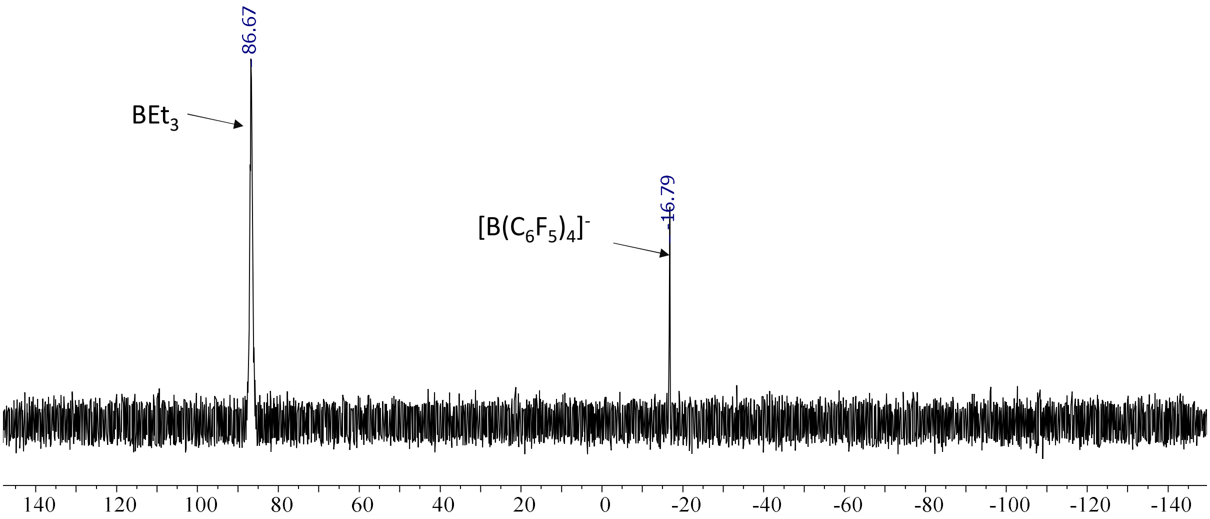

Figure S1: In-situ $^{11}B\{^1H\}$ NMR spectrum (128 MHz) in dichloromethane showing the formation of BEt_3.

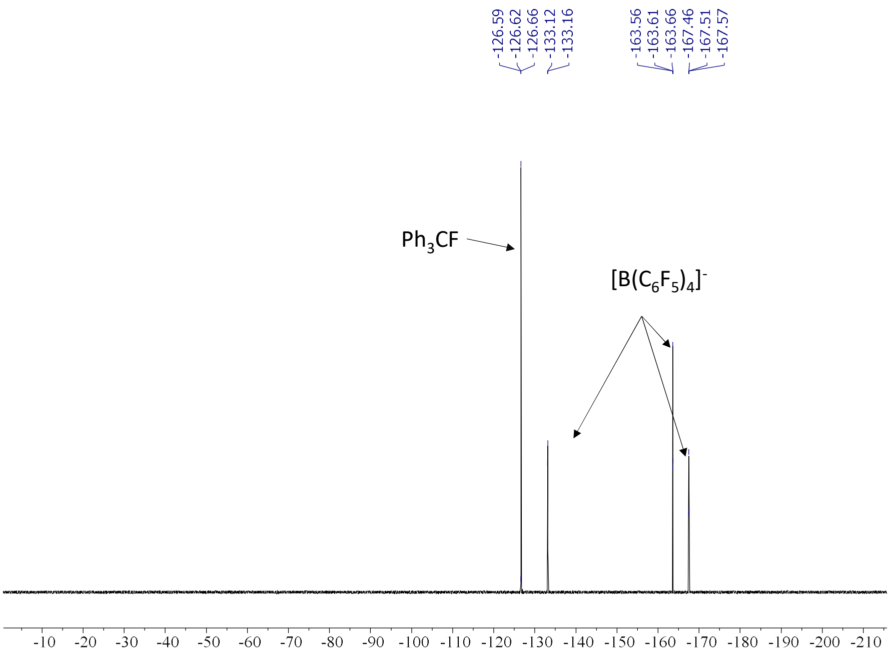

Figure S2: In-situ $^{19}F\{^1H\}$ NMR spectrum (376 MHz) in dichloromethane showing the formation of Ph_3CF.

Reaction with [Me_4N][$FBPh_3$]

Starting from [Me_4N][$FBPh_3$] (46.9 mg, 140 µmol) and [Ph_3C][$B(C_6F_5)_4$] (129 mg, 140 µmol).

^{11}B{^{1}H} NMR (128 MHz, CH_2Cl_2) *δ* = –16.7 (s) ([$B(C_6F_5)_4$]$^{-}$).
^{19}F NMR (376 MHz, CH_2Cl_2) *δ* = –126.6 (s) (Ph_3CF), –133.1 (ψd), –163.6 (ψt), 167.5 (ψt) ([$B(C_6F_5)_4$]$^{-}$).

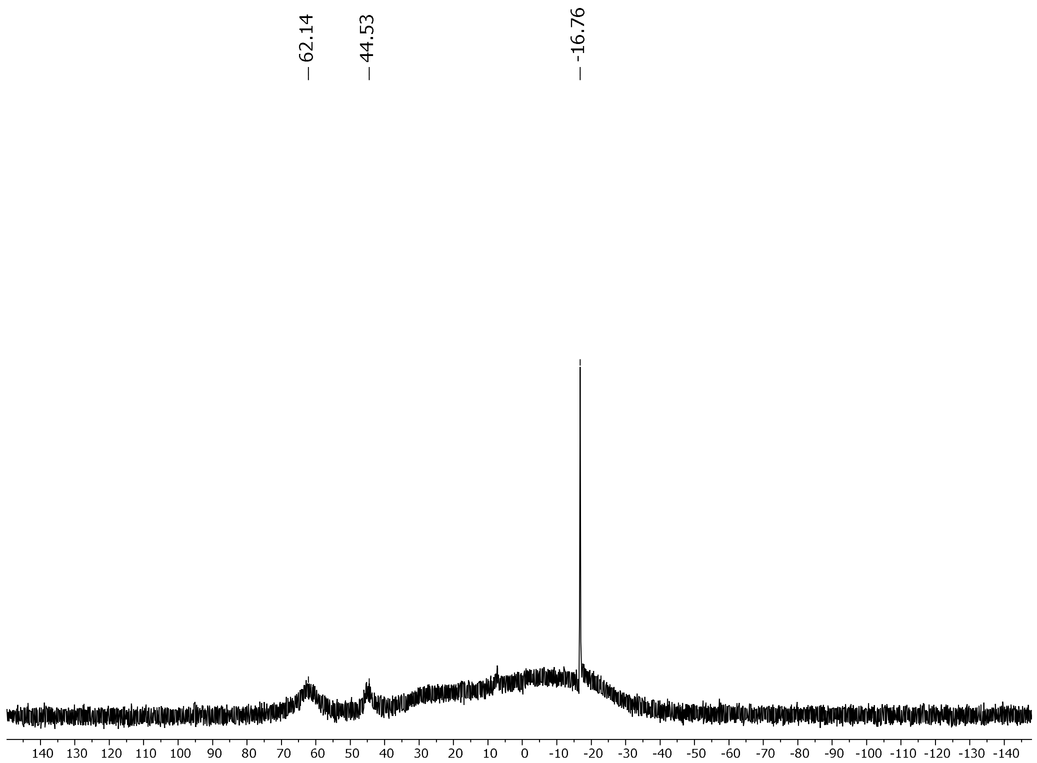

Figure S3: In-situ ^{11}B{^{1}H} NMR spectrum (128 MHz) in dichloromethane showing the consumption of [$FBPh_3$]$^{-}$. The major new resonance at +62 ppm is in the region expected for a tri-aryl borane.

In reactions with BPh_3 / [$FBPh_3$]$^{-}$ minor impurities also are observed, this is attributed to the greater propensity of the B-Ph derivative to undergo protodeboronation when exposed to minor protic impurities, e.g. [HF_2]$^{-}$ / H_2O.

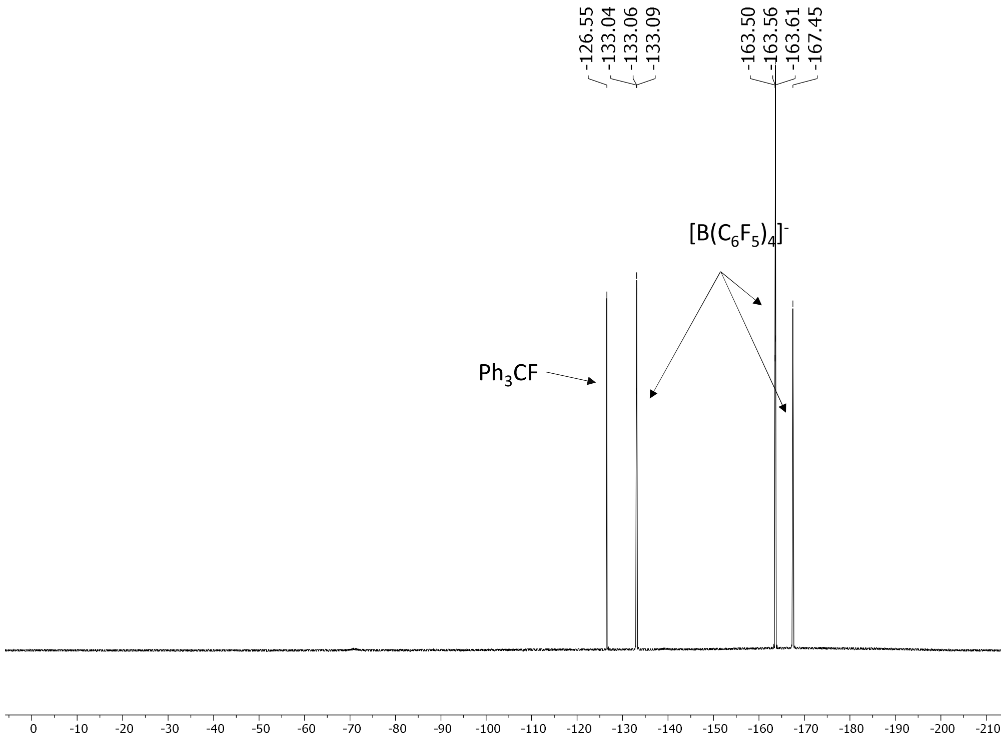

Figure S4: In-situ $^{19}F\{^1H\}$ NMR spectrum (376 MHz) in dichloromethane showing the formation of Ph_3CF.

Nucleophilic Fluorination with CsF

Fluorination of **6** with BEt_3 or aryl–Bpins

Fluorination of activated haloalkanes, general procedure:

A Schlenk tube was charged with the respective haloalkane (1.0 eq), the aryl–Bpin (0.1 eq, if used) and CsF (1.5 eq, finely ground, stored in a glove box). After addition of dry $CHCl_3$ (to give a 0.5 M solution), and BEt_3 solution (if used), the reaction mixture was stirred at 1000 rpm. Progress of the reaction was monitored by subjecting aliquots of the mixture to ^{1}H NMR spectroscopy (for Ph_3CF and PhC(O)F progress was monitored by $^{13}C\{^{1}H\}$ NMR spectroscopy). After complete conversion, the reaction mixture was filtered through Celite®, eluting with EtOAc. The eluate was evaporated to dryness and the residue adsorbed on silica. The desired product was isolated by column chromatography. Note: After fluorination of chlorotriphenylmethane and benzoyl chloride, column chromatography lead to complete hydrolysis of the product. Thus, conversion was quantified by ^{19}F-NMR spectroscopy, using 1,2-$F_2C_6H_4$ (δ = –138.8 ppm)[S3] as internal standard. To obtain accurate integrals for quantitative analysis, the CHORUS pulse programme[S4] was used.

All products are known and were characterised by ^{1}H-NMR spectroscopy:

rac-1-(2-Fluoro-1,2-diphenylethyl)-piperidine (**7**): *ref.* S5
rac-1-(1,2-Diphenyl-2-fluoroethyl)(methyl)sulfide (**9**): *ref.* S6
Fluorotriphenylmethane: *ref.* S2
Benzoyl fluoride: *ref.* S7

Screening of conditions

Table S1: Influence of different additives on conversion.

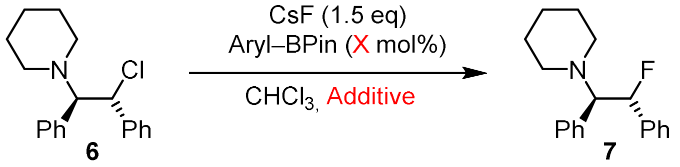

#	*Additive*	*Cat. loading / mol%*	*t* / h	Conversion / %[a]
1	open to air	10	18	50
2	H_2O (0.5 v%)	10	8	<5
3	DMF[b] (5 v%)	10	6	100

[a]: Conversion determined by ^{1}H NMR integration of **6** vs **7**.
[b]: DMF = N,N-dimethylformamide

Catalyst optimisation experiments:

Table S2: Optimisation of the catalyst for the nucleophilic fluorination of **6** with CsF.

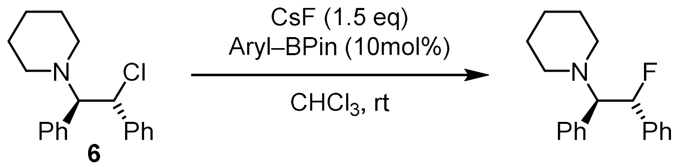

Aryl	*t* / h	Conversion / %	Yield / %
3-Me-C_6H_4–	168	100	82
3,5-$(CF_3)_2C_6H_3$–	7	100	89
3,5-$(CF_3)_2C_6H_3$–	96[a]	<50	--
Ph–	96	<50	--
4-F-C_6H_4–	96	<50	--
4-Cl-C_6H_4–	96	<50	--
4-Br-C_6H_4–	96	<50	--
3,4,5-F_3-C_6H_2–	18	100	73
4-NO_2-C_6H_4–	96	<50	--
3-NO_2-C_6H_4–	18	100	--
2-NO_2-C_6H_4–	8	100	
4-CF_3-C_6H_4–	48	<50	--
2-CF_3-C_6H_4–	24	100	--

[a]: KF (spray dried) used as F^- source

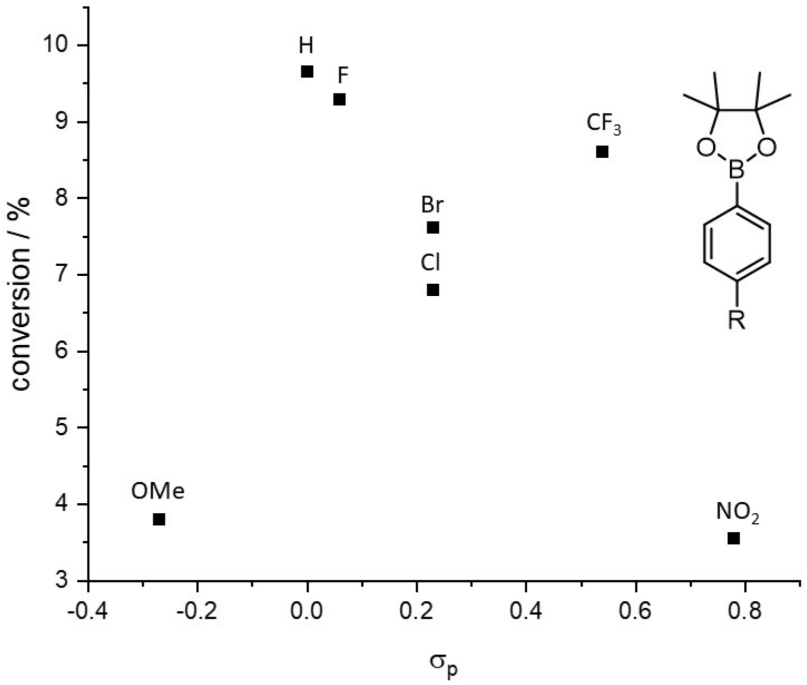

Figure S5: Hammett plot of the catalytic activity of various *para* substituted aryl-BPin derivatives in the nucleophilic fluorination of **6** with CsF to give **7**; conversion estimated by ^1H NMR integration of **7** vs **6** after 5 h reaction time.

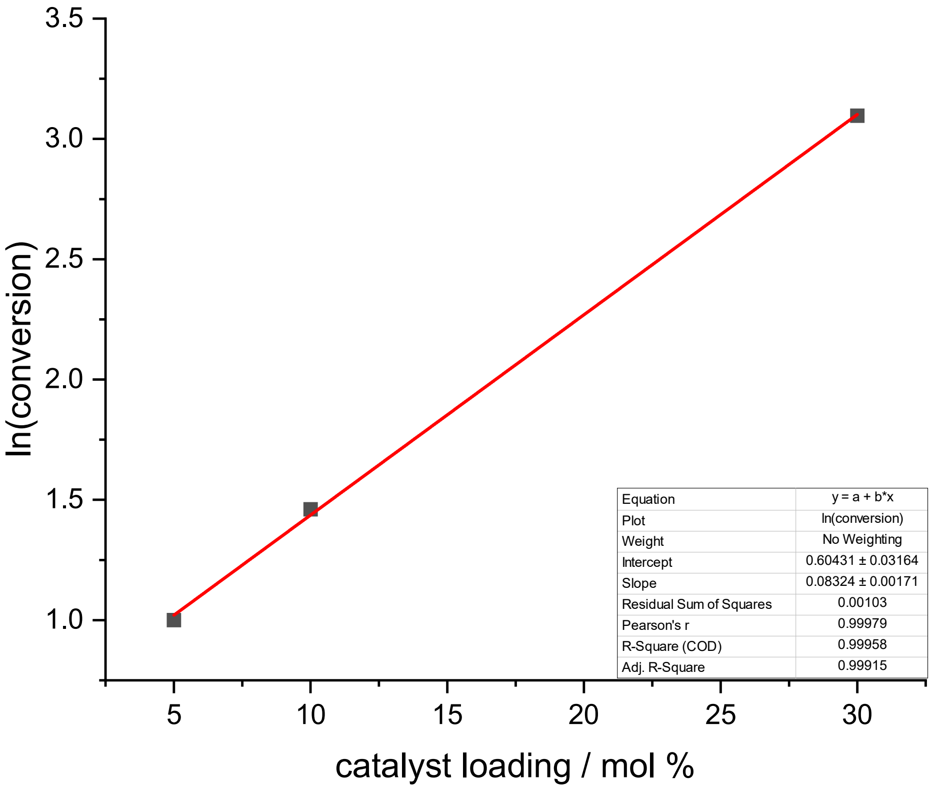

Figure S6: Plot of ln(conversion) vs catalyst loading for the nucleophilic fluorination of **6** with CsF to give **7** using **1** as the catalyst. Conversion estimated by ^{1}H NMR integration of **7** vs **6**.

Enantioselective Fluorination Studies

Synthesis of chiral B-based phase-transfer catalysts

Synthesis of *B*-(3-Pinanyl)-9-borabicyclo[3.3.1]nonane (Alpine-Borane, **5**)

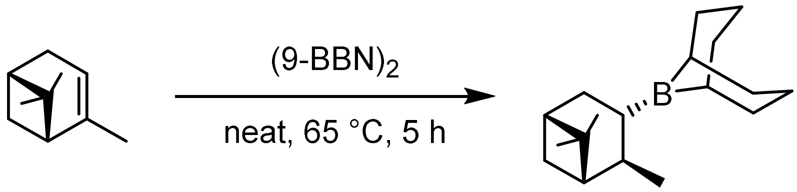

This compound was made according to a literature procedure:[S8] A Schlenk flask was charged with (–)-α-pinene (0.58 mL, 3.67 mmol) and 9-borabicyclo[3.3.1]nonane dimer ($(9\text{-BBN})_2$, 0.41 g, 1.68 mmol). The mixture was vigorously stirred and heated to 65 °C for 5 h. After cooling to room temperature, excess pinene was removed *in vacuo* (40 °C / $5{\cdot}10^{-2}$ mbar); the resulting oil was used without further purification.

^{1}H NMR (500 MHz, CH_2Cl_2) δ = 2.28 (dtd, *J* = 9.6, 6.2, 2.0, 1H), 2.11 (td, *J* = 7.3, 2.2, 1H), 2.07 – 1.99 (m, 1H), 1.96 – 1.84 (m, 7H), 1.82 (ddd, *J* = 7.0, 5.1, 2.2, 1H), 1.75 – 1.65 (m, 8H), 1.32 – 1.23 (m, 2H), 1.20 (s, 3H), 1.14 (s, 3H), 1.05 (d, *J* = 7.1, 3H), 0.74 (d, *J* = 9.4, 1H).
^{11}B NMR (160 MHz, CH_2Cl_2) δ = 82.1.
The data is in accordance with literature reports.[S8]

Synthesis of caesium [alpine-fluoro-borate], Cs[**5–F**]

A glass ampule with a J. Young's valve was charged with **5** (642 mg, 2.48 mmol) and CsF (378 mg, 2.48 mmol). After addition of dry MeCN (10 mL), the mixture was stirred at room temperature for 2 h, giving a cloudy solution. After filtration, the solvent was removed from the clear filtrate under reduced pressure until ca. 2 mL remained. The ampule was tightly sealed and stored at –25 °C for two days to yield colourless crystals of Cs[**5**–F] suitable for X-ray diffraction studies.

^{1}H NMR (500 MHz, CD_3CN) δ = 2.04 – 1.95 (m, 5H, overlaps with CD_3CN), 1.88 – 1.66 (m, 7H), 1.60 – 1.34 (m, 8H), 1.13 (s, 3H), 1.11 (s, 3H), 0.96 (d, *J* = 7.2, 3H), 0.46 – 0.41 (m, 2H).
^{13}C{^{1}H} NMR (126 MHz, CD_3CN) δ = 51.5, 43.9, 40.1, 40.0, 40.0, 35.0, 34.8, 33.8, 33.6, 32.9, 31.5, 28.9, 27.4, 25.3, 23.6.
^{11}B NMR (160 MHz, CD_3CN) δ = 4.0 (d, *J* = 80.0).
^{19}F NMR (471 MHz, CD_3CN) δ = –153.6 (d, *J* = 80.0).
Accurate Mass (EI): calculated for $C_{18}H_{31}BCsF$: 410.15573 (M^-); found: 409.97632

Reaction of Cs[**5–F**] with **6** and **8**

A J. Young's NMR tube was charged with Cs[**5–F**] (33 mg, 78 µmol) and either **6** (19 mg, 65 µmol) or **8** (20 mg, 65 µmol). After addition of dry DCM/MeCN mixture (97:3, 0.6 mL), the tube was agitated at room temperature and progress of the reaction was monitored by ^{1}H NMR spectroscopy.

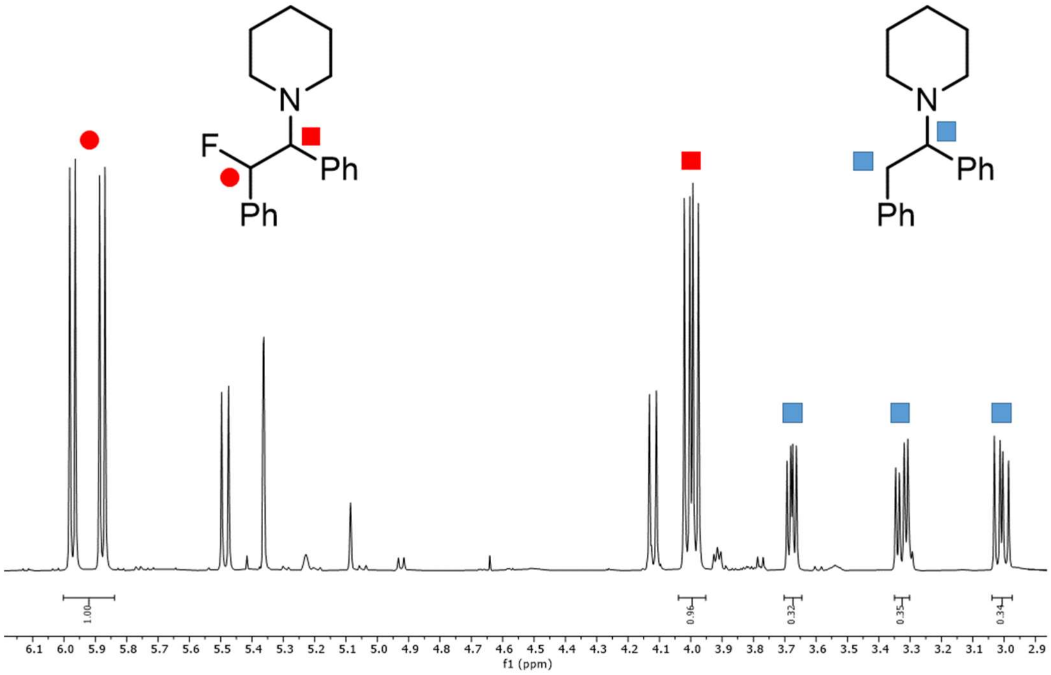

Figure S7: In-situ ^{1}H NMR spectrum (500 MHz) of the reaction of Cs[**5–F**] with **6** to give either **7** or the hydrodehalogenated product via Midland type reduction. Resonances of the side product assigned using Ref. S9

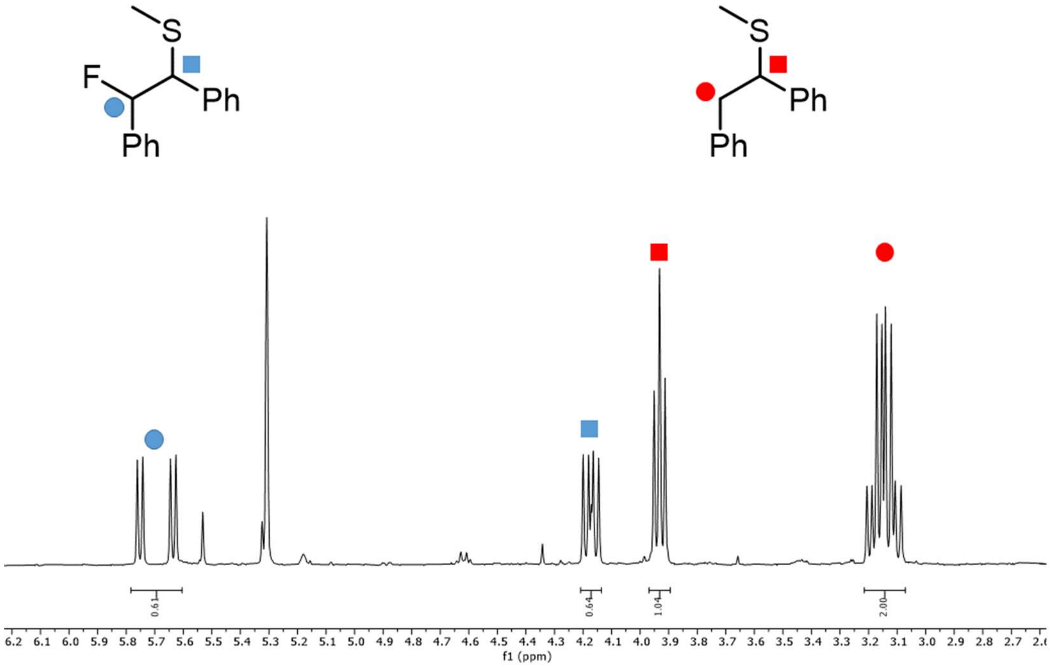

Figure S8: In-situ ^{1}H NMR spectrum (500 MHz) of the reaction of Cs**[5–F]** with **8** to give either **9** or the hydrodehalogenated product via Midland reduction. Resonances of the side product assigned using Ref. S10

CBS catalysts as phase-transfer fluorination catalysts

Syntheses of CBS catalysts

Synthesis of (S)-N-ethylcarbamoylproline methylester

Prepared according to a literature procedure:[S11] To an ice-cooled slurry of K_2CO_3 (2.64 g, 20 mmol) and (S)-proline (2.30 g, 20 mmol) in dry MeOH (40 mL) was added ethyl chloroformate (4.40 mL, 44 mmol). The mixture was allowed to reach room temperature overnight. The reaction mixture was evaporated to dryness with the aid of a rotary evaporator and the colourless residue was taken up in H_2O/EtOAc (1:1; 100 mL). After separation of the layers, the aqueous layer was extracted with EtOAc (3 x 20 mL) and the combined organic layers were washed with brine (20 mL). After drying over Na_2SO_4 and filtration, the solvent was removed under reduced pressure using a rotary evaporator. The remaining colourless oil was the essentially pure mixture of isomers of the desired product and was used without further purification. Yield: 4.00 g (19.9 mmol, >98%).

^{1}H NMR (500 MHz, $CDCl_3$) δ = 4.36 (dd, J = 8.6, 3.5, 1H), 4.29 (dd, J = 8.6, 4.0, 1H), 4.14 (q, J = 7.1, 3H), 4.10 – 3.98 (m, 1H), 3.73 (s, 3H), 3.71 (s, 3H), 3.62 – 3.38 (m, 4H), 2.29 – 2.13 (m, 2H), 2.05 – 1.81 (m, 7H), 1.26 (t, J = 7.1, 3H), 1.19 (t, J = 7.1, 3H). Note: Signals reported are for the mixture of both isomers and no attempts have been made to assign the resonances to the respective molecule.

Synthesis of diaryl-2-pyrrolidinyl-methanols (diarylprolinols)

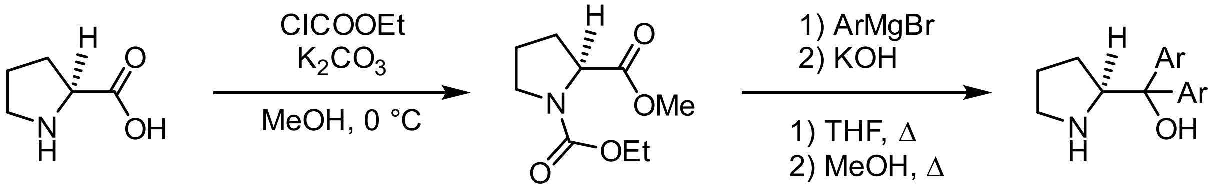

General procedure:

Prepared according to a modified literature procedure:[S11] Mg turnings (3.00 g, 122 mmol) were stirred vigorously *in-vacuo* for 30 min. Dry THF (80 mL) was added, followed by a crystal of I_2 (typically 3-5 mg). The mixture was heated to reflux temperature until the brown colour had disappeared. Ar–Br (89 mmol) was then slowly added dropwise (20 - 30 min). After complete addition, reflux was continued for 1 h and the mixture was then cooled to room temperature. The brown supernatant was removed from the remaining Mg turnings via cannula transfer and added dropwise to an ice cold solution of the crude proline ester (4.0 g, 20 mmol) in dry THF (10 mL). After complete addition, the mixture was stirred in an ice bath for further 4 h and then poured into aqueous NH_4Cl solution (ca. 5 M, 50 mL). After separation of the layers, the aqueous layer was extracted with EtOAc (3 x 30 mL) and the combined organic layers were washed with brine (30 mL) and dried over Na_2SO_4. Following filtration, the solvent was removed with the aid of a rotary evaporator, leaving a highly viscous oil behind which was dried *in vacuo*.

Under an atmosphere of N_2, KOH (finely ground prior to use; 13.2 g, 200 mmol) was added to the oil followed by dry MeOH (40 mL). The resulting slurry was heated to reflux temperature overnight. After cooling to room temperature, most of the solvent was removed with the aid of a rotary evaporator and the remaining slush poured into aqueous NH_4Cl solution (ca. 5 M, 200 mL), adjusting the pH of the solution to 5 – 6. The product was extracted with EtOAc (3 x 30 mL), the combined organic layers were washed with brine (30 mL), dried over Na_2SO_4 and filtered. After evaporation of the solvent from the filtrate, the product was purified by RP chromatography (C18, MeOH) to furnish the respective prolinol as a highly viscous oil that solidified at room temperature.

(S)-Diphenyl-2-pyrrolidinyl-methanol:

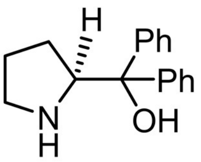

Yield: 4.20 g (16.5 mmol, 83%)

^{1}H NMR (500 MHz, $CDCl_3$) δ = 7.60 – 7.56 (m, 2H), 7.50 (m, 2H), 7.30 (m, 4H), 7.15 (m, 2H), 4.26 (t, J = 7.7, 1H), 3.04 (m, 1H), 2.95 (m, 1H), 1.69 (m, 5H).[S12]

(S)-Bis(3,5-dimethylphenyl)-2-pyrrolidinyl-methanol

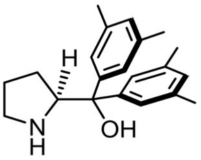

Yield: 3.41 g (11.0 mmol, 55%)

^{1}H NMR (500 MHz, $CDCl_3$) δ = 7.17 (s, 2H), 7.12 – 7.08 (s, 2H), 6.80 (s, 2H), 4.21 (t, J = 7.7, 1H), 3.02 (m, 1H), 2.94 (m, 1H), 2.28 (s, 6H), 2.28 (s, 6H), 1.72 (m, 2H), 1.60 (m, 2H), 1.28 (m, 2H).[S12]

Synthesis of Me-CBS catalysts: General procedure

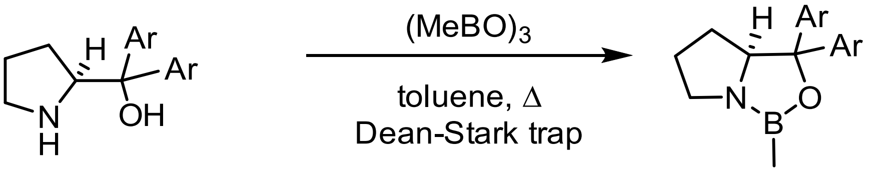

Prepared according to a literature procedure:[S12] Trimethylboroxine (0.66 eq) and the respective prolinol (1.00 eq) were dissolved in dry toluene (0.4 M) and, after stirring at room temperature for 30 min, heated to 175 °C (bath temperature) for 2 h in a Dean Stark apparatus with azeotropic removal of water. A colourless solid formed in the Dean-Stark trap. The reaction mixture was allowed to cool to room temperature and all volatiles were removed under reduced pressure. The residue was dried *in vacuo* at 60 °C for 1 h and then subjected to sublimation (120 °C / $3 \cdot 10^{-2}$ mbar), furnishing the desired oxazaborolidine as a colourless solid.

(4*S*)-2-Methyl-5,5-bis(3,5-dimethylphenyl)-pyrrolidino-[1,2-c][1,3,2]-oxazaborolidine

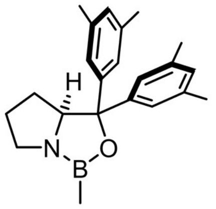

^{1}H NMR (400 MHz, $CDCl_3$) δ = 7.14 (s, 2H), 6.94 (s, 2H), 6.87 (s, 1H), 6.83 (s, 1H), 4.30 (dd, *J* = 9.8, 5.7, 1H), 3.35 (ddd, *J* = 10.5, 8.0, 6.6, 1H), 3.04 (ddd, *J* = 10.5, 8.0, 6.3, 1H), 2.30 (s, 6H), 2.28 (s, 6H), 1.80 – 1.71 (m, 2H), 1.64 – 1.52 (m, 1H), 0.85 – 0.76 (m, 1H), 0.36 (s, 3H).[S12]

^{11}B{^{1}H} NMR (160 MHz, $CDCl_3$) δ = 34.7.

(4*S*)-2-Methyl-5,5-diphenyl-pyrrolidino-[1,2-c][1,3,2]-oxazaborolidine (**11**)

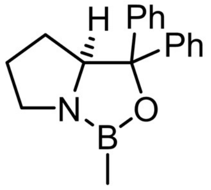

(**11**)

^{1}H NMR (400 MHz, $CDCl_3$) δ = 7.56 – 7.50 (m, 2H), 7.37 – 7.16 (m, 8H), 4.35 (dd, *J* = 9.8, 5.8, 1H), 3.36 (ddd, *J* = 10.6, 8.0, 6.3, 1H), 3.05 (ddd, *J* = 10.5, 8.5, 6.0, 1H), 1.88 – 1.66 (m, 2H), 1.71 – 1.56 (m, 1H), 0.83 (dq, *J* = 12.2, 9.6, 1H), 0.38 (s, 3H).

^{11}B{^{1}H} NMR (128 MHz, $CDCl_3$) δ = 34.8.

Synthesis of arylCBS catalysts: General procedure

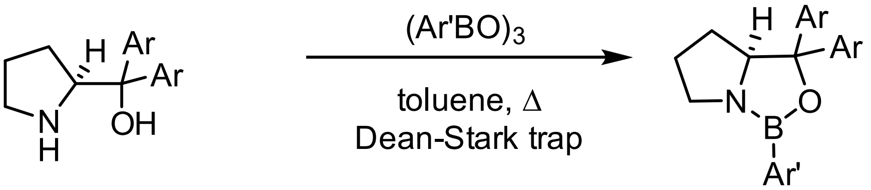

Prepared according to a literature procedure:[S12] Triarylboroxine (0.33 eq) and the respective prolinol (1.00 eq) were dissolved in dry toluene (0.4 M) and heated to 175 °C (bath temperature) for 18 h in a Dean Stark apparatus with azeotropic removal of water. Most of the toluene was removed via the Dean-Stark outlet. After cooling to room temperature remaining volatiles were removed under reduced pressure. The residue

was dried *in vacuo* at 60 °C for 1 h and then subjected to vacuum sublimation (180 °C / 3 · 10^{-2} mbar), furnishing the desired compound as a colourless powder.

(4*S*)-2-(2-methylphenyl)-5,5-diphenyl-pyrrolidino-[1,2-c][1,3,2]-oxazaborolidine (**4**)

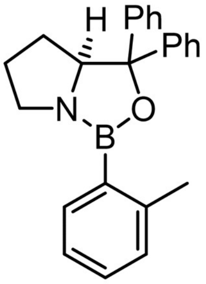

(**4**)

Yield: 39% (7.89 mmol scale)
^{1}H NMR (500 MHz, $CDCl_3$) *δ* = 7.75 (dd, *J* = 7.4, 1.6, 1H), 7.67 – 7.63 (m, 2H), 7.55 – 7.48 (m, 2H), 7.40 – 7.33 (m, 5H), 7.31 – 7.25 (m, 4H), 4.59 (dd, *J* = 9.8, 5.7, 1H), 3.48 (ddd, *J* = 10.6, 8.3, 5.9, 1H), 3.27 (ddd, *J* = 10.6, 9.1, 5.2, 1H), 2.69 (s, 3H), 1.96 – 1.73 (m, 3H).
^{11}B{^{1}H} NMR (128 MHz, $CDCl_3$) *δ* = 32.6.

(4*S*)-2-(2-methylphenyl)-5,5-bis(3,5-dimethylphenyl)-pyrrolidino-[1,2-c][1,3,2]-oxazaborolidine

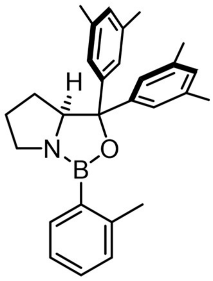

Yield: 40% (1.00 mmol scale)
^{1}H NMR (500 MHz, $CDCl_3$) *δ* = 7.68 (dd, *J* = 7.4, 1.6, 1H), 7.32 (td, *J* = 7.5, 1.6, 1H), 7.25 – 7.14 (m, 5H), 7.07 (d, *J* = 1.6, 2H), 6.87 (s, 1H), 6.85 (s, 1H), 4.49 (dd, *J* = 9.8, 5.7, 1H), 3.40 (ddd, *J* = 10.6, 8.3, 5.8, 1H), 3.19 (ddd, *J* = 10.6, 9.2, 5.2, 1H), 2.63 (s, 3H), 2.30 (s, 6H), 2.29 (s, 6H), 1.88 – 1.66 (m, 3H).
^{11}B{^{1}H} NMR (128 MHz, $CDCl_3$) *δ* = 32.5.

(4*S*)-2-(2,6-dimethylphenyl)-5,5-bis(3,5-dimethylphenyl)-pyrrolidino-[1,2-c][1,3,2]-oxazaborolidine

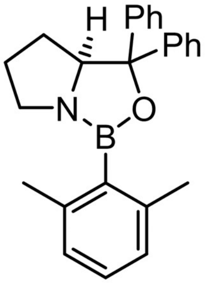

Yield: 56% (1.62 mmol scale)
^{1}H NMR (400 MHz, $CDCl_3$) *δ* = 7.25 (s, 1H), 7.14 (s, 1H), 6.99 (s, 1H), 6.87 (s, 1H), 6.85 (s, 1H), 4.43 (s, 1H), 3.29 – 3.18 (m, 1H), 3.07 – 2.96 (m, 1H), 2.31 (s, 6H), 2.29 (s, 12H), 1.91 – 1.67 (m, 1H).
^{11}B{^{1}H} NMR (128 MHz, $CDCl_3$) *δ* = 34.7.

Fluorination of **6** under CBS phase transfer catalysis

*General procedure for the fluorination of **6** with CsF under CBS phase-transfer catalysis:*

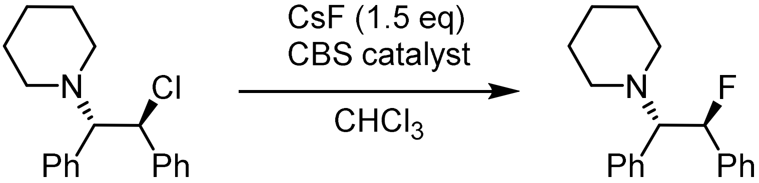

A Schlenk tube was charged with **6** (1.0 eq), the catalyst (0.1 eq) and CsF (1.5 eq, finely ground, stored in a glove box). After addition of dry $CHCl_3$ (to give a 0.5 M solution), the reaction mixture was magnetically stirred at 1000 rpm. Progress of the reaction was monitored by subjecting aliquots of the mixture to ^{1}H NMR spectroscopy. After complete conversion, the reaction mixture was filtered through Celite®, eluting with EtOAc. The eluate was evaporated to dryness and the residue adsorbed to silica. Pure **7** was isolated by column chromatography and identified by ^{1}H NMR spectroscopy.[S5] Results are summarised in Table S3.

Table S3: Results of fluorination of 6 with CsF under CBS phase-transfer catalysis

Catalyst	Time / h	Yield / %	*er* / *ee*
comm. **4** (toluene removed)	10	80	65:35 / 30%
comm. **11** (solid)	30	67	50:50 / 0%
**4** (independent synthesis)	24	84	60:40 / 20%
comm. **4** (at −20 °C)	168	65	60:40 / 20%
Combination 1	45	71	55:45 / 10%
Combination 2	36	75	55:45 / 10%

Further insight into CBS catalysts as phase-transfer reagents

Attempts were made to isolate pure CBS catalysts. This proved challenging as literature reports used the formed crude CBS catalysts without any further purification. We succeeded in the purification of several CBS derivatives by sublimation (120 – 180 °C; 10^{-2} mbar), but thermal [2+3] cycloreversion[S12] limited purity to ca. >90%, which is, however, still significantly higher than that of the commercial material (*cf.* Figure S9).

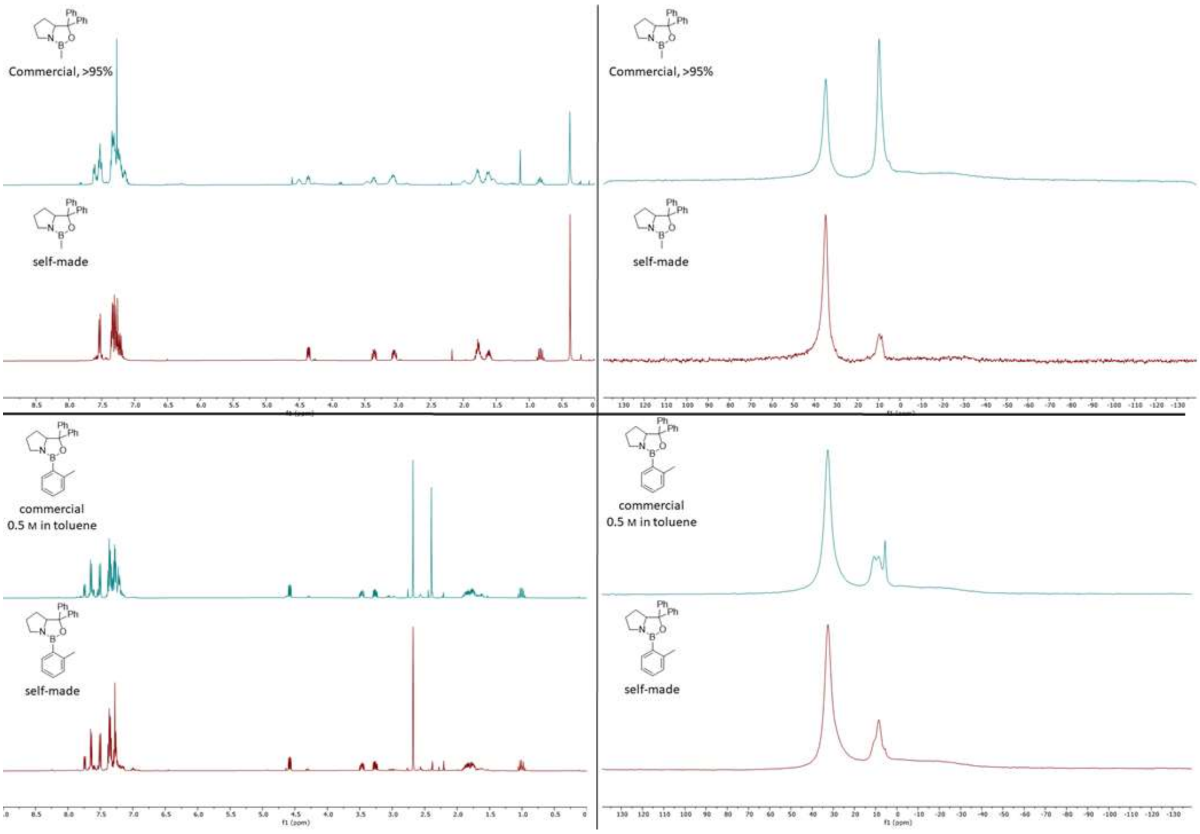

Figure S9: Comparison of the ^{1}H and $^{11}B\{^{1}H\}$ NMR spectra (400 / 128 MHz, respectively) of commercial (blue, sold as "> 95% purity") and self-made (red) CBS catalysts in dry $CDCl_3$. Note: Commercial **4** was supplied as a solution in toluene. All volatiles were removed under reduced pressure ($5 \cdot 10^{-2}$ mbar) from the sample for four hours prior to the NMR experiment.

These higher purity CBS catalysts gave worse outcomes than using commercial batches of **4** in the fluorination of **6** with CsF. In addition, all > 90% purity CBS catalysts (including independently synthesised **4**) displayed an induction period before significant fluorination occurred (Figure 6 in manuscript). This indicated that CBS catalysts are actually pre-catalysts for phase transfer fluorination. It should be noted that **1** and BEt_3 did not display induction periods during fluorination of **6** under identical conditions thus are on-cycle species.

Seeking to identify an on cycle species the impurities in commercial CBS, **12**, **13** and **14** were tested as fluorination catalysts along with other possible impurities from CBS hydrolysis and/or reaction of CBS with CsF/H_2O (see Figure S10). However, these all gave poorer outcomes in the fluorination of **6** relative to using commercial CBS or independently synthesised CBS (pre)catalysts.

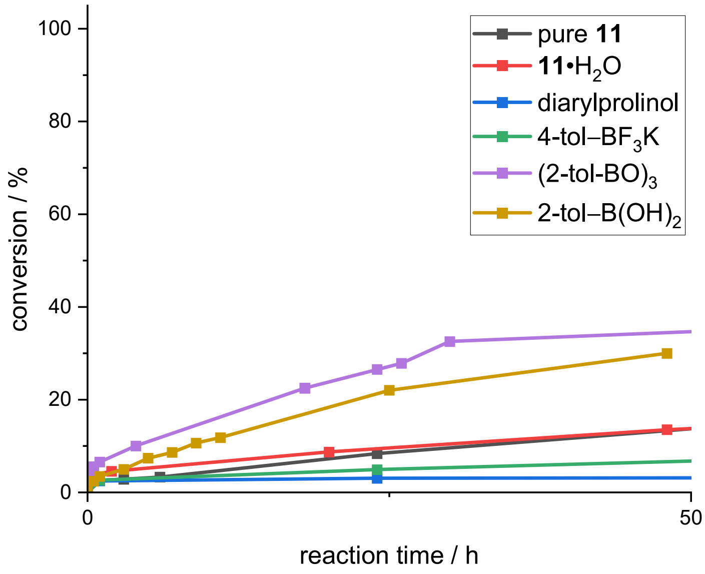

Figure S10: Plot of conversion vs. reaction time for the fluorination of **6** with CsF catalysed by various catalysts.

We hypothesised that a more complex catalyst derived from the partial hydrolysis of CBS and subsequent condensation of several boron species present in the reaction mixture maybe the true catalyst. Therefore, we combined one equivalent of **15** and **16** and found that this combination resulted in more efficient catalysis (than **15** or **16** on their own) and had no induction period, with activity now approaching that of the commercial CBS catalyst **4** (Figure S11). A comparable outcome was obtained on combining two equivalents of **15** with one equivalent of the boronic acid **17**. Thus, we propose that a multi-boron containing aggregate is an on-cycle species and this is formed from partial hydrolysis of the oxazaborolidine unit and subsequent aggregation during the induction period.[a] The aggregation process may be assisted by the formation of the β-amino-alcohol, **10**, which occurs more rapidly (than fluorination) on addition of **6**, this will remove water from the system, favouring condensation. Variable quantity of water was found to be an important factor via Karl Fischer titration of CsF (as a neat solid which dissolves completely in MeOH used as titration medium): Commercial CsF (Sigma Aldrich) was found to have a water content of 4500 ppm. Treatment of CsF under vacuum ($2{\cdot}10^{-2}$ mbar, 120 °C for 6 h) led to 1500 ppm of water still present, and only thorough drying ($2{\cdot}10^{-2}$ mbar, 200 °C, 18 h) reduced the water content to 460 ppm. However, this drier material showed reduced reactivity when employed in fluorination reactions.

[a] Usually, formation of a six-membered boroxine is thermodynamically favoured. Formation of linear boroxanes is reported to happen under special circumstances only: a) J. Walkowiak and B. Marciniec, *Tetrahedron Lett.*, 2010, **51**, 6177–6180; b) Y. Wu, L. Liu, J. Su, K. Yan, J. Zhu and Y. Zhao, *Chem. Commun.*, 2016, **52**, 1582–1585.

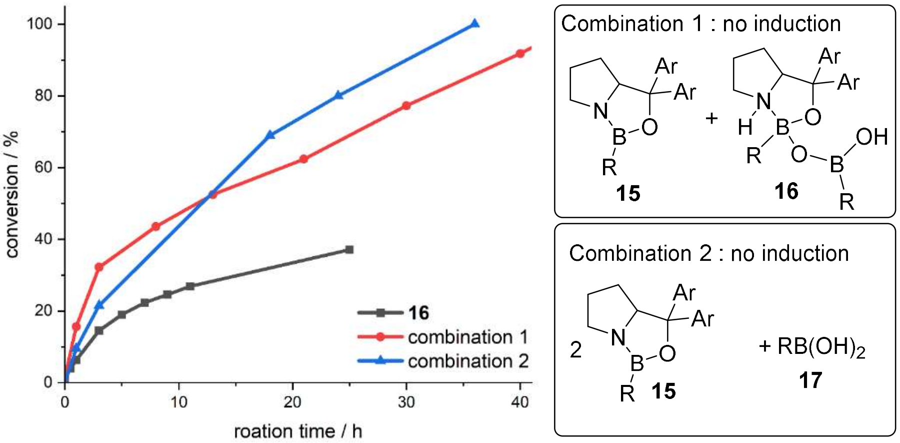

Figure S11: Plot of conversion vs. reaction time for the fluorination of **6** with CsF catalysed by various catalysts containing more than one boron atom.

MF binding studies

DOSY studies on Cs[FBEt3]

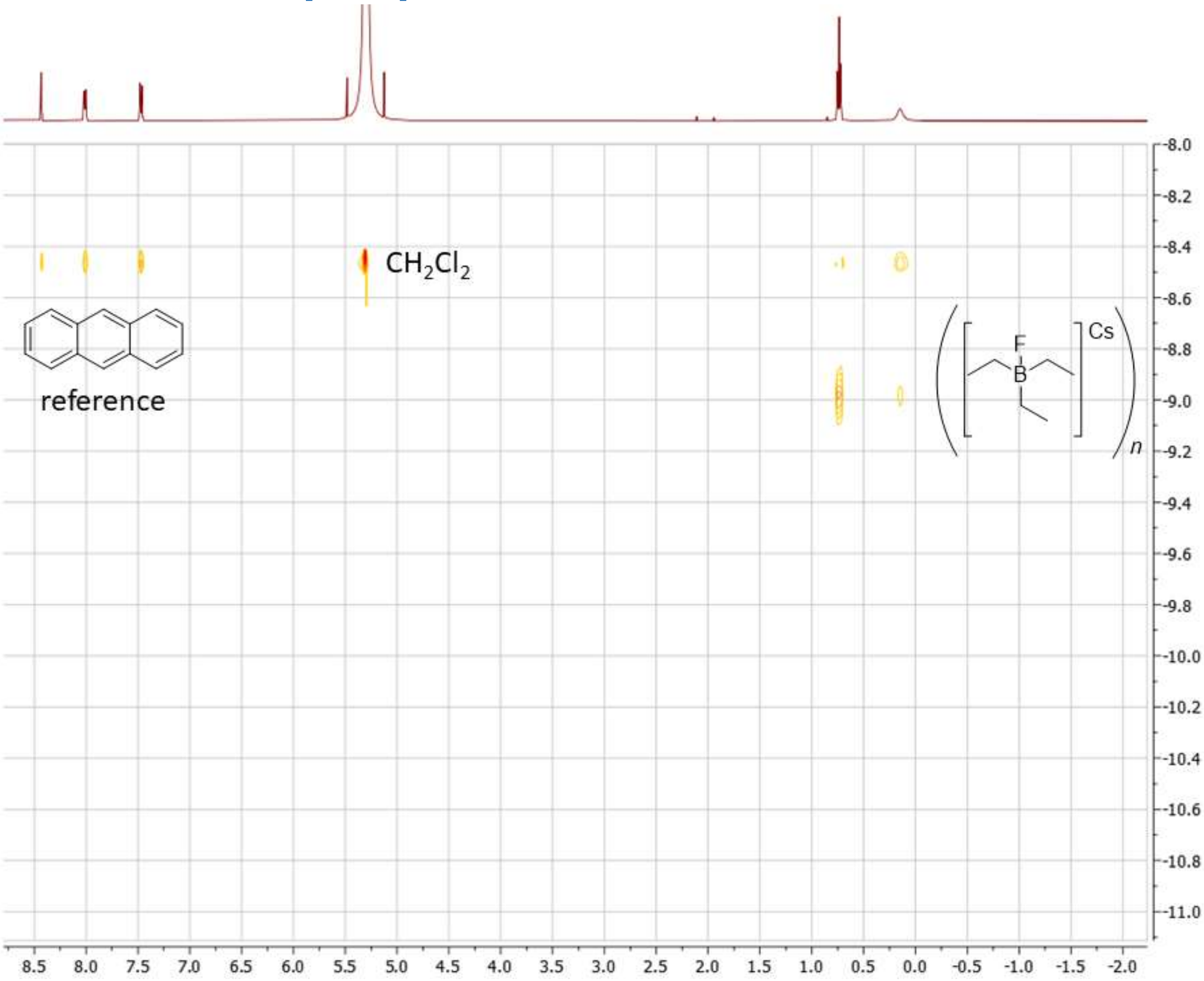

Figure S12: ^{1}H DOSY NMR spectrum (500 MHz) of $Cs[FBEt_3]$ in CH_2Cl_2.

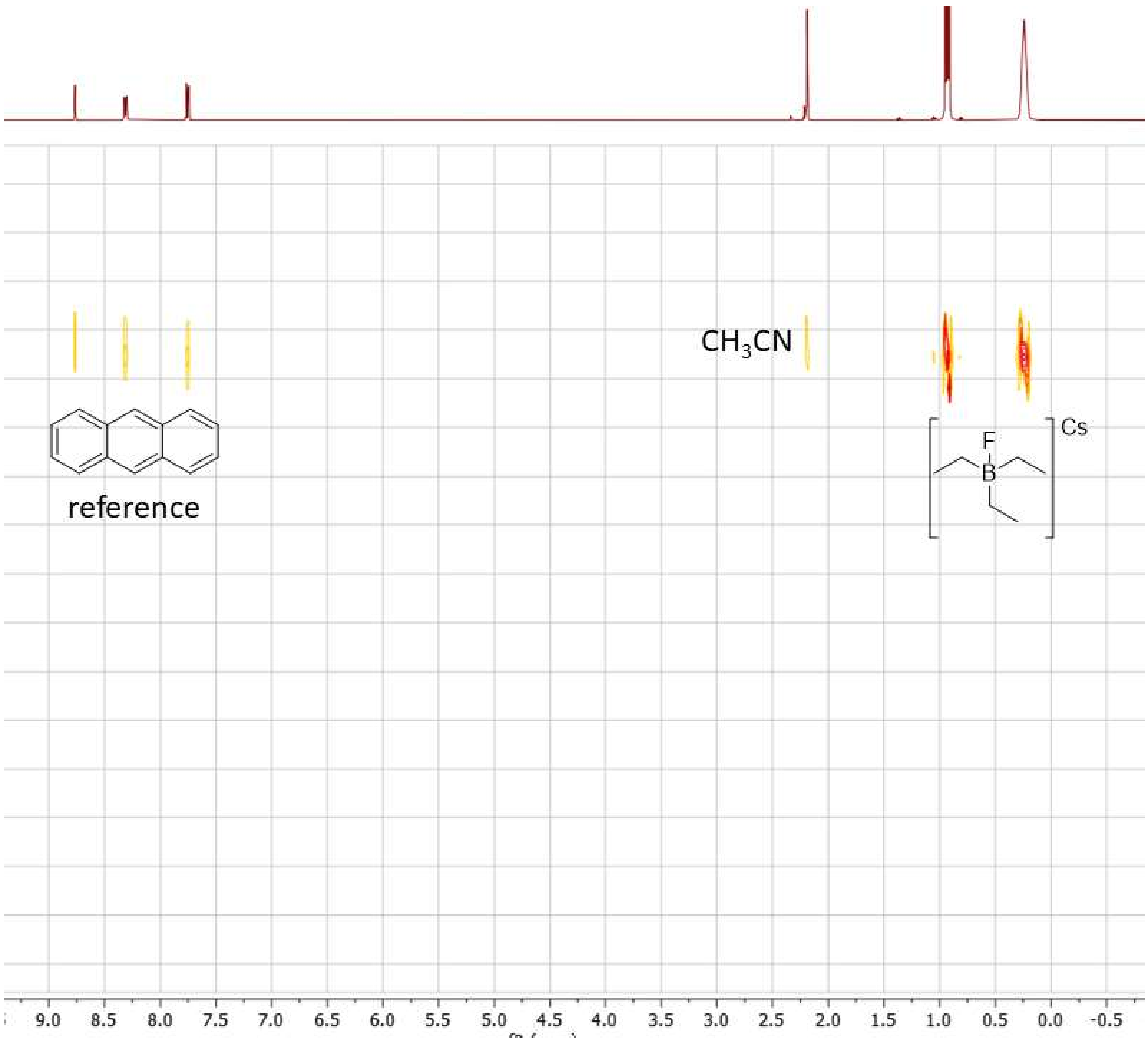

Figure S13: ^{1}H DOSY NMR spectrum (500 MHz) of $Cs[FBEt_3]$ in CH_3CN.

Complexation of **1** with F^-

Cs[**1–F**] in MeCN

An NMR tube with a J. Young's valve is charged with **1** (17 mg, 50 µmol) and CsF (11 mg, 75 µmol). After addition of dry MeCN (0.6 mL), the tube is sealed and agitated for 30 min. NMR spectra of all relevant nuclei are recorded:

^{1}H NMR (400 MHz, CH_3CN) *δ* = 7.98 (s, 2H), 7.59 (s, 1H), 1.14 (s, 6H), 0.96 (s, 6H).
^{11}B{^{1}H} NMR (128 MHz, CH_3CN) *δ* = 7.4 (d, *J* = 71.6).
^{19}F NMR (376 MHz, CH_3CN) *δ* = –62.0 (s, 6F), –130.2 (q, *J* = 71.6, 1F).

Cs·[2.2.2.]-cryptand [**1–F**] in $CDCl_3$

An NMR tube with a J. Young's valve is charged with **1** (3.4 mg, 10 µmol), [2.2.2]-cryptand (56.4 mg, 150 µmol), and CsF (22.7 mg, 150 µmol). After addition of dry $CDCl_3$ (0.6 mL), the tube is sealed and agitated for 30 min. NMR spectra of all relevant nuclei are recorded:

^{1}H NMR (400 MHz, $CDCl_3$) δ = 8.46 – 8.11 (m, 2H), 7.41 (s, 1H), 1.23 (s, 6H), 1.07 (s, 6H).
^{11}B{^{1}H} NMR (128 MHz, $CDCl_3$) δ = 2.9 (br).
^{19}F NMR (376 MHz, $CDCl_3$) δ = –62.3 (s, 6F), –144.4 (br, 1F).

Crystallographic Data

Crystallographic data for compound Cs**[5-F]** were recorded on a Bruker D8 VENTURE diffractometer, at 100 K with Mo Kα radiation (λ = 0.71073 Å). For data set **3** Bruker APEX3 software package was used for data collection, the applications SAINT[S13] and SADABS[S14] were used for the data reduction and absorption corrections of the data, respectively. All further data processing was undertaken within the Olex2 software package.[S15] The molecular structure was solved with the ShelXT[S16] structure solution program using Intrinsic Phasing and refined with the ShelXL[S17–S19] refinement package using Least Squares minimisation. Non-hydrogen atoms were refined anisotropically. Hydrogen atoms were all located in a difference map and repositioned geometrically.

Selected crystallographic data are presented in Table S4 and full details in cif format can be obtained free of charge from the Cambridge Crystallographic Data Centre via www.ccdc.cam.uk/data_request/cif.

Table S4. Select crystallographic data for compound Cs**[5-F]**.

	**Cs[5-F]**
CCDC No	2116585
Empirical formula	$C_{42}H_{71}B_2Cs_2F_2N_3$
Formula Weight	943.45
Temperature (K)	100.0
Radiation	0.71073
Crystal system	Orthorombic
Space group	$P\ 2_12_12_1$
a (Å)	11.5055(10)
b (Å)	17.6861(18)
c (Å)	24.528(2)
α(°)	90
β(°)	90
γ(°)	90
Cell volume (Å^3)	4557.4(8)
Z	4
ρ calc (gcm^{-3})	1.375
μ (mm^{-1})	1.638
F (000)	1928.0
Crystal size/ mm^3	0.168 x 0.155 x 0.064
2θ range for data collection/°	4.51 to 50.822
Index ranges	$-12 \le h \le 12$; $-21 \le k \le 21$; $-29 \le l \le 29$
Reflections collected	269071
Independent reflections	8401 [R_{int} = 0.0913; R_{sigma} = 0.0225]
Data/restraints/parameters	8401/0/469
Goodnes-of-fit-on F^2 (*GOF*)	1.067
Final *R* indices [I>2σ(I)]	R_1 = 0.0235; wR_2 = 0.0552
R indices (all data)	R_1 = 0.0255; wR_2 = 0.0560
Largest diff. peak and hole (e Å^{-3})	1.03 / –0.62
Flack parameter	–0.015(6)

Computational Details

All calculations were performed using the Gaussian09 series of programs.[S20] Geometry optimisations were completed with the DFT method using the B3LYP functional[S21] with Grimme's D3(BJ) dispersion corrections[S22] and the Def2TZVPP[S23] as a basis set. All geometry optimizations were full, with no restrictions. Stationary points located in the potential energy surface were characterized as minima (no imaginary frequencies) by vibrational analysis. Single point energy calculations were performed at the DSD-BLYP(D3BJ)[S24]/Def2TZVP[S23] level of theory. Solvation free energies in dichloromethane were computed using the universal solvation model based on solute electron density (SMD)[S25] at the M05-2X[S26]/6-31G(d) level of theory. The fluoride ion affinity (FIA) for corresponding Lewis acids (LA) was calculated using TMS-isodesmic reactions shown below according to the work by Greb.[S27]

$$\mathrm{LA} + \mathrm{Me_3SiF} \xrightarrow{\Delta H} [\mathrm{LA\text{-}F}]^- + \mathrm{Me_3Si^+} \qquad \textit{eq. 1}$$

$$\mathrm{Me_3SiF} \xrightarrow[\text{952.5 KJ/mol}]{\Delta H} \mathrm{F^-} + \mathrm{Me_3Si^+} \qquad \textit{eq. 2}$$

$$\mathrm{LA} + \mathrm{F^-} \xrightarrow{\mathit{FIA}} [\mathrm{LA\text{-}F}]^- \qquad = \text{eq. 1 - eq. 2}$$

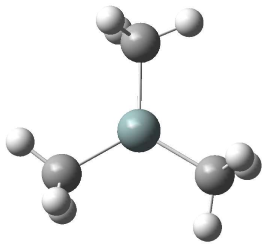

[$Me_3Si]^+$

Si	-2.05926400	1.01548500	0.00007400
C	-3.81931800	0.55995200	0.17901900
H	-4.35139900	1.35728400	0.70875900
H	-4.30939400	0.37013300	-0.77395100
H	-3.90187100	-0.33024300	0.81208300
C	-1.05904400	1.39539500	1.48072100
H	-0.18753700	0.73299200	1.51379300
H	-0.66172700	2.41304400	1.39907600
H	-1.61941600	1.30198000	2.40896400
C	-1.29994700	1.08969300	-1.65971900
H	-1.86138300	1.78937200	-2.28791300
H	-0.25261700	1.38458300	-1.63708300
H	-1.38948900	0.11100800	-2.14382400

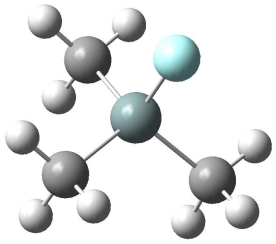

Me_3Si–F

Si	-0.00016400	-0.00010200	-0.02940700
F	0.00023000	0.00006400	-1.64909500
C	1.23456900	1.28259800	0.52536800
H	1.28045000	1.33120000	1.61541600
H	2.23578300	1.04944500	0.15944600
H	0.96490700	2.27414000	0.15819600
C	-1.72831100	0.42774000	0.52511500
H	-2.45218600	-0.30124100	0.15730800
H	-1.79313100	0.44192800	1.61515300
H	-2.02684800	1.41180300	0.16020300
C	0.49365700	-1.71030300	0.52525300
H	0.51046700	-1.77389100	1.61528800
H	-0.20709900	-2.46176500	0.15786600
H	1.48839000	-1.97097400	0.16025500

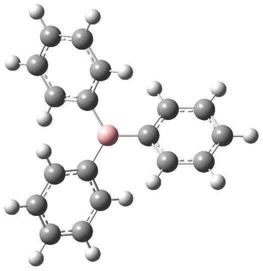

BPh_3

B	-0.13146000	0.72043400	-0.00000200
C	-0.91518500	-0.62782600	-0.00671900
C	-0.40839100	-1.76639300	-0.65478900
C	-2.15689100	-0.75421300	0.63780500
C	-1.10982800	-2.96394000	-0.67231200
H	0.54434800	-1.70410000	-1.16320100
C	-2.84901400	-1.95723600	0.65108100
H	-2.57603900	0.10314700	1.14714300
C	-2.32933100	-3.06401200	-0.01144800
H	-0.70481000	-3.82110200	-1.19393300
H	-3.79514900	-2.03210600	1.17073900
H	-2.87225700	-4.00003000	-0.01313500
C	1.42786500	0.71611000	0.01536900
C	2.14981400	-0.29255800	0.67453500
C	2.16846100	1.72057200	-0.62937500
C	3.53742500	-0.29144200	0.70313500
H	1.61046100	-1.08055500	1.18262700
C	3.55633400	1.71144000	-0.63060400
H	1.64373900	2.51158500	-1.14801700
C	4.24420800	0.70796400	0.04312900
H	4.06906800	-1.07101600	1.23272100
H	4.10281400	2.48786700	-1.14960600
H	5.32624400	0.70482800	0.05372400
C	-0.90806300	2.07276000	-0.00868400
C	-2.13669400	2.20491000	-0.67667600
C	-0.40802600	3.20917600	0.64832500
C	-2.82241900	3.41135700	-0.70374000
H	-2.55042800	1.34935600	-1.19338400
C	-1.10367600	4.41024600	0.65186200
H	0.53455900	3.14249800	1.17480100
C	-2.30985400	4.51597800	-0.03211900
H	-3.75817000	3.49059800	-1.24124600
H	-0.70435100	5.26566500	1.18068500
H	-2.84805900	5.45467400	-0.04123400

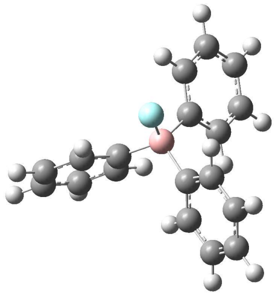

[F–BPh$_3$]$^-$

C	0.19373500	-0.63571200	-0.04881300
C	1.38817500	-0.96685600	0.60449800
C	-0.47505700	-1.68329900	-0.69232700
C	1.89337500	-2.26239600	0.60954400
H	1.92378000	-0.18124000	1.12286100
C	0.02110100	-2.98429900	-0.70131100
H	-1.41661500	-1.47935700	-1.18861400
C	1.21250500	-3.28220900	-0.04934400
H	2.82101300	-2.48027000	1.12768500
H	-0.52719500	-3.76883900	-1.21115100
H	1.60166900	-4.29349200	-0.04904000
C	-1.95012000	0.98510900	-0.04498300
C	-2.74686600	0.96392200	-1.19535100
C	-2.62708700	1.03383800	1.18076100
C	-4.13791100	0.97514700	-1.13371900
H	-2.26738300	0.95369100	-2.16688300
C	-4.01535200	1.05139300	1.25859600
H	-2.03902700	1.06481000	2.08953500
C	-4.78241900	1.01772400	0.09747400
H	-4.71993700	0.95863000	-2.04861900
H	-4.50336100	1.09104500	2.22643700
H	-5.86455300	1.03048500	0.15205200
C	0.28110400	1.69873800	-1.36638900
C	0.64912000	3.04655100	-1.26531600
C	0.42823600	1.10662300	-2.62641900
C	1.12699300	3.76639700	-2.35537800
H	0.56021700	3.53132300	-0.30107000
C	0.89875700	1.81547000	-3.72825700
H	0.18223300	0.05810000	-2.74666100
C	1.25112100	3.15440800	-3.59923600
H	1.40391500	4.80838900	-2.23692300
H	0.99852200	1.31986200	-4.68766700
H	1.62218900	3.71053100	-4.45196500
F	0.18005800	1.57134100	1.13438200
B	-0.31880300	0.91493000	-0.06574600

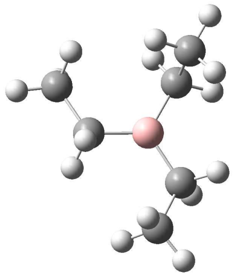

BEt_3

C	-1.07182300	3.05279700	0.09762400
H	-0.56087100	2.49825600	0.88596700
H	-1.26478700	4.05488500	0.48462500
H	-2.03915000	2.57403500	-0.06359000
C	-0.24042500	3.10360600	-1.18612100
H	-0.10937700	2.08681400	-1.58562500
H	-0.80655300	3.60504700	-1.98079300
C	1.89940300	4.08175400	0.23585900
H	2.67840700	4.83917100	0.11246400
H	1.19287800	4.46190100	0.97767900
C	2.55113300	2.79504200	0.79404900
H	3.05577100	2.98643600	1.74184100
H	1.81043600	2.01292700	0.96806100
H	3.29420100	2.39387300	0.10219500
C	2.01080100	3.98116000	-2.45799400
H	3.06233300	3.72020800	-2.28728000
H	2.03822400	5.07780300	-2.55986600
C	1.51497400	3.35789500	-3.76458500
H	0.49570500	3.67001000	-3.99672600
H	2.14263200	3.63631400	-4.61271800
H	1.51074100	2.26773900	-3.70653600
B	1.19956500	3.73485500	-1.13169700

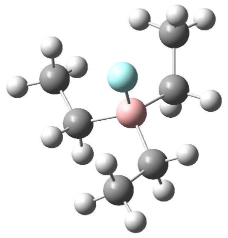

$[F–BEt_3]^-$

C	-0.89484600	2.64303100	0.01261100
H	-0.52177400	1.63044300	0.18747700
H	-0.67899300	3.22380200	0.91340200
H	-1.98786200	2.58105300	-0.07476100
C	-0.22266300	3.25680500	-1.22089300
H	-0.58883400	2.72190300	-2.10740100
H	-0.57462100	4.29250400	-1.33702400
C	2.10427900	3.95329700	0.08496400
H	3.08955100	4.35486200	-0.19428000
H	1.51157100	4.83164200	0.37989800

C	2.30411200	3.04902300	1.30861300
H	2.82536700	3.55229900	2.13394300
H	1.35419600	2.68151600	1.70216900
H	2.88721500	2.16750700	1.03370000
C	1.99968200	3.96606800	-2.59967000
H	3.09584000	3.88302800	-2.58461600
H	1.78642400	5.04440900	-2.56044500
C	1.48702200	3.40004500	-3.92843600
H	0.41084400	3.55951700	-4.04014200
H	1.97177900	3.84335000	-4.80832400
H	1.65396600	2.32018000	-3.96912200
F	1.85420700	1.81972500	-1.27989000
B	1.42318900	3.23589700	-1.24203600

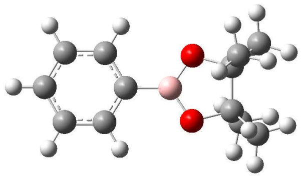

PhBPin

C	0.30262900	2.90899800	1.17820600
C	1.69102400	2.91173300	1.18175800
C	2.41391000	3.07002200	-0.00656900
C	1.70107600	3.22529000	-1.20142500
C	0.31275500	3.22204200	-1.21037500
C	-0.38794600	3.06398300	-0.01921200
H	-0.24230300	2.78583100	2.10483800
H	2.22865400	2.78946200	2.11287000
H	2.24657800	3.34994400	-2.12761600
H	-0.22438700	3.34293800	-2.14184600
H	-1.47008300	3.06165300	-0.02411400
C	6.10378500	3.35951400	-0.72421400
C	6.09923500	2.79559600	0.74072900
B	3.96206200	3.07312900	-0.00005800
O	4.71753200	2.99884200	1.14304400
O	4.72614500	3.15051900	-1.13727400
C	6.35922700	1.29273000	0.80525000
H	7.39655000	1.05657600	0.57018600
H	6.14564400	0.94285100	1.81417900
H	5.71399900	0.75157800	0.11444400
C	7.00540500	3.52931900	1.71343500
H	6.92525100	3.07469400	2.70021000
H	8.04597700	3.46312600	1.39308400
H	6.73321100	4.57789100	1.80070600
C	6.35805000	4.86344200	-0.78687900
H	7.39259500	5.10393400	-0.54409100
H	6.15057500	5.21232800	-1.79742500
H	5.70540700	5.40196600	-0.10101500
C	7.02052700	2.62958300	-1.68983000

H	6.94621700	3.08398200	-2.67716500
H	8.05827600	2.69999400	-1.36133900
H	6.75332500	1.57991300	-1.77928000

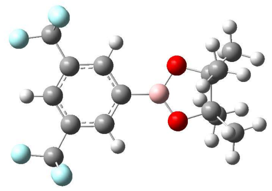

Compound 1

C	0.29691000	2.90973700	1.17155400
C	1.68631900	2.91200500	1.17855800
C	2.40607700	3.07021600	-0.00705300
C	1.69592100	3.22532100	-1.19885200
C	0.30650200	3.22144900	-1.20387800
C	-0.40125200	3.06394700	-0.01921400
H	2.22031800	2.78992000	2.11105100
H	2.23740200	3.35004500	-2.12666800
H	-1.47955400	3.06169800	-0.02383700
C	6.09647600	3.36281800	-0.72361900
C	6.09162800	2.79299300	0.74013900
B	3.96009800	3.07351200	-0.00051200
O	4.70388300	2.99115200	1.14282100
O	4.71291400	3.15889100	-1.13768400
C	6.35205100	1.29093300	0.80176300
H	7.38980500	1.05784300	0.56662800
H	6.13944100	0.93825600	1.80976600
H	5.70918200	0.74920300	0.10916800
C	6.99194700	3.52712100	1.71681800
H	6.91150700	3.06932600	2.70189000
H	8.03299000	3.46438900	1.39790700
H	6.71721400	4.57474100	1.80629000
C	6.35110900	4.86595300	-0.78326900
H	7.38598500	5.10341200	-0.53996900
H	6.14502300	5.21763800	-1.79297200
H	5.70048900	5.40503000	-0.09586500
C	7.00775700	2.63240800	-1.69287200
H	6.93356800	3.08990900	-2.67857100
H	8.04586100	2.69935300	-1.36539000
H	6.73803600	1.58368200	-1.78458200
C	-0.43295000	2.73782300	2.47563800
C	-0.41278600	3.38859300	-2.51444400
F	-0.11244200	1.56950500	3.07402300
F	-1.76974300	2.75310800	2.32597700
F	-0.11899200	3.71662500	3.35243100
F	-1.75071000	3.37936300	-2.37487300
F	-0.08306900	4.55192100	-3.11736800
F	-0.09614600	2.40319200	-3.38295300

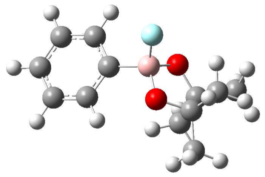

[PhBPin–F]⁻

C	-3.80310400	2.28053100	0.32071600
C	-2.43833200	2.21755600	0.59271500
C	-1.55760600	3.23457100	0.21229200
C	-2.11694100	4.33024600	-0.45743300
C	-3.47618700	4.40617800	-0.74182100
C	-4.33017500	3.37678000	-0.35319900
H	-4.45738000	1.47370400	0.63368200
H	-2.03152100	1.36224000	1.11858500
H	-1.46474200	5.14161300	-0.75889100
H	-3.87474000	5.26812300	-1.26624200
H	-5.39060200	3.43113200	-0.57093700
C	1.53982900	1.36511100	0.80450300
C	1.41429800	1.65996100	-0.73293000
O	0.37725300	1.97296800	1.32344000
O	0.83411400	2.94252200	-0.75630200
C	1.53645900	-0.11617600	1.17174700
H	2.37233600	-0.64502600	0.70587300
H	1.62769000	-0.22231800	2.25446100
H	0.60649000	-0.59015200	0.86440200
C	2.77805600	2.03761800	1.41839800
H	2.69657200	1.97705500	2.50464300
H	3.70882900	1.55380600	1.11143300
H	2.80867200	3.08947100	1.14140400
C	0.46870100	0.66943100	-1.43236700
H	0.24905200	1.05435900	-2.42901000
H	0.90583300	-0.32736100	-1.53179500
H	-0.47114900	0.59219100	-0.88889800
C	2.74495900	1.69303100	-1.48230400
H	3.27309400	0.73927800	-1.39749900
H	2.56210000	1.88874400	-2.54064900
H	3.38425200	2.48565500	-1.09951100
F	0.47187100	4.37502100	1.09163000
B	0.04545700	3.14662900	0.48818700

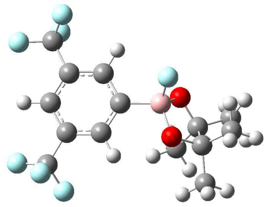

[1–F]⁻

C	-3.78295700	2.28615100	0.32805600
C	-2.41820000	2.23722300	0.60789600
C	-1.54965900	3.25254400	0.20843900

C	-2.11335200	4.33153800	-0.47691200
C	-3.47525200	4.38733900	-0.76160900
C	-4.32525400	3.36145500	-0.36318600
H	-2.00388600	1.39499700	1.14598700
H	-1.47051800	5.14419900	-0.78901200
C	1.53097600	1.37976800	0.81188700
C	1.39551000	1.65094100	-0.72916900
O	0.37773700	2.01402100	1.33118800
O	0.82115700	2.93999200	-0.76794400
C	1.50917500	-0.09394000	1.20320500
H	2.33542200	-0.63993500	0.74090600
H	1.60575200	-0.18428700	2.28653100
H	0.57223600	-0.56196300	0.90873900
C	2.77983100	2.04737600	1.40604100
H	2.70657200	2.00450400	2.49343200
H	3.70135200	1.54710300	1.09932000
H	2.82375000	3.09432600	1.11255300
C	0.43978500	0.65761800	-1.40812000
H	0.21163000	1.03124500	-2.40689400
H	0.87346000	-0.34063800	-1.50052100
H	-0.49512200	0.58301200	-0.85569700
C	2.71989000	1.66740800	-1.48823500
H	3.24380000	0.71287600	-1.39184200
H	2.53041100	1.84679200	-2.54800400
H	3.36567200	2.46277900	-1.12299600
F	0.46971800	4.41371300	1.05068800
B	0.06018800	3.17021700	0.47884900
C	-4.67293000	1.18722800	0.81781700
C	-4.01623200	5.54382900	-1.54310200
F	-4.93894300	1.28729800	2.14756400
F	-5.87888100	1.16699000	0.19882600
F	-4.13121400	-0.04008700	0.64093700
F	-5.36639700	5.64530000	-1.47125300
F	-3.71937300	5.45972800	-2.86723300
F	-3.52018600	6.73199400	-1.12805900
H	-5.37975300	3.40142100	-0.58467400

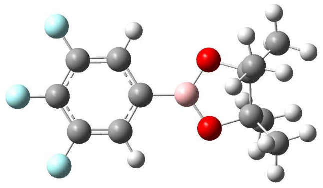

Compound 2

C	0.32173500	2.91190200	1.17007300
C	1.70169800	2.91280500	1.18651300
C	2.41358800	3.06998000	-0.00693000
C	1.71132300	3.22420200	-1.20645300
C	0.33128600	3.21922900	-1.20187400
C	-0.37997500	3.06403700	-0.01894100
H	2.21675300	2.79059900	2.12856100

H	2.23391600	3.34865700	-2.14404300
C	6.10217800	3.36065000	-0.72424700
C	6.09740000	2.79446800	0.74063500
B	3.96438500	3.07314000	-0.00047400
O	4.71181700	2.99546800	1.14309800
O	4.72075500	3.15388400	-1.13793700
C	6.35742100	1.29217000	0.80454800
H	7.39493300	1.05766000	0.56943400
H	6.14474000	0.94135200	1.81324000
H	5.71354500	0.75011400	0.11315300
C	7.00015400	3.52937500	1.71480200
H	6.92012900	3.07384600	2.70103300
H	8.04089700	3.46480700	1.39511600
H	6.72663000	4.57751900	1.80234600
C	6.35641700	4.86402400	-0.78618900
H	7.39106700	5.10290300	-0.54301200
H	6.15016900	5.21386900	-1.79655300
H	5.70486400	5.40340700	-0.09991800
C	7.01579500	2.62949400	-1.69106800
H	6.94192800	3.08473900	-2.67790900
H	8.05361800	2.69828900	-1.36291900
H	6.74727400	1.58024600	-1.78082400
F	-0.37812000	2.76259700	2.30519700
F	-1.71383100	3.06120600	-0.02468700
F	-0.35942500	3.36562500	-2.34296500

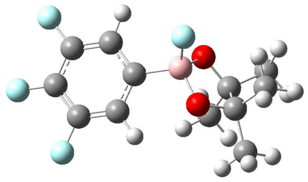

[2–F]⁻

C	-3.77083200	2.28638900	0.33372400
C	-2.41872500	2.21651000	0.60811000
C	-1.55398700	3.23905100	0.20978800
C	-2.10521500	4.33190100	-0.46949700
C	-3.45670000	4.38465000	-0.74004300
C	-4.31184800	3.36566700	-0.34508200
H	-2.02606200	1.36181300	1.14205000
H	-1.47593900	5.15180100	-0.78877900
C	1.53399400	1.36506200	0.80279800
C	1.41330000	1.66329800	-0.73438400
O	0.37242900	1.98165100	1.32112400
O	0.83379000	2.94893100	-0.75629100
C	1.51900100	-0.11590500	1.16796100
H	2.35274200	-0.64883400	0.70355600
H	1.60687700	-0.22437800	2.25048200
H	0.58747600	-0.58455500	0.85742000
C	2.77298500	2.02941800	1.42193800
H	2.68819000	1.96695600	2.50762000

H	3.70109500	1.54036300	1.11655800
H	2.81257200	3.08162600	1.14734600
C	0.46891300	0.67756800	-1.44050800
H	0.25350600	1.06569400	-2.43669900
H	0.90543700	-0.31883400	-1.54180500
H	-0.47309600	0.59661000	-0.90130100
C	2.74580900	1.69824100	-1.47913100
H	3.27238700	0.74386100	-1.39515900
H	2.56667900	1.89689400	-2.53734700
H	3.38468100	2.48895400	-1.09211400
F	0.46421600	4.38507200	1.08663700
B	0.05470600	3.15344100	0.48661600
F	-5.63474900	3.42750700	-0.61345100
F	-4.61258800	1.29451300	0.72380700
F	-3.99523300	5.44045000	-1.40279500

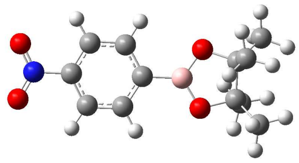

Compound 3p

C	0.27715900	2.91272900	1.18863800
C	1.66315300	2.91626000	1.18375600
C	2.38259900	3.06982400	-0.00716500
C	1.67283300	3.22043300	-1.20426000
C	0.28693500	3.21814000	-1.22110400
C	-0.38830700	3.06401200	-0.01913300
H	-0.28997500	2.79563600	2.09851900
H	2.20017700	2.79789800	2.11477300
H	2.21734800	3.34112200	-2.13061300
H	-0.27284600	3.33287700	-2.13582700
C	6.07516600	3.36140200	-0.72401100
C	6.07037000	2.79356600	0.74017700
B	3.93712100	3.07301100	-0.00064700
O	4.68371800	2.99208400	1.14243100
O	4.69270100	3.15701400	-1.13758000
C	6.33171600	1.29151300	0.80299200
H	7.36953700	1.05842200	0.56794700
H	6.11930100	0.93963500	1.81134500
H	5.68874000	0.74916600	0.11099700
C	6.97154900	3.52854400	1.71566200
H	6.89154000	3.07185400	2.70133100
H	8.01251900	3.46543900	1.39649600
H	6.69694300	4.57631400	1.80413100
C	6.33061100	4.86455600	-0.78483800
H	7.36554400	5.10210100	-0.54167000
H	6.12465300	5.21543900	-1.79487600
H	5.67986900	5.40418900	-0.09800300

C	6.98732700	2.63023400	-1.69210800
H	6.91349200	3.08663300	-2.67839200
H	8.02536300	2.69764300	-1.36441700
H	6.71781400	1.58133600	-1.78281100
O	-2.42742200	3.19900200	-1.10021000
O	-2.43613700	2.92074900	1.04428000
N	-1.86388600	3.06094700	-0.02551800

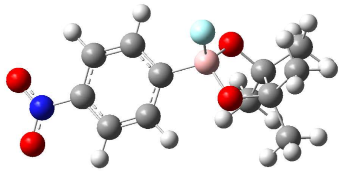

[3p–F]⁻

C	-3.82958200	2.28212300	0.37232200
C	-2.47264700	2.23203000	0.64930100
C	-1.58513000	3.22749700	0.21994200
C	-2.13066800	4.29411900	-0.51156300
C	-3.47956600	4.37080100	-0.80950500
C	-4.32400900	3.35610800	-0.36186600
H	-4.50923100	1.51402600	0.70860500
H	-2.07055500	1.40301600	1.21680300
H	-1.47012500	5.08138400	-0.85163900
H	-3.89324100	5.19156500	-1.37521500
C	1.50878900	1.36448000	0.79927200
C	1.38157600	1.68175700	-0.73309100
O	0.34408000	1.96709800	1.32840400
O	0.79647300	2.96652100	-0.73584000
C	1.50343000	-0.12105300	1.14540700
H	2.33868800	-0.64314700	0.67170400
H	1.59566500	-0.24261300	2.22610000
H	0.57359600	-0.59120800	0.83207000
C	2.74535700	2.02821900	1.42338800
H	2.66405300	1.95154600	2.50835000
H	3.67518400	1.54819600	1.10936300
H	2.77892000	3.08402300	1.16213300
C	0.43730000	0.70262900	-1.44757200
H	0.21556500	1.10228900	-2.43769500
H	0.87668300	-0.29075700	-1.56350600
H	-0.50148900	0.61090800	-0.90417600
C	2.71062600	1.73251500	-1.48237600
H	3.24088200	0.77918000	-1.41286700
H	2.52685700	1.94436800	-2.53714100
H	3.34795000	2.52030700	-1.08705100
F	0.41217700	4.37327300	1.13121400
B	0.01886700	3.14700000	0.51055300
O	-6.16353500	4.38106700	-1.31063000
O	-6.47406800	2.51745800	-0.26285500
N	-5.74473100	3.42241200	-0.66439800

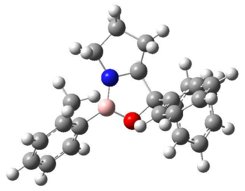

Compound 4

C	-2.88958600	2.52856600	-0.41391500
C	-4.09322100	3.51941200	-0.43302400
C	-4.20282900	4.73720600	0.47642000
C	-5.72867300	4.92474400	0.54365800
C	-6.29638600	3.48522300	0.53592600
H	-3.80039200	4.50616400	1.46175000
H	-3.68609800	5.61650500	0.09435800
H	-6.07479000	5.46628900	-0.33777500
H	-7.19513200	3.41257800	-0.08037700
H	-4.25176000	3.83499000	-1.47124100
C	-2.11547600	2.58194300	0.90469500
C	-1.21003200	3.61523500	1.14865600
C	-2.32104700	1.62953300	1.89838200
C	-0.54033000	3.70368700	2.36020900
H	-1.01849500	4.35389400	0.38332200
C	-1.64887100	1.71559500	3.11383800
H	-2.98965000	0.80300200	1.71567000
C	-0.75930400	2.75337800	3.35205900
H	0.15881700	4.51214800	2.52756300
H	-1.82050700	0.96255200	3.87150700
H	-0.23423900	2.81826300	4.29542500
C	-1.94491300	2.70290300	-1.58729200
C	-1.24657200	1.59778500	-2.07048700
C	-1.71753400	3.94770800	-2.16774900
C	-0.34486300	1.73584100	-3.11610500
H	-1.42530700	0.62988300	-1.62619200
C	-0.80912400	4.08924100	-3.21144400
H	-2.24873000	4.81954900	-1.81115500
C	-0.12086100	2.98291800	-3.69015300
H	0.18457500	0.86737100	-3.48472700
H	-0.64541500	5.06389300	-3.65145900
H	0.58187600	3.08960900	-4.50541200
O	-3.52881800	1.23659400	-0.52381600
H	-6.04623000	5.48841400	1.41917800
H	-6.56105400	3.16024200	1.54169300
N	-5.20759000	2.68058500	0.00762500
B	-4.85778200	1.33014100	-0.12039800
C	-5.71336400	0.03862700	0.06713000
C	-5.78510500	-0.84805500	-1.01622400
C	-6.39510700	-0.28985200	1.25034400
C	-6.53324600	-2.01408600	-0.95505300
H	-5.23998700	-0.61450600	-1.92213000
C	-7.13542700	-1.47211300	1.30553600

C	-7.21537500	-2.32547400	0.21511600
H	-6.57769100	-2.67855500	-1.80759500
H	-7.64955800	-1.72973400	2.22370600
H	-7.79740800	-3.23520100	0.28248100
C	-6.33634400	0.58800200	2.47455400
H	-6.31044400	-0.01416200	3.38309600
H	-5.45801300	1.23139900	2.47340400
H	-7.21764800	1.23058500	2.54165400

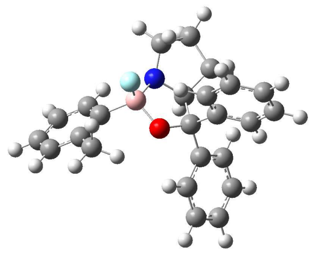

[4–F]$^{-}$

C	0.86553700	0.23234400	0.06615700
C	0.49754500	-0.38055300	1.48808800
C	1.56083300	-1.16324400	2.27906500
C	0.77817400	-2.36861900	2.81818700
C	-0.22038500	-2.64240600	1.69062500
H	2.35557900	-1.51063200	1.62047900
H	2.02497300	-0.56576900	3.06595400
H	0.23980100	-2.09499700	3.72913700
H	-1.10075000	-3.18965400	2.04216200
H	0.13957300	0.46690800	2.08753400
C	2.08326500	-0.45571000	-0.57100300
C	3.39116300	0.00934200	-0.42383400
C	1.88313200	-1.62970700	-1.30056000
C	4.46709500	-0.67812500	-0.97482800
H	3.58022900	0.92297900	0.11986000
C	2.95810700	-2.32114400	-1.84551700
H	0.87537600	-1.99168200	-1.43747800
C	4.25771400	-1.85240100	-1.68693800
H	5.47038000	-0.28919600	-0.84948400
H	2.77509900	-3.23207800	-2.40227200
H	5.09345100	-2.38971600	-2.11765900
C	1.09820100	1.73431900	0.16729900
C	0.58242300	2.58410900	-0.80842300
C	1.81780600	2.29792800	1.22141200
C	0.79518500	3.95560700	-0.74363000
H	-0.00435700	2.14733100	-1.60219200
C	2.03438400	3.66973300	1.29035800
H	2.20913000	1.65984900	2.00233600
C	1.52606200	4.50622400	0.30388500
H	0.38136900	4.59858200	-1.51057900
H	2.59324700	4.08471400	2.12011100
H	1.68753700	5.57551700	0.35710400

O	-0.27375700	-0.00686600	-0.71557500
H	1.41789200	-3.22425700	3.04537200
H	0.24656900	-3.26367500	0.91099400
N	-0.57745800	-1.31558200	1.22984400
C	-2.71699500	-0.42845800	-0.09298700
C	-3.41431600	-0.04000000	-1.25611800
C	-3.36960100	-0.26282800	1.12950100
C	-4.70175000	0.48603400	-1.15689200
C	-4.65743900	0.25979500	1.22868900
H	-2.83242500	-0.55431800	2.02469000
C	-5.33035300	0.63874600	0.07604600
H	-5.22235500	0.78497900	-2.06118800
H	-5.12873500	0.37327700	2.19868600
H	-6.33133900	1.05106200	0.12970800
C	-2.77998400	-0.18992200	-2.61596100
H	-1.79163600	0.26868500	-2.63231900
H	-2.63055000	-1.24318500	-2.85858100
H	-3.39931600	0.26469000	-3.39207100
F	-1.20611200	-2.22428400	-0.94600000
B	-1.19926000	-1.02665000	-0.13126600

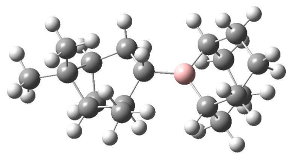

Compound 5

C	3.55807600	13.04920300	3.08858800
H	4.52803700	13.13189400	3.58901500
C	3.44326200	11.61378500	2.48758400
H	2.98295400	11.74171500	1.49464400
C	2.52582800	13.28852300	4.19447600
H	2.56564700	14.32305000	4.54258200
C	2.70287000	12.16147300	5.24253900
H	3.70840900	11.75092500	5.34609300
H	2.32300000	12.41215900	6.22861800
C	2.49134600	10.64988400	3.27458200
H	1.80430300	10.16779900	2.57704900
H	3.06194900	9.84250500	3.73814100
C	1.70858900	11.34942700	4.38142800
H	1.03582500	10.65310800	4.88624500
C	1.10024800	12.70390600	3.90476500
C	0.47191600	12.77817400	2.51765500
H	0.23928100	13.81200000	2.25419400
H	-0.46956400	12.22345700	2.51147400
H	1.09735300	12.36821400	1.73159500
C	3.53700200	14.12439000	2.00352600
H	4.31627300	13.94528100	1.26083200
H	3.70947400	15.11333300	2.43267800
H	2.58205700	14.14980400	1.47962700

B	4.79693600	10.94737600	2.05986400
C	0.07607400	13.25057800	4.90311600
H	0.43060000	13.23174600	5.93172800
H	-0.84658800	12.66764700	4.85924900
H	-0.17433300	14.28532700	4.65886500
C	4.93254700	9.41783200	1.72068500
H	3.97623200	8.88960400	1.74204600
C	5.80992700	8.80767300	2.84875500
H	5.22068600	8.82000700	3.77148500
H	6.00422300	7.75240400	2.63270500
C	6.18923100	11.66778800	1.95748300
H	6.13609200	12.74316500	2.13707300
C	7.13909800	9.53304200	3.11125400
H	7.87653100	9.23182400	2.37081600
H	7.53794700	9.20073000	4.07294100
C	7.01947500	11.06318500	3.12497900
H	8.01849800	11.50990000	3.13121900
H	6.54905100	11.37024500	4.06571000
C	6.82350600	10.01969200	0.04098900
H	7.65653100	9.47823000	0.48436400
H	7.02206600	10.02978500	-1.03363000
C	5.51804600	9.25665200	0.29894000
H	5.66840100	8.19514800	0.07732900
H	4.76262700	9.60599000	-0.41323800
C	6.80895600	11.46631800	0.55644000
H	6.23400800	12.07847700	-0.14617700
H	7.82689800	11.86882300	0.53908000

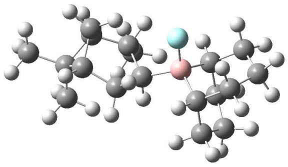

[5–F]⁻

F	5.26019000	10.75012600	4.45268600
C	3.60290100	13.00654700	3.35376900
H	4.44696400	13.07563400	4.04743800
C	3.60987000	11.56136900	2.75505900
H	3.44809400	11.67630200	1.67973600
C	2.36516200	13.28573500	4.21550900
H	2.36128300	14.32121300	4.57298800
C	2.26804000	12.15367500	5.26958000
H	3.21225900	11.69976500	5.56495100
H	1.69004800	12.42117100	6.15299600
C	2.44736500	10.66553000	3.28952800
H	1.91290200	10.21094000	2.44916900
H	2.86049700	9.83624100	3.86727000
C	1.45439100	11.38673400	4.19996800
H	0.65490600	10.71857900	4.53751400

C	1.01751200	12.76082000	3.61179800
C	0.73205500	12.84407600	2.11711300
H	0.58734700	13.88154600	1.80326300
H	-0.19075400	12.30216600	1.88590700
H	1.52899200	12.41786000	1.51704900
C	3.82110000	14.09317200	2.29818900
H	4.76721000	13.94148800	1.78068200
H	3.84858200	15.08524900	2.76117300
H	3.03087900	14.09928400	1.54665100
B	5.06590000	10.81725200	2.98718200
C	-0.18476200	13.34950600	4.35368400
H	-0.06601500	13.32279200	5.43603700
H	-1.09620800	12.79764000	4.10468400
H	-0.33993900	14.39277500	4.06283400
C	5.14764100	9.29613400	2.37811800
H	4.32579600	8.67792600	2.76237200
C	6.44776800	8.63030300	2.86618800
H	6.36041100	8.51974700	3.95011000
H	6.55146700	7.61414800	2.45553500
C	6.34967100	11.58722300	2.31595500
H	6.39439400	12.63416100	2.64582400
C	7.74104700	9.41032400	2.56697600
H	8.04131800	9.23480500	1.53303700
H	8.55370300	8.99792500	3.17701900
C	7.64387400	10.92491300	2.82156800
H	8.53271200	11.40819400	2.38795000
H	7.69122600	11.09531400	3.90009200
C	6.00163400	10.23595900	0.11643000
H	6.95644400	9.71775000	0.02688200
H	5.65990100	10.39221200	-0.91380000
C	4.99969200	9.32499800	0.84733700
H	5.07970500	8.31327500	0.42214900
H	3.98596800	9.66741000	0.61320200
C	6.22534700	11.60638300	0.78151800
H	5.37971400	12.24849800	0.51952200
H	7.10919800	12.07291200	0.32081200

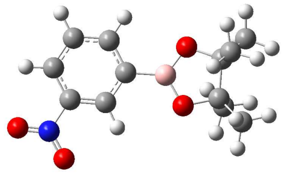

Compound 3m

C	0.13623500	2.93126300	0.96534700
C	1.52612200	2.93551500	0.96617200
C	2.25323400	3.09514800	-0.21919400
C	1.55040200	3.25190100	-1.41571900
C	0.16556000	3.24401600	-1.39887500
C	-0.55862300	3.08595000	-0.22525500
H	-0.40898400	2.80683300	1.89057400
H	2.06328200	2.81284900	1.89715000
H	2.07188600	3.37941200	-2.35168100
C	5.94308100	3.38590700	-0.93761400
C	5.93945000	2.82195700	0.52832200
B	3.80611000	3.09870100	-0.21258800
O	4.55285800	3.02124600	0.93137000
O	4.56096000	3.17918200	-1.34949200
C	6.20120500	1.32020300	0.59503900
H	7.23870800	1.08665000	0.35912000
H	5.99032400	0.97097800	1.60470500
H	5.55748200	0.77598500	-0.09476500
C	6.84115000	3.55973700	1.50123000
H	6.76181800	3.10600400	2.48837600
H	7.88191300	3.49568600	1.18158600
H	6.56670600	4.60781600	1.58650600
C	6.19719800	4.88904600	-1.00320300
H	7.23221900	5.12831900	-0.76203200
H	5.98934400	5.23646700	-2.01399900
H	5.54663700	5.43019300	-0.31734200
C	6.85484400	2.65277200	-1.90467400
H	6.77857400	3.10605200	-2.89214200
H	7.89332200	2.72231900	-1.57873300
H	6.58612200	1.60340400	-1.99214100
O	0.08677900	3.55291000	-3.68828300
O	-1.78882200	3.39649600	-2.62337800
H	-1.63661300	3.08658700	-0.26056800
N	-0.56787500	3.41054100	-2.66784100

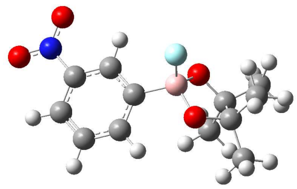

[**3m–F**]$^{-}$

C	-3.89086100	2.41822500	0.28311500

C	-2.52288300	2.37086000	0.55403400
C	-1.63949300	3.36350900	0.12269700
C	-2.18778200	4.42657700	-0.60098700
C	-3.54952500	4.46549700	-0.87454600
C	-4.42269100	3.47016200	-0.44189700
H	-4.54458400	1.63008400	0.63696700
H	-2.11268100	1.54280300	1.11831000
H	-1.55508000	5.22713800	-0.95306700
C	1.44364200	1.48742400	0.71431600
C	1.32873800	1.80345000	-0.81944000
O	0.27963100	2.09787100	1.23468200
O	0.75348400	3.09125400	-0.82778500
C	1.42757800	0.00228600	1.06263800
H	2.26256500	-0.52537200	0.59458000
H	1.51274600	-0.11842800	2.14410000
H	0.49691800	-0.46311500	0.74432500
C	2.68024600	2.14458800	1.34538600
H	2.59120200	2.07086200	2.43000700
H	3.60921000	1.65817900	1.03859400
H	2.72166300	3.19952100	1.08187900
C	0.38233400	0.82900200	-1.53851400
H	0.16894200	1.22959500	-2.53009400
H	0.81606400	-0.16739300	-1.65112400
H	-0.56030000	0.74389000	-1.00064200
C	2.66300900	1.84242000	-1.56027400
H	3.18603000	0.88531600	-1.48591800
H	2.48753900	2.05425800	-2.61644200
H	3.30333900	2.62640000	-1.16227700
F	0.36567300	4.50343200	1.03214900
B	-0.03367900	3.28027600	0.41133700
H	-5.47327300	3.53810500	-0.67430000
N	-4.10156800	5.58627000	-1.64171900
O	-5.31028700	5.59748900	-1.86902600
O	-3.34057100	6.46541000	-2.02520700

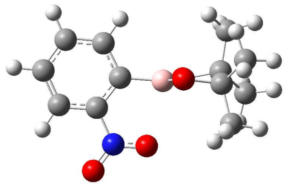

Compound 3o

C	0.31058400	1.59153700	-0.01697600
C	1.68669600	1.77788200	0.03038100
C	2.28115900	2.93454300	-0.47802600
C	1.42093200	3.87969100	-1.02610000
C	0.04339300	3.72423800	-1.08894800
C	-0.51301000	2.56349900	-0.57698500
H	-0.12240800	0.68522600	0.38440200
H	2.31150900	1.01170400	0.46963600
C	6.03142000	2.95818100	-1.01338300

C	5.90544000	3.22243800	0.53250600
B	3.84260800	3.10853500	-0.42265900
O	4.49915200	3.56987200	0.68268800
O	4.66981000	2.58413400	-1.37510200
C	6.13187700	1.97392500	1.38024600
H	7.17825700	1.67042700	1.37510600
H	5.84026400	2.18991000	2.40704000
H	5.52803600	1.14034900	1.02342100
C	6.74697200	4.37363400	1.05394400
H	6.58101500	4.49025000	2.12429500
H	7.80785200	4.17708200	0.89368000
H	6.48675700	5.30897300	0.56641300
C	6.38111200	4.20733500	-1.81673600
H	7.41428900	4.50765600	-1.64447100
H	6.25934700	3.98599200	-2.87608800
H	5.72614400	5.03939200	-1.57079300
C	6.95850000	1.81637600	-1.39262200
H	6.96811300	1.70178000	-2.47579700
H	7.97759400	2.02565700	-1.06493100
H	6.63781300	0.87441200	-0.95535700
H	-1.58342200	2.41814200	-0.61305000
H	-0.56188800	4.50076300	-1.52927700
N	2.00791900	5.11546300	-1.56623700
O	3.22461800	5.24136400	-1.47564200
O	1.26939400	5.94345300	-2.06921100

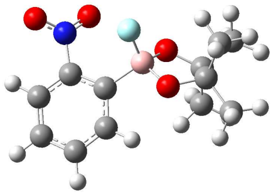

[**3o–F**]$^-$

C	-3.80840700	2.43268000	0.16321000
C	-2.42895600	2.38259800	0.31216800
C	-1.58118500	3.44013000	-0.04869500
C	-2.24426900	4.56148500	-0.54547600
C	-3.62527000	4.66272300	-0.68126300
C	-4.41697700	3.57869200	-0.34049900
H	-4.41433800	1.58126700	0.45068700
H	-1.96386000	1.50636400	0.74276800
C	1.44279200	1.48767300	0.69458700
C	1.39514600	1.70643000	-0.85926300
O	0.27519000	2.15685300	1.12614200
O	0.82761200	2.99102600	-0.97873300
C	1.37598200	0.02900200	1.13593800
H	2.21501700	-0.54495200	0.73474500
H	1.41657600	-0.02468000	2.22541200
H	0.44738500	-0.43550300	0.81025600
C	2.66946100	2.15549600	1.33430800

H	2.53525200	2.15175900	2.41681700
H	3.59738600	1.63001100	1.09646800
H	2.74770300	3.19084200	1.00923800
C	0.47659200	0.69227600	-1.55958900
H	0.31109900	1.03367400	-2.58193700
H	0.90844300	-0.31094000	-1.59183900
H	-0.49056400	0.64356200	-1.06238400
C	2.75971200	1.69726500	-1.54482800
H	3.27438600	0.74338100	-1.39988300
H	2.62714500	1.85417200	-2.61663800
H	3.38811800	2.49937900	-1.16482000
F	0.48211200	4.52895500	0.80463000
B	0.04077100	3.30348000	0.22440200
H	-5.49177600	3.63235200	-0.45735000
H	-4.05663300	5.58066600	-1.05332100
N	-1.49352900	5.75737800	-0.98537200
O	-1.92007600	6.85262500	-0.62158700
O	-0.54063600	5.60074700	-1.72103500

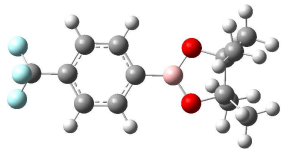

Compound 15p

C	0.30403600	2.89239300	1.17763100
C	1.69056600	2.90151100	1.17943500
C	2.41332700	3.05908900	-0.00794000
C	1.70201700	3.20667200	-1.20314300
C	0.31530300	3.19773100	-1.21694800
C	-0.38258400	3.03873200	-0.02393700
H	-0.24385200	2.76505300	2.10012600
H	2.22531400	2.78237600	2.11197900
H	2.24550900	3.32820500	-2.13029800
H	-0.22391600	3.30732700	-2.14661600
C	6.10383900	3.36122700	-0.72404400
C	6.10064800	2.79682500	0.74127100
B	3.96536500	3.06719300	-0.00055600
O	4.71494300	2.99283200	1.14288000
O	4.72394000	3.14977000	-1.13750600
C	6.36658400	1.29549100	0.80702200
H	7.40502600	1.06446800	0.57240400
H	6.15485800	0.94513000	1.81609700
H	5.72468500	0.75034400	0.11624000
C	7.00077200	3.53579300	1.71505200
H	6.92159100	3.08118900	2.70181100
H	8.04197600	3.47429000	1.39614500
H	6.72372800	4.58313300	1.80124800
C	6.35353900	4.86542300	-0.78778900
H	7.38736200	5.10811600	-0.54470800
H	6.14625300	5.21328300	-1.79865400

H	5.69987200	5.40345300	-0.10249200
C	7.02032900	2.63225500	-1.69006500
H	6.94500000	3.08635400	-2.67735800
H	8.05796100	2.70463500	-1.36198700
H	6.75516700	1.58206900	-1.77897000
C	-1.88495800	3.07818500	-0.02396700
F	-2.35050000	4.33458400	0.17440900
F	-2.41607200	2.31298700	0.95142200
F	-2.40833300	2.65339600	-1.19173600

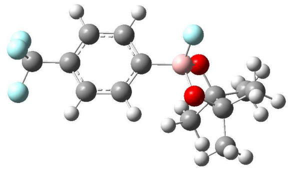

[**15p–F**]$^{-}$

C	-3.79685400	2.27323400	0.31819900
C	-2.43666200	2.21883000	0.59534600
C	-1.55574900	3.23177300	0.20463300
C	-2.11183500	4.31805500	-0.48317500
C	-3.46541000	4.39177400	-0.77607700
C	-4.31740600	3.36135300	-0.37786700
H	-4.45354600	1.47254100	0.63217600
H	-2.03159800	1.37019500	1.13168200
H	-1.45971300	5.12418200	-0.79555900
H	-3.86515200	5.23961000	-1.31681200
C	1.53841500	1.36708800	0.80692900
C	1.41383500	1.65812500	-0.73125100
O	0.37396900	1.97796400	1.32369900
O	0.82890100	2.94086900	-0.75789300
C	1.53303400	-0.11268200	1.17784700
H	2.36938000	-0.64263000	0.71462000
H	1.62268600	-0.21629400	2.26075100
H	0.60378100	-0.58767000	0.87007400
C	2.77499300	2.04096400	1.42103800
H	2.69218900	1.98301600	2.50716300
H	3.70543000	1.55567600	1.11645200
H	2.80805300	3.09212500	1.14169400
C	0.47190500	0.66503500	-1.43056000
H	0.25125400	1.04813200	-2.42753200
H	0.91267100	-0.32985000	-1.52923000
H	-0.46781900	0.58236200	-0.88752500
C	2.74478500	1.69496800	-1.47883000
H	3.27553900	0.74319200	-1.39123400
H	2.56286200	1.88776100	-2.53769400
H	3.38108800	2.49018200	-1.09675300
F	0.45975500	4.38027800	1.08505900
B	0.04889200	3.14707900	0.48621700
C	-5.78103300	3.45789800	-0.63787000
F	-6.45723300	4.10664100	0.35582700

F	-6.38249100	2.24733300	-0.75096100
F	-6.07484400	4.13795300	-1.77473700

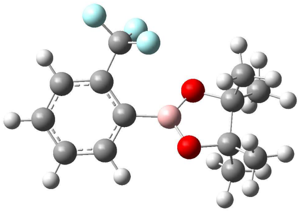

Compound 15o

C	0.42714100	2.29404300	1.02231900
C	1.80683100	2.43567800	0.95340300
C	2.43549700	3.00641700	-0.15835700
C	1.61404400	3.46479900	-1.20191100
C	0.23214100	3.33186200	-1.13436000
C	-0.36202400	2.73963100	-0.02720000
H	2.42082600	2.09829200	1.77710000
H	-1.43789800	2.63890000	0.01439600
C	6.17684700	3.16081000	-0.75944800
C	6.07873700	2.93443200	0.79062600
B	3.99697400	3.05266000	-0.12609000
O	4.67497900	3.21975100	1.05297100
O	4.82055300	2.86310800	-1.19632000
C	6.31377000	1.48409400	1.20211700
H	7.35984900	1.20204000	1.08820300
H	6.03788100	1.36606100	2.24912900
H	5.70446500	0.80239400	0.60999400
C	6.93104100	3.86844100	1.63038200
H	6.77988900	3.65030700	2.68697500
H	7.98869500	3.72920200	1.40313000
H	6.67167400	4.90971000	1.45866000
C	6.44846700	4.61232400	-1.14263800
H	7.47462300	4.89904600	-0.91467700
H	6.28538700	4.72708800	-2.21276900
H	5.77237100	5.29156300	-0.62584900
C	7.13506600	2.23340700	-1.48469400
H	7.12336100	2.45939300	-2.55010700
H	8.15316900	2.37224100	-1.11851900
H	6.85713700	1.19042900	-1.35695800
H	-0.02812700	1.84259200	1.89347300
H	-0.38133000	3.69905800	-1.94271700
C	2.20789700	4.13201500	-2.41943300
F	2.80441100	3.25439800	-3.24910100
F	3.13184300	5.06147600	-2.08761100
F	1.27108200	4.77433400	-3.15255400

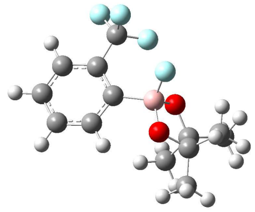

[**15o–F**]$^{-}$

C	0.42714100	2.29404300	1.02231900
C	1.80683100	2.43567800	0.95340300
C	2.43549700	3.00641700	-0.15835700
C	1.61404400	3.46479900	-1.20191100
C	0.23214100	3.33186200	-1.13436000
C	-0.36202400	2.73963100	-0.02720000
H	2.42082600	2.09829200	1.77710000
H	-1.43789800	2.63890000	0.01439600
C	6.17684700	3.16081000	-0.75944800
C	6.07873700	2.93443200	0.79062600
B	3.99697400	3.05266000	-0.12609000
O	4.67497900	3.21975100	1.05297100
O	4.82055300	2.86310800	-1.19632000
C	6.31377000	1.48409400	1.20211700
H	7.35984900	1.20204000	1.08820300
H	6.03788100	1.36606100	2.24912900
H	5.70446500	0.80239400	0.60999400
C	6.93104100	3.86844100	1.63038200
H	6.77988900	3.65030700	2.68697500
H	7.98869500	3.72920200	1.40313000
H	6.67167400	4.90971000	1.45866000
C	6.44846700	4.61232400	-1.14263800
H	7.47462300	4.89904600	-0.91467700
H	6.28538700	4.72708800	-2.21276900
H	5.77237100	5.29156300	-0.62584900
C	7.13506600	2.23340700	-1.48469400
H	7.12336100	2.45939300	-2.55010700
H	8.15316900	2.37224100	-1.11851900
H	6.85713700	1.19042900	-1.35695800
H	-0.02812700	1.84259200	1.89347300
H	-0.38133000	3.69905800	-1.94271700
C	2.20789700	4.13201500	-2.41943300
F	2.80441100	3.25439800	-3.24910100
F	3.13184300	5.06147600	-2.08761100
F	1.27108200	4.77433400	-3.15255400

Plots of NMR spectra

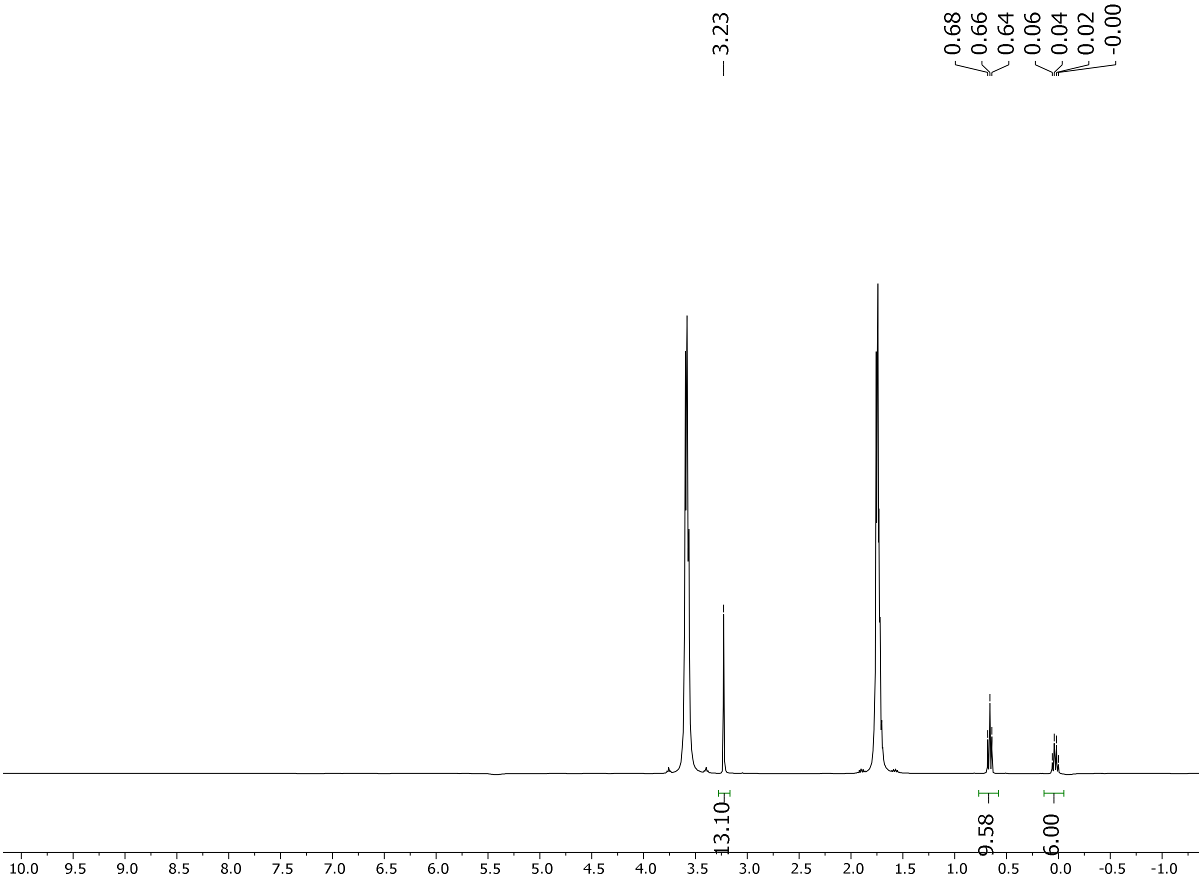

Figure S14: ^{1}H NMR spectrum of $[NMe_4][FBEt_3]$ (400 MHz) in THF.

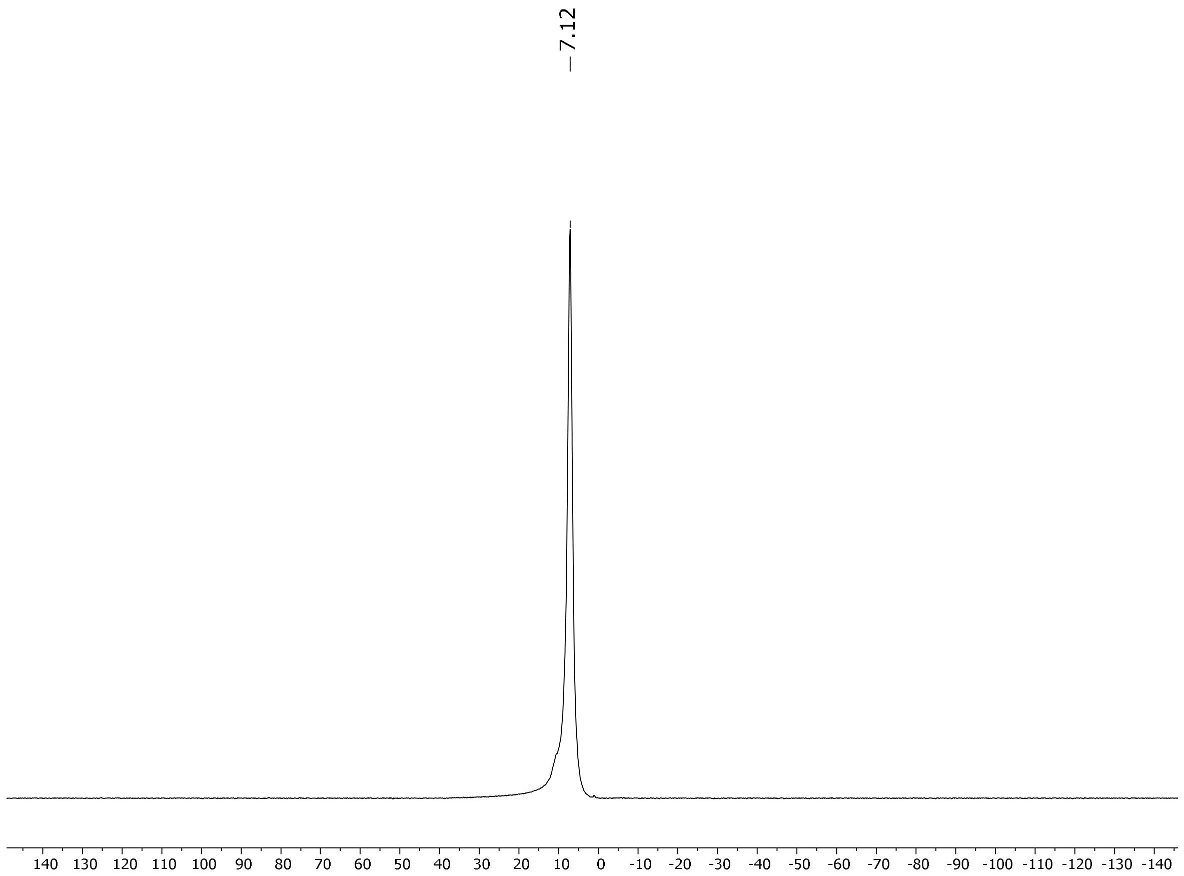

Figure S15: $^{11}B\{^{1}H\}$ NMR spectrum (128 MHz) of $[NMe_4][FBEt_3]$ in THF.

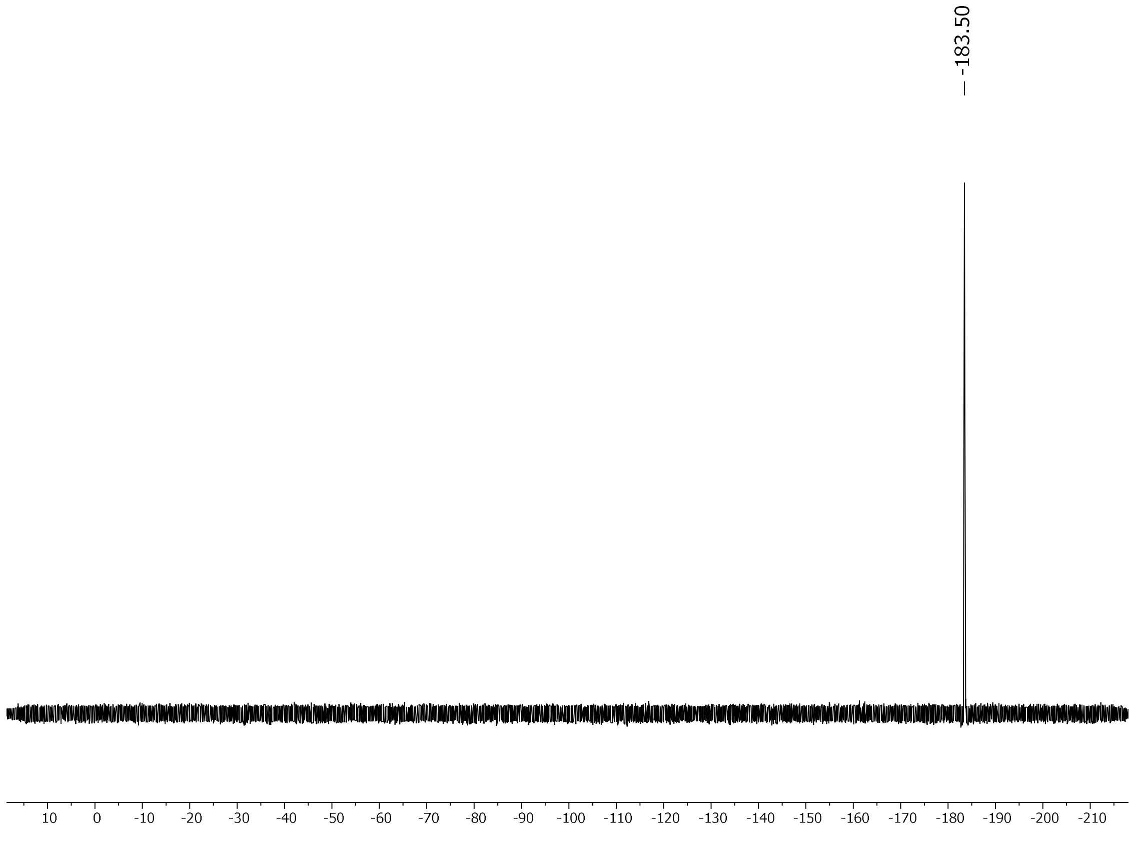

Figure S16: $^{19}F\{^{1}H\}$ NMR spectrum (376 MHz) of $[NMe_4][FBEt_3]$ in THF.

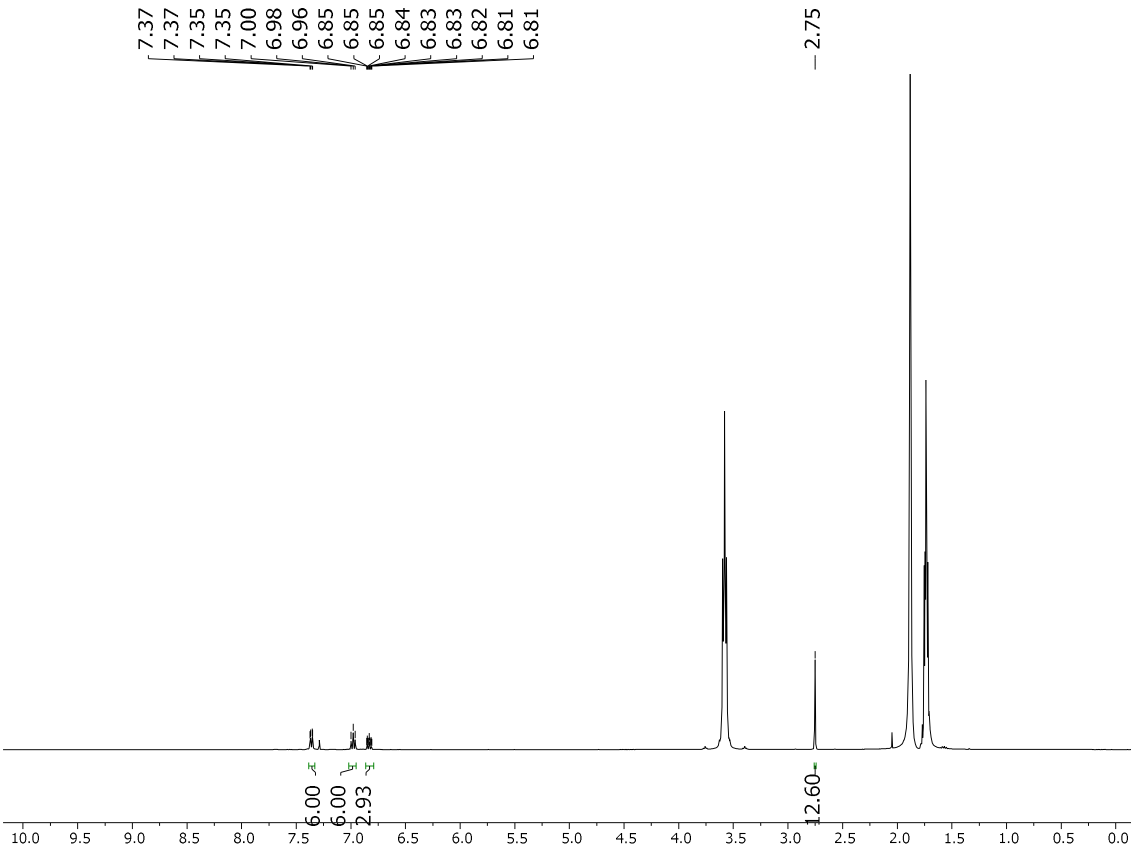

Figure S17: ^{1}H NMR spectrum (400 MHz) of $[NMe_4][FBPh_3]$ in THF-MeCN (1:1) mixed solvents.

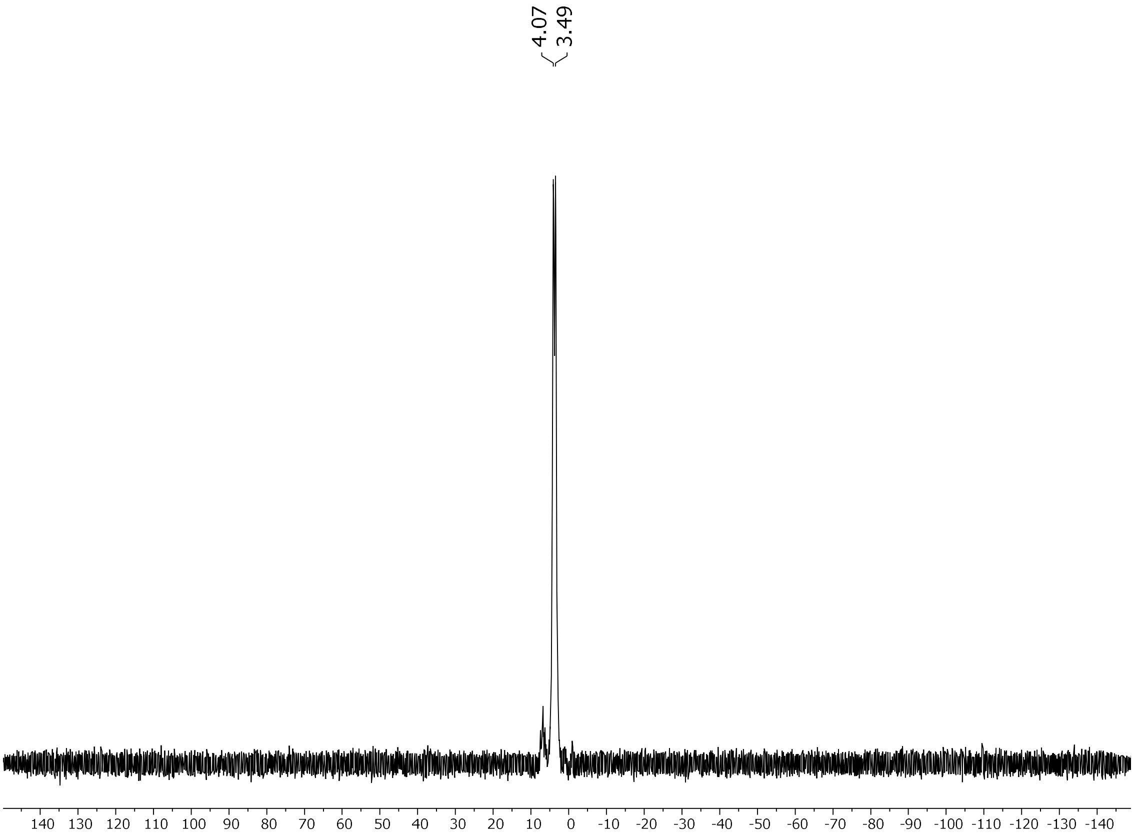

Figure S18: $^{11}B\{^{1}H\}$ NMR spectrum (128 MHz) of $[NMe_4][FBPh_3]$ in THF-MeCN (1:1) mixed solvents.

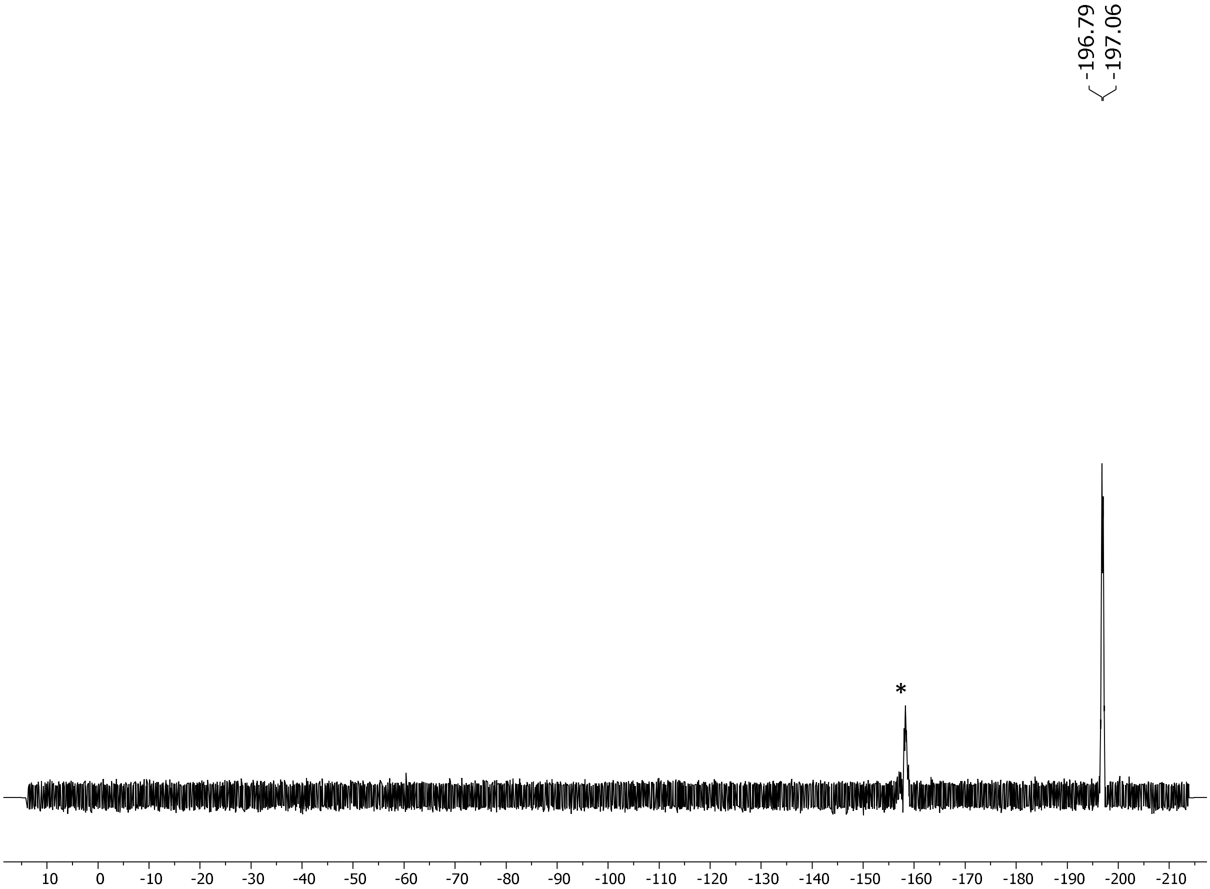

Figure S19: $^{19}F\{^{1}H\}$ NMR spectrum (376 MHz) of $[NMe_4][FBPh_3]$ in THF-MeCN (1:1) mixed solvents. * denotes impurity.

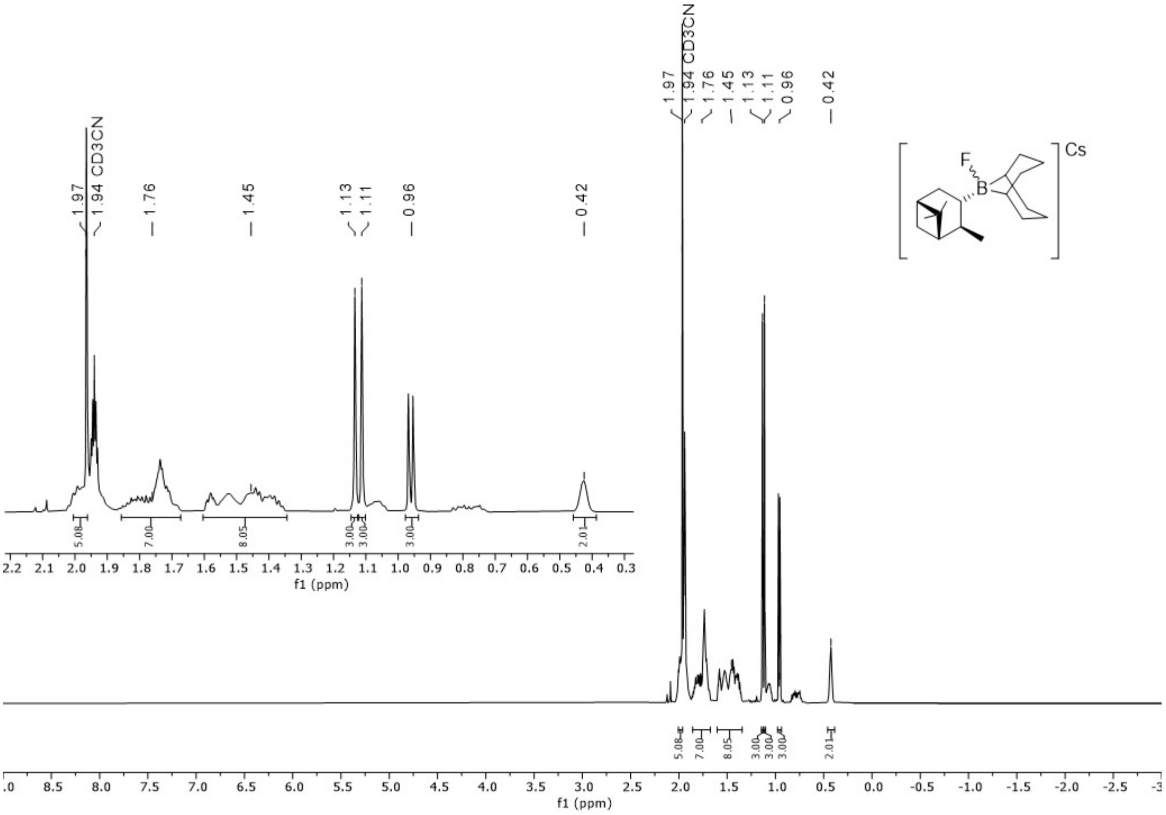

Figure S20: ^{1}H NMR spectrum (500 MHz) of Cs[**5**–F] in CD_3CN.

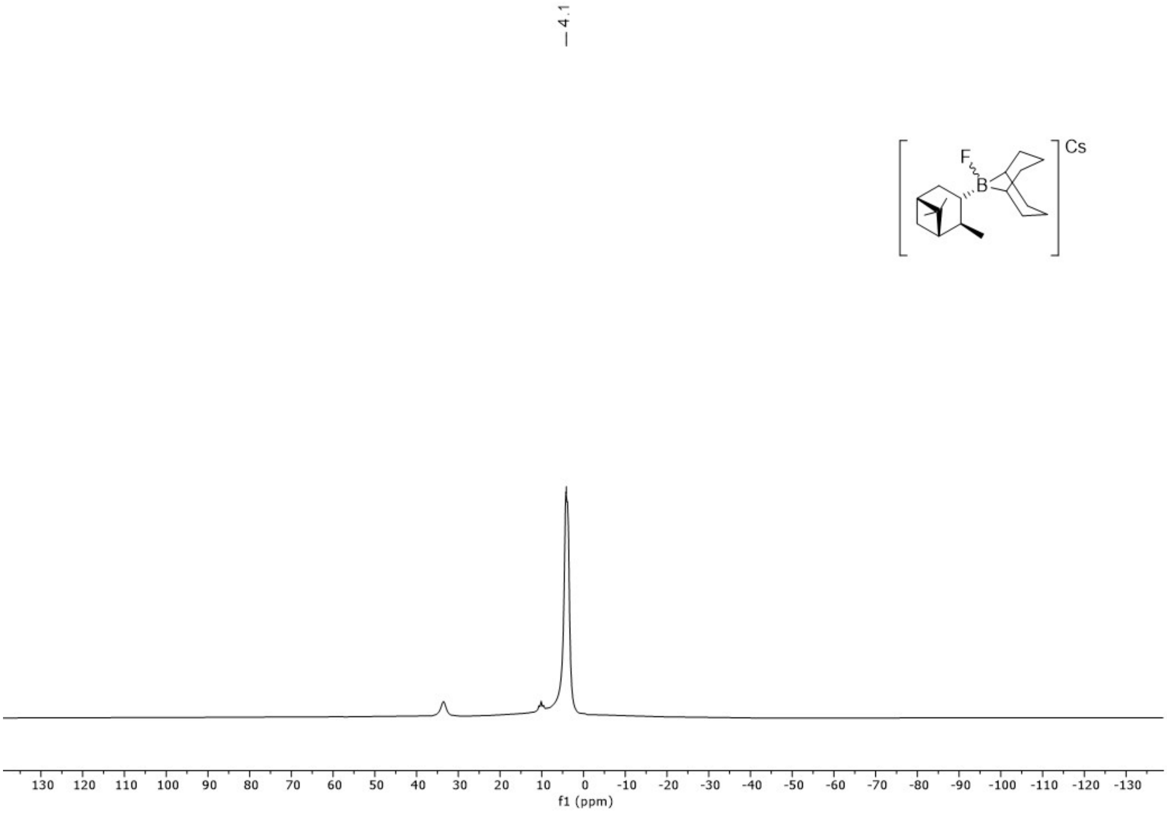

Figure S21: ^{11}B NMR spectrum (160 MHz) of Cs[**5**–F] in CD_3CN.

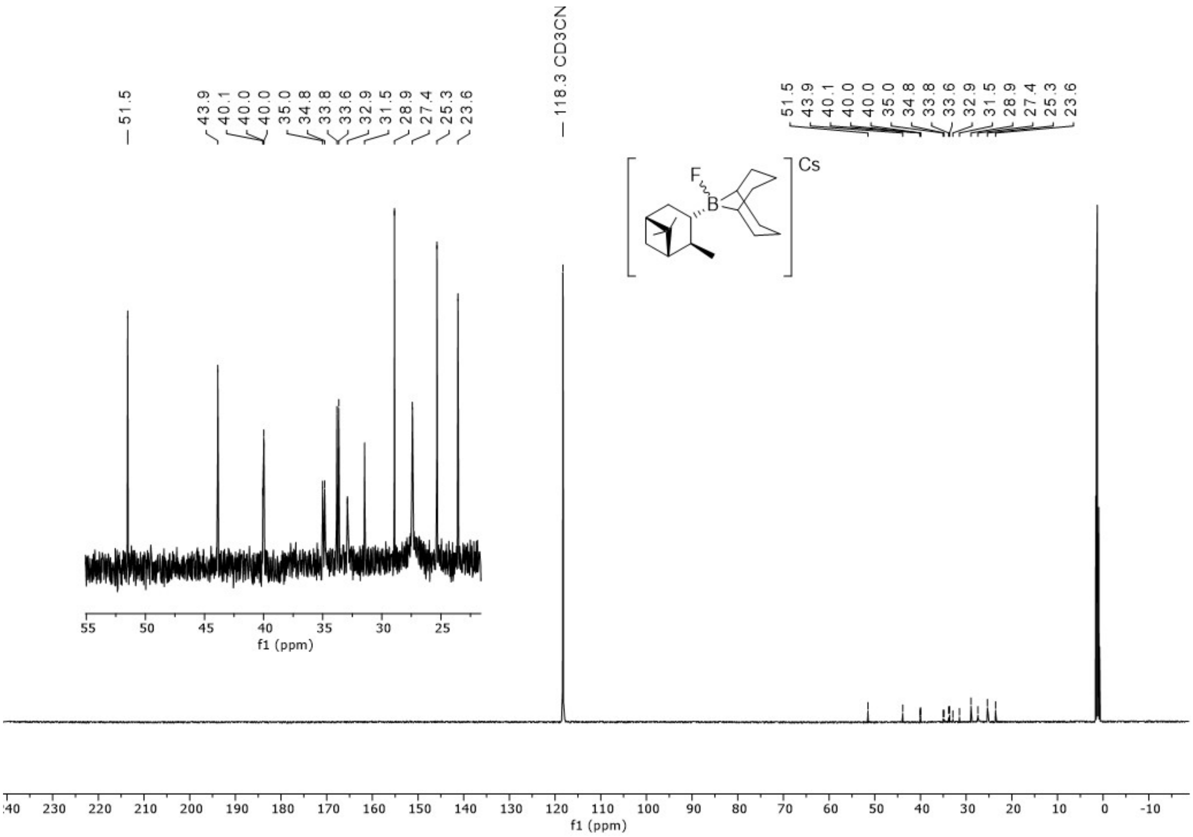

Figure S22: $^{13}C\{^{1}H\}$ NMR spectrum (126 MHz) of Cs[**5**–F] in CD_3CN.

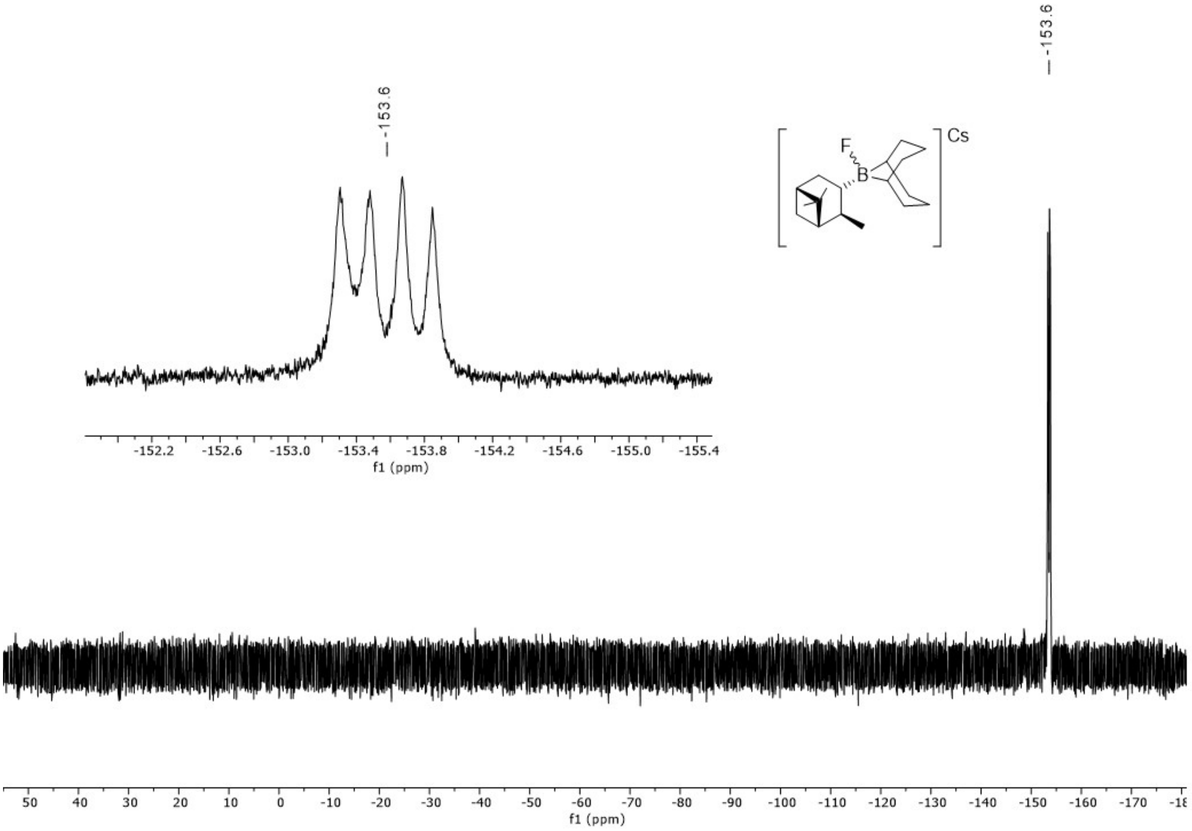

Figure S23: ^{19}F NMR spectrum (471 MHz) of Cs[**5**–F] in CD_3CN.

Plots of CBS catalyst NMR Spectra

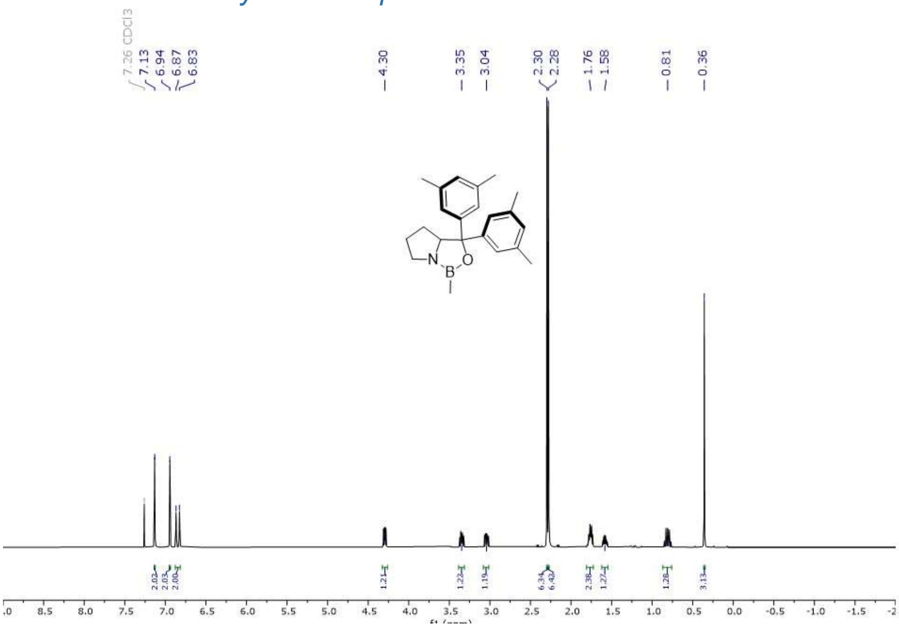

Figure S24: ^{1}H NMR spectrum (500 MHz) of the depicted CBS catalyst in $CDCl_3$.

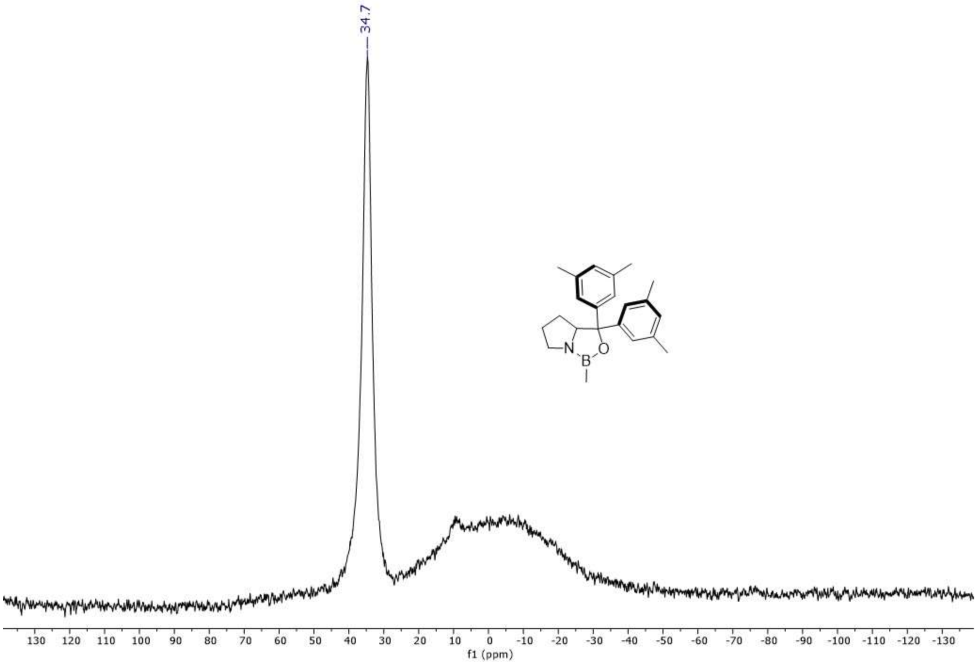

Figure S25: $^{11}B\{^{1}H\}$ NMR spectrum (160 MHz) of the depicted CBS catalyst in $CDCl_3$

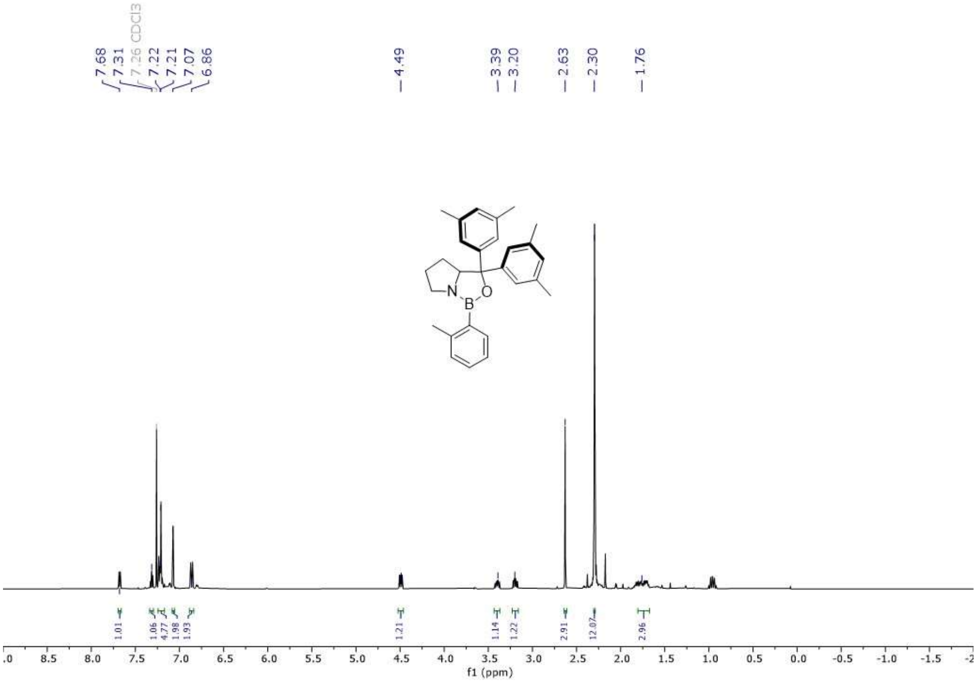

Figure S26: ^{1}H NMR spectrum (500 MHz) of the depicted CBS catalyst in $CDCl_3$.

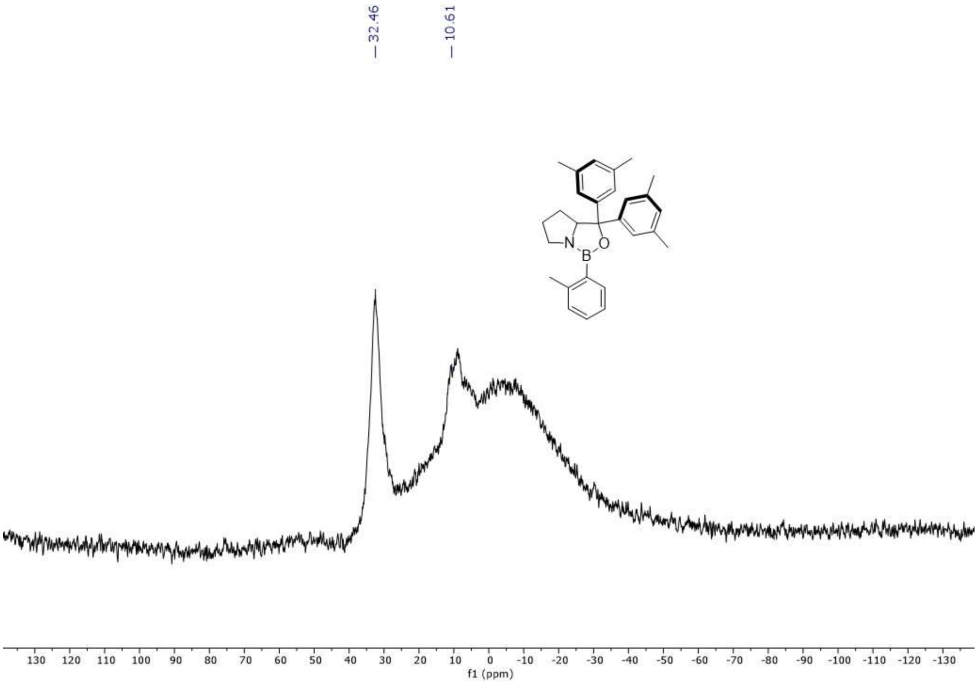

Figure S27: $^{11}B\{^{1}H\}$ NMR spectrum (160 MHz) of the depicted CBS catalyst in $CDCl_3$

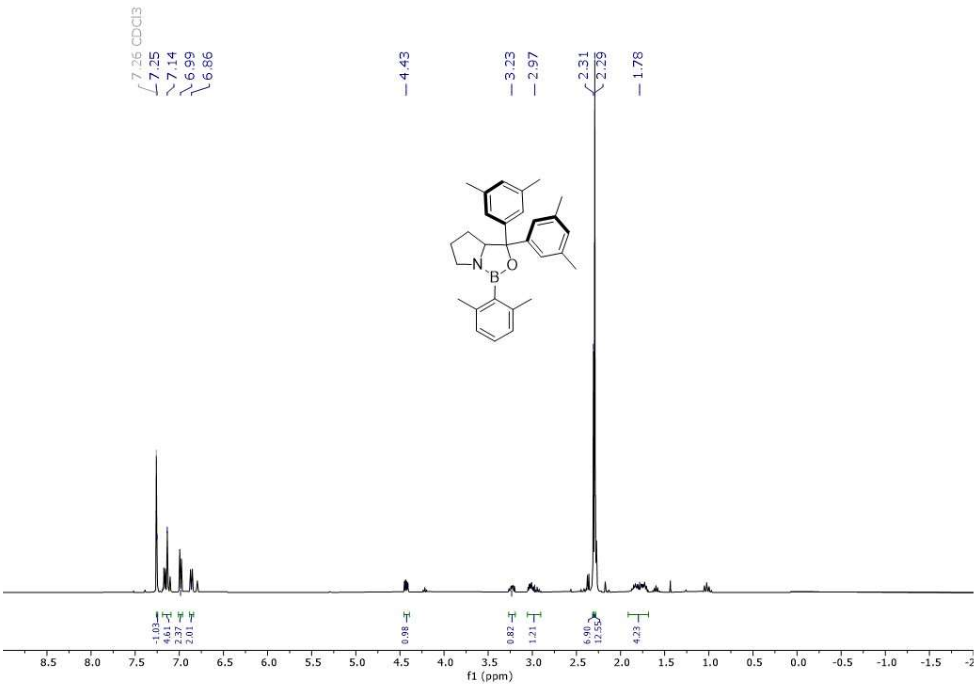

Figure S28: ^{1}H NMR spectrum (500 MHz) of the depicted CBS catalyst in $CDCl_3$.

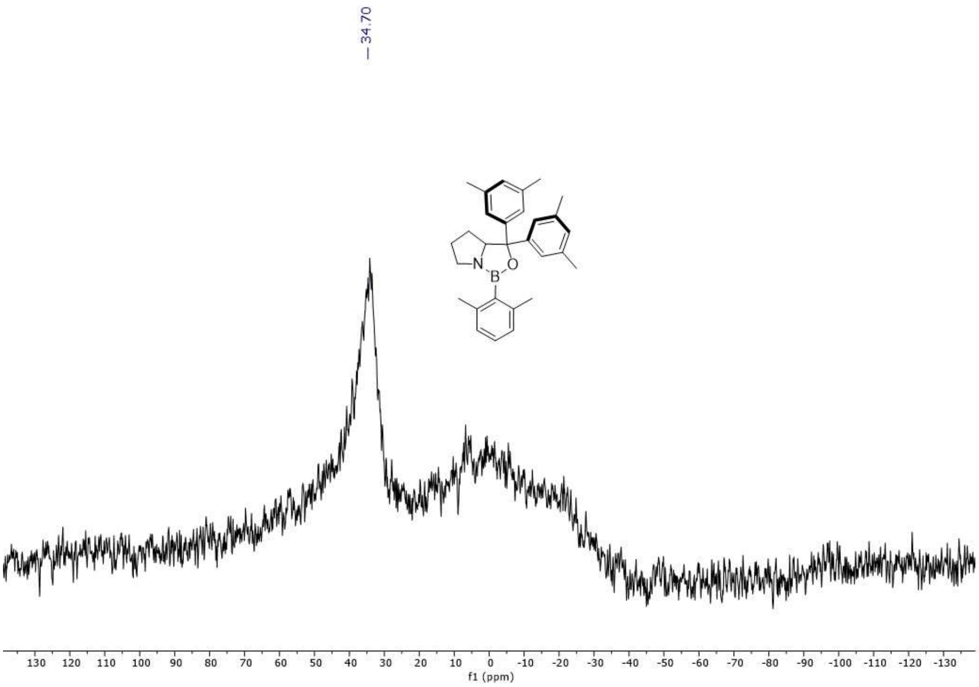

Figure S29: $^{11}B\{^{1}H\}$ NMR spectrum (160 MHz) of the depicted CBS catalyst in $CDCl_3$

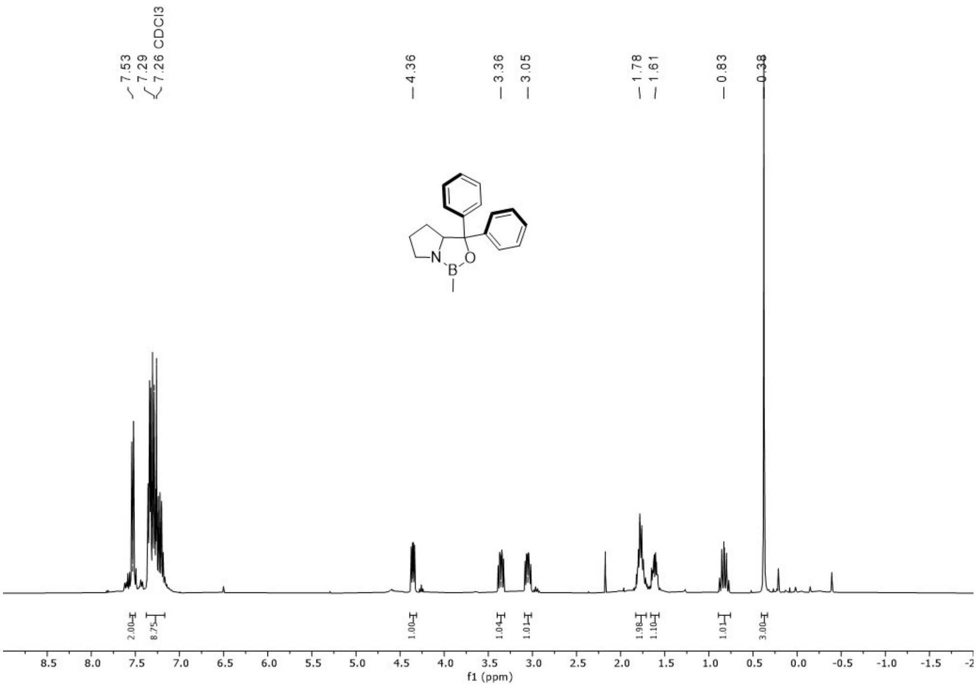

Figure S30: ${}^{1}H$ NMR spectrum (500 MHz) of self-made **11** in $CDCl_3$.

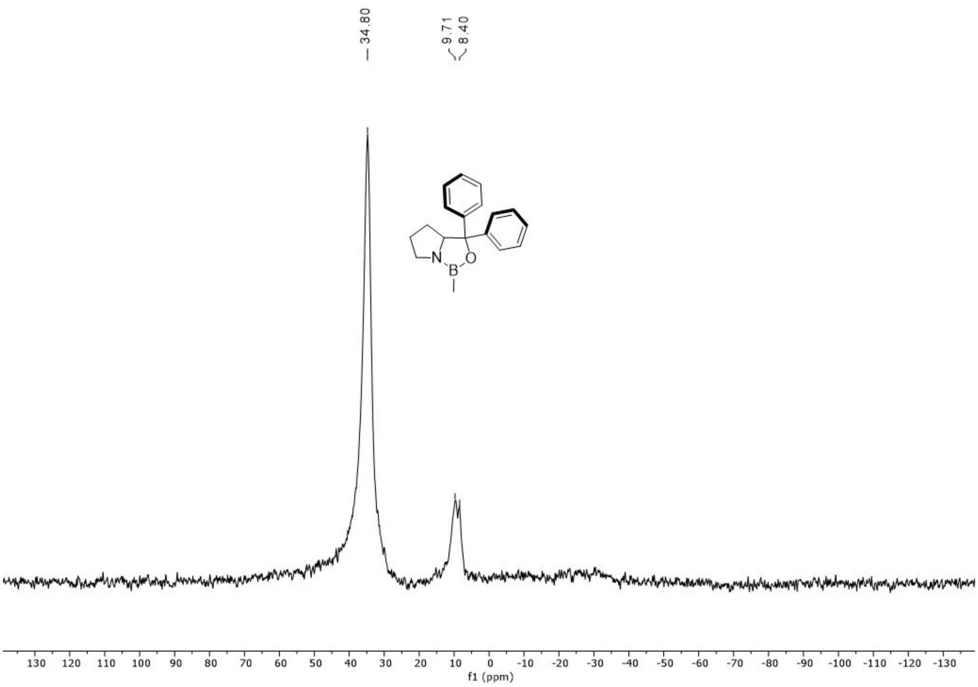

Figure S31: ${}^{11}B\{{}^{1}H\}$ NMR spectrum (160 MHz) of self-made **11** in $CDCl_3$

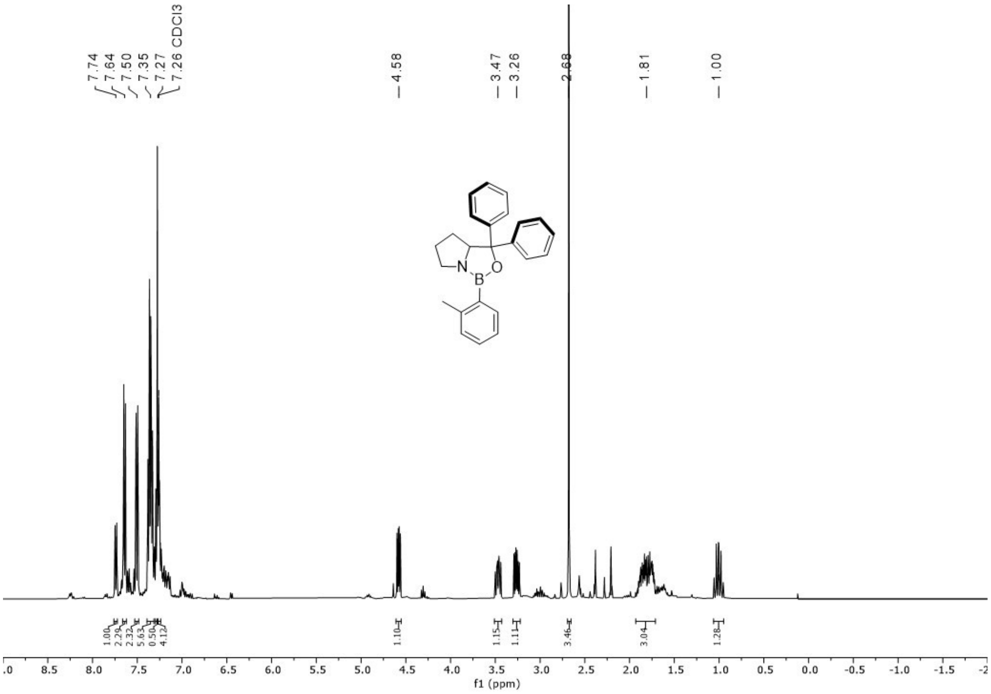

Figure S32: ^{1}H NMR spectrum (500 MHz) of self-made **4** in $CDCl_3$.

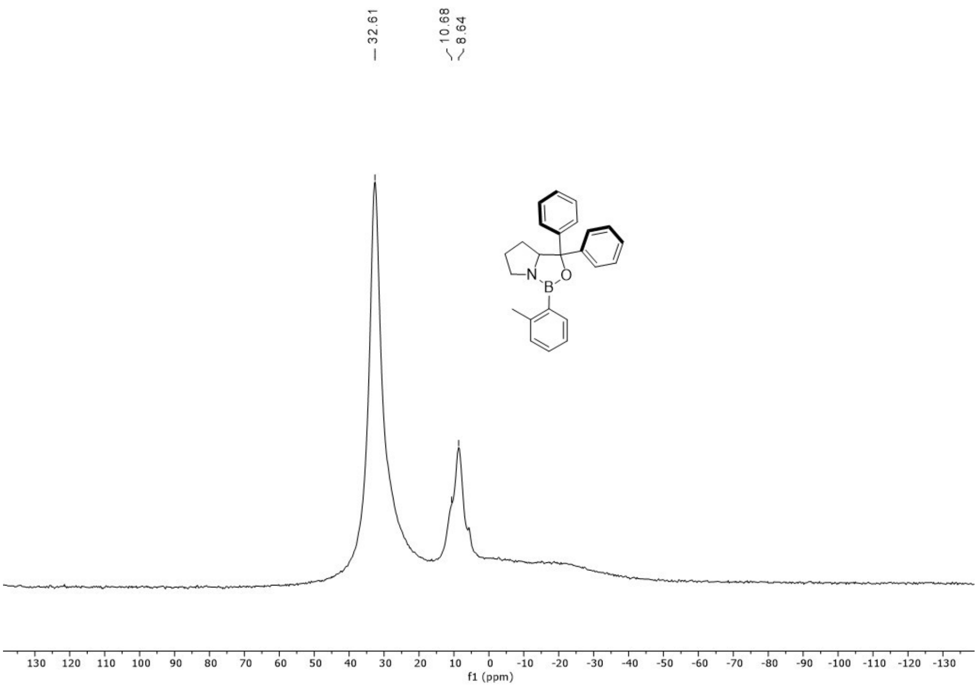

Figure S33: $^{11}B\{^{1}H\}$ NMR spectrum (160 MHz) of self-made **4** in $CDCl_3$

*Plots of NMR spectra of Cs[**1**–F] complexes*

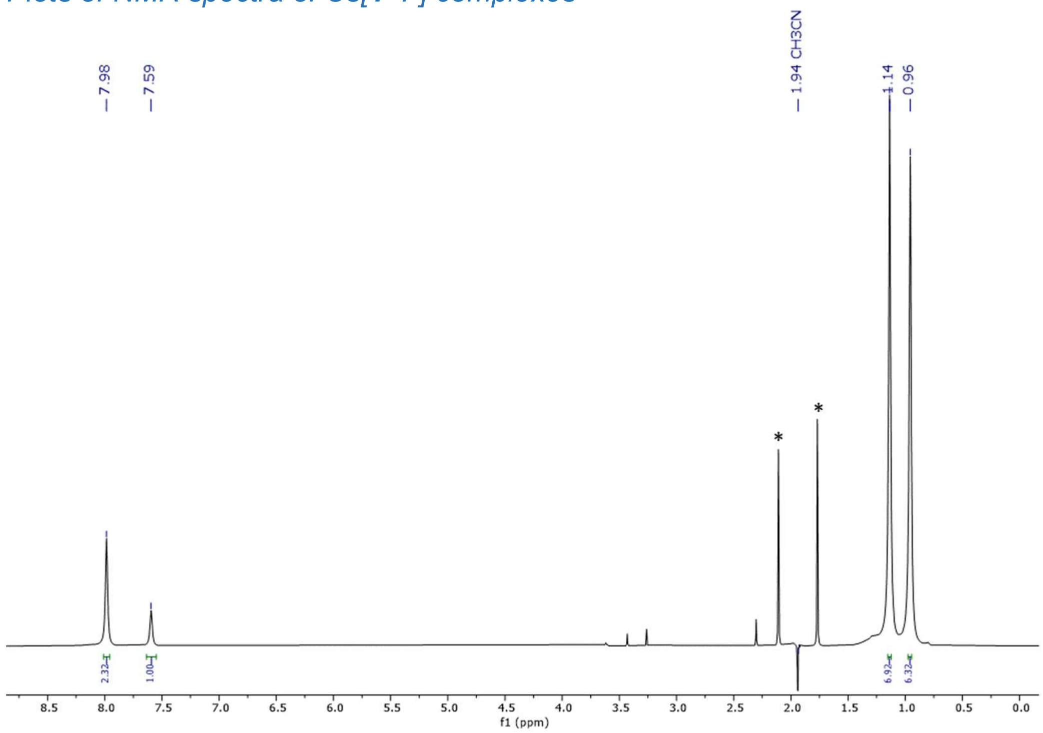

Figure S34: ^{1}H NMR spectrum of Cs[**1**–F] (400 MHz) in CH_3CN (solvent suppression pulse programme). Resonances marked with an asterisk are ^{13}C satellite signals of CH_3CN.

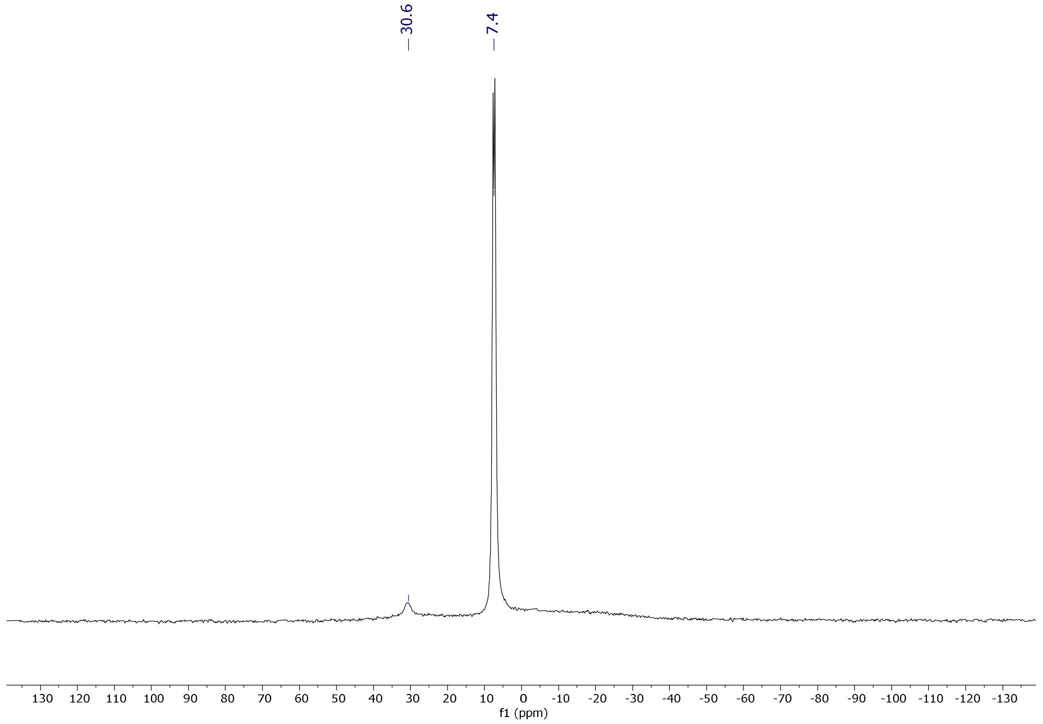

Figure S35: $^{11}B\{^{1}H\}$ NMR spectrum of Cs[**1**–F] (128 MHz) in CH_3CN.

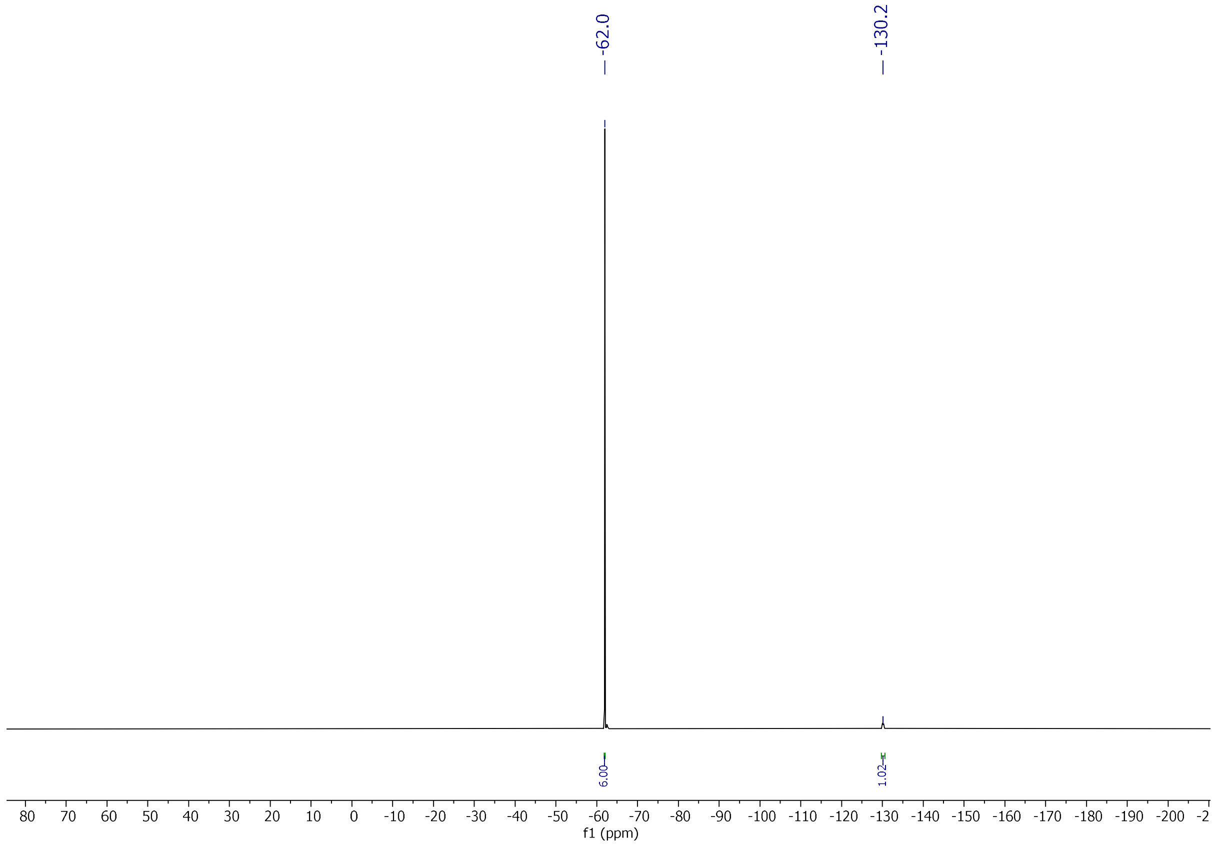

Figure S36: ^{19}F NMR spectrum of Cs[**1**–F] (376 MHz) in CH_3CN.

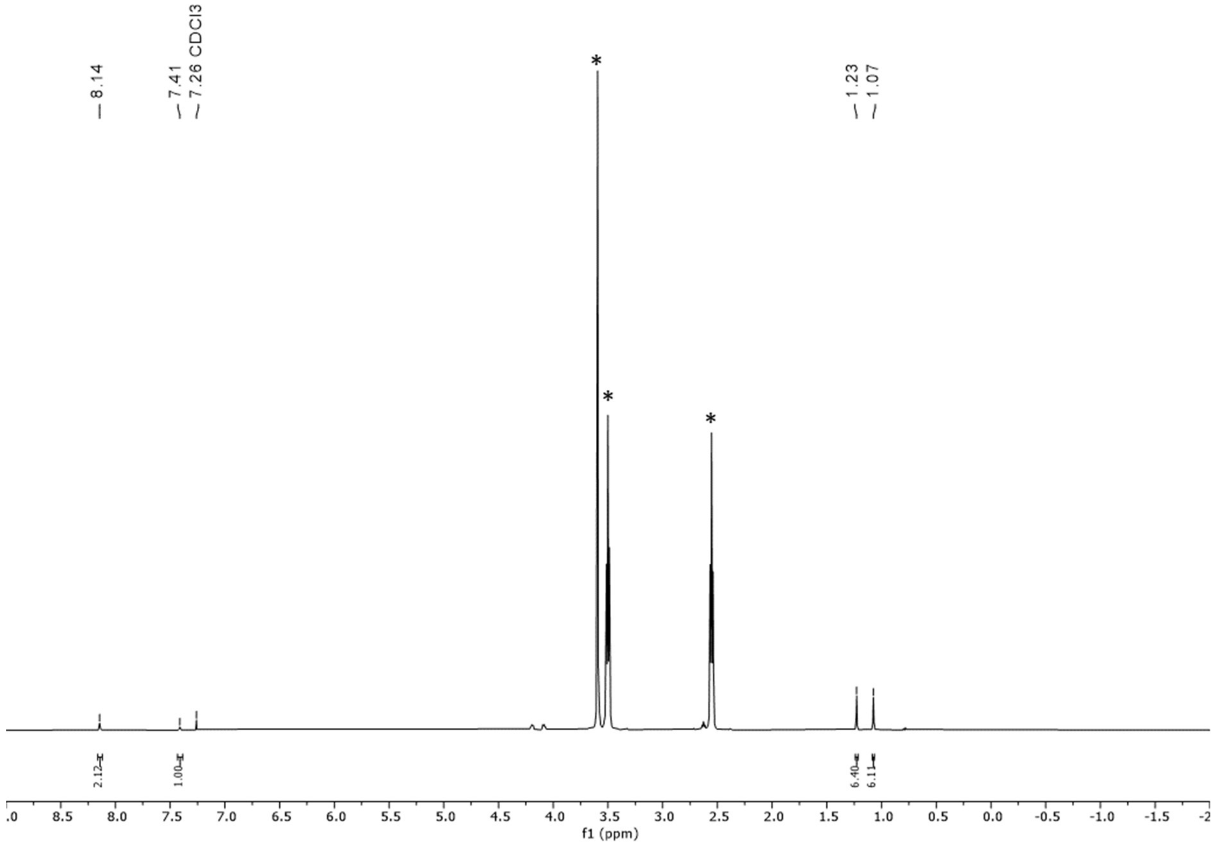

Figure S37: Figure S 38: ^{1}H NMR spectrum of Cs·[2.2.2][**1**–F] (400 MHz) in $CDCl_3$. Resonances marked with an asterisk originate from the large excess of [2.2.2]-cryptand.

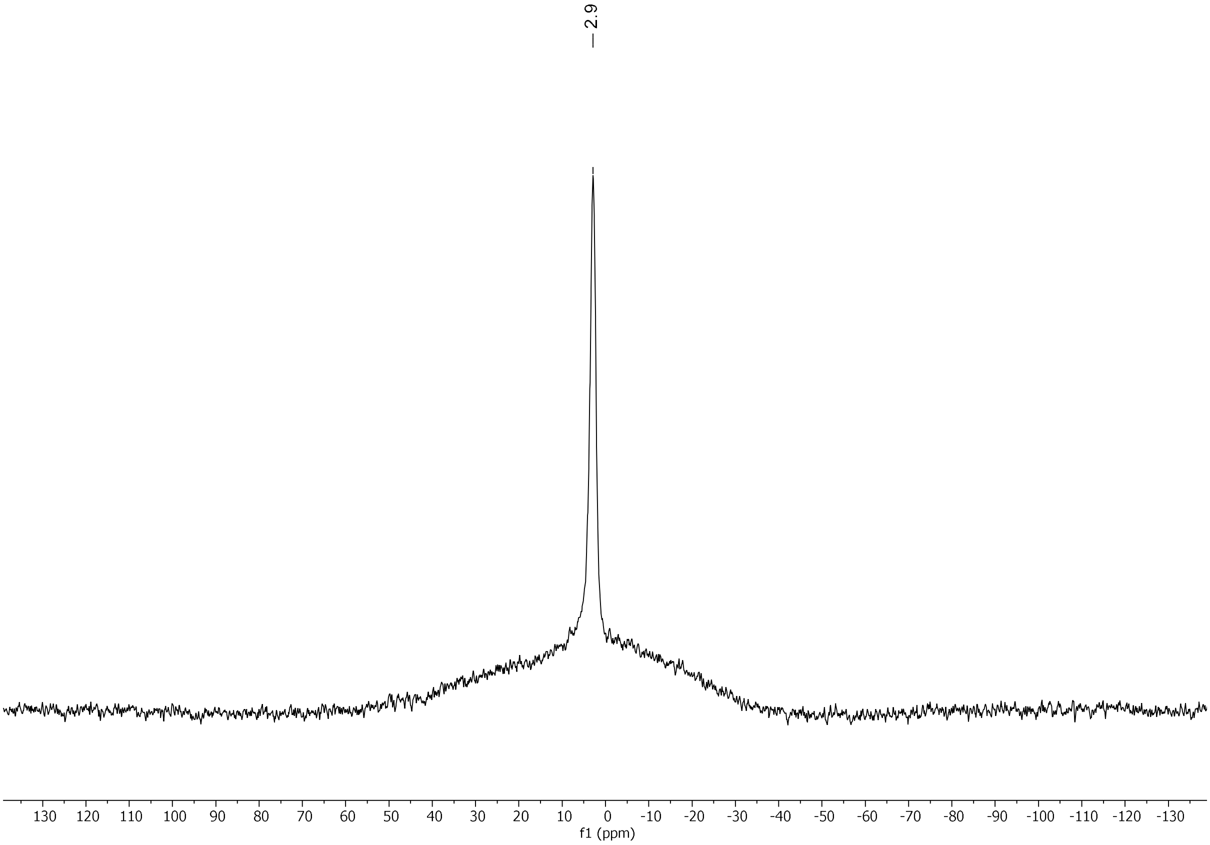

Figure S39: $^{11}B\{^{1}H\}$ NMR spectrum of Cs·[2.2.2][**1**–F] (128 MHz) in $CDCl_3$.

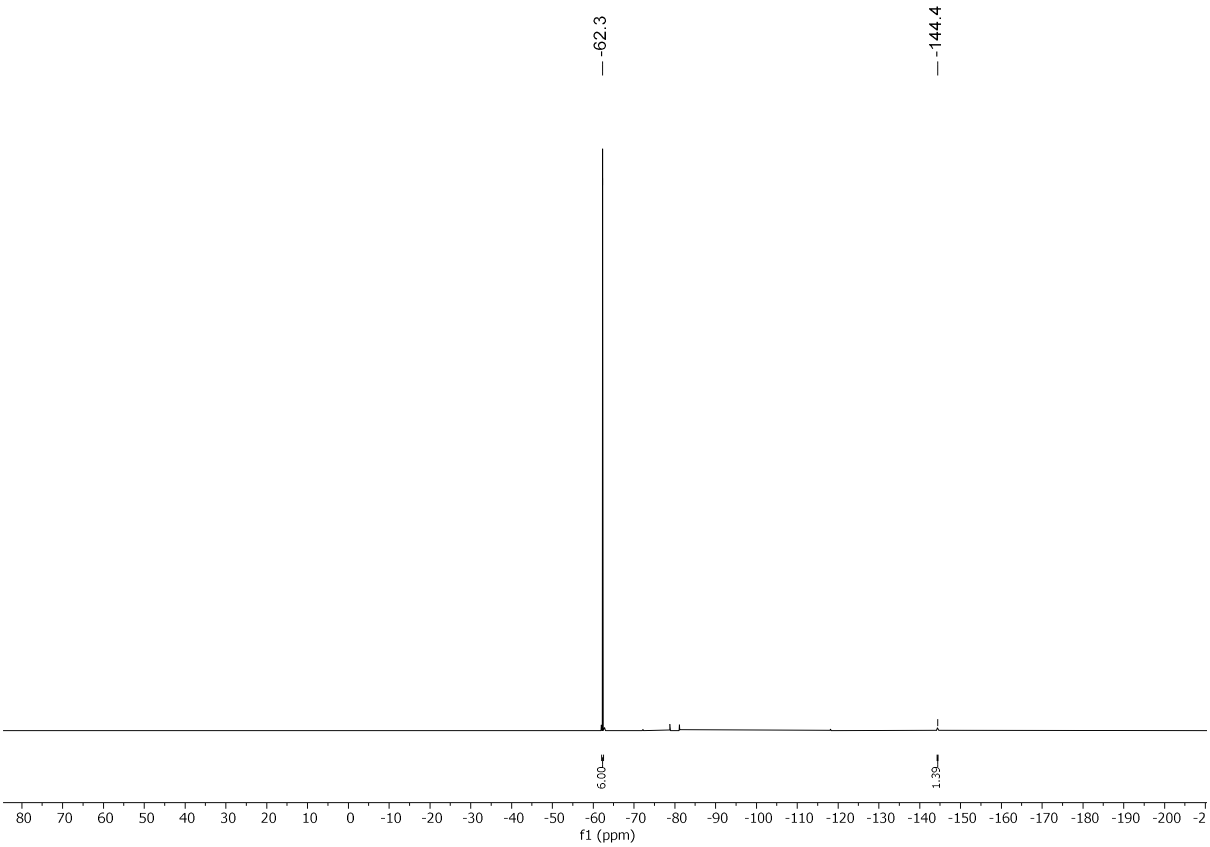

Figure S40: ^{19}F NMR spectrum of Cs·[2.2.2][**1**–F] (376 MHz) in CH_3CN.

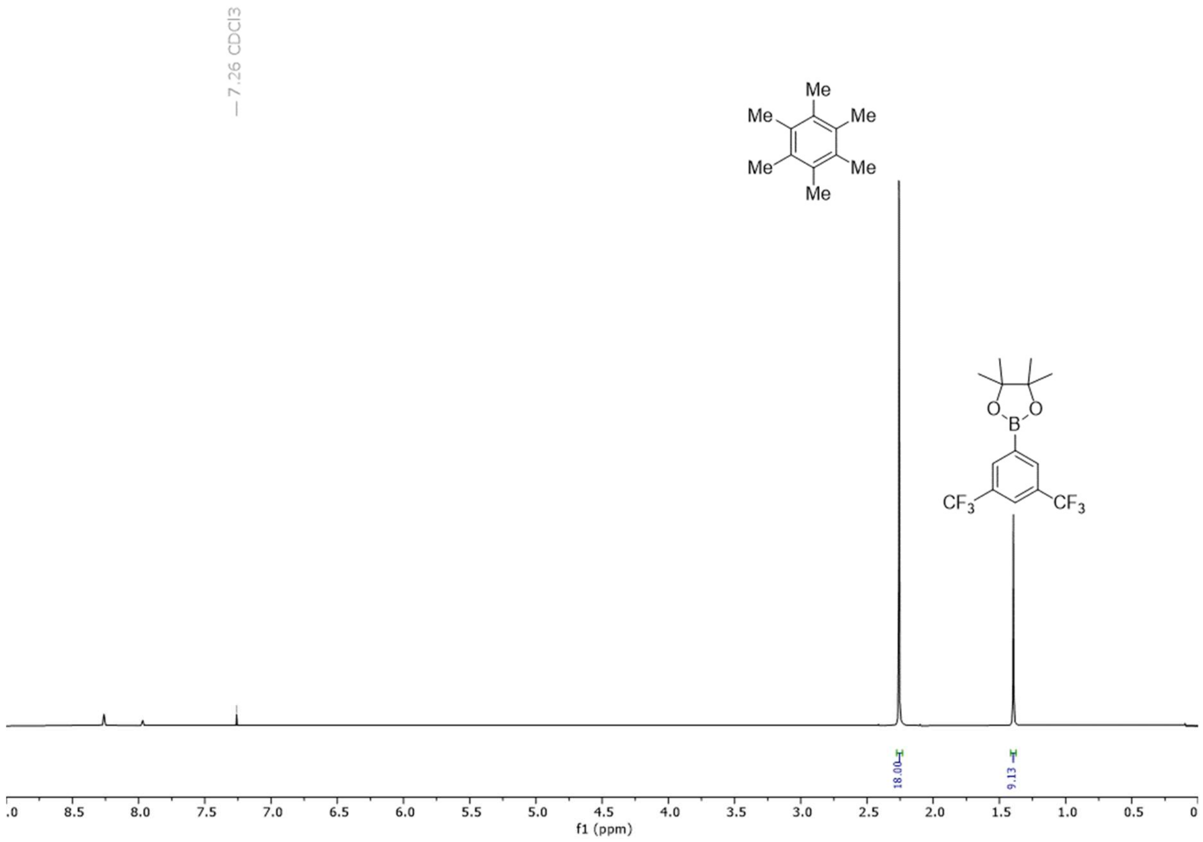

Figure S41: ^{1}H NMR spectrum (400 MHz) of **1** in $CDCl_3$ over excess CsF versus hexamethylbenzene (1 eq) as internal standard.

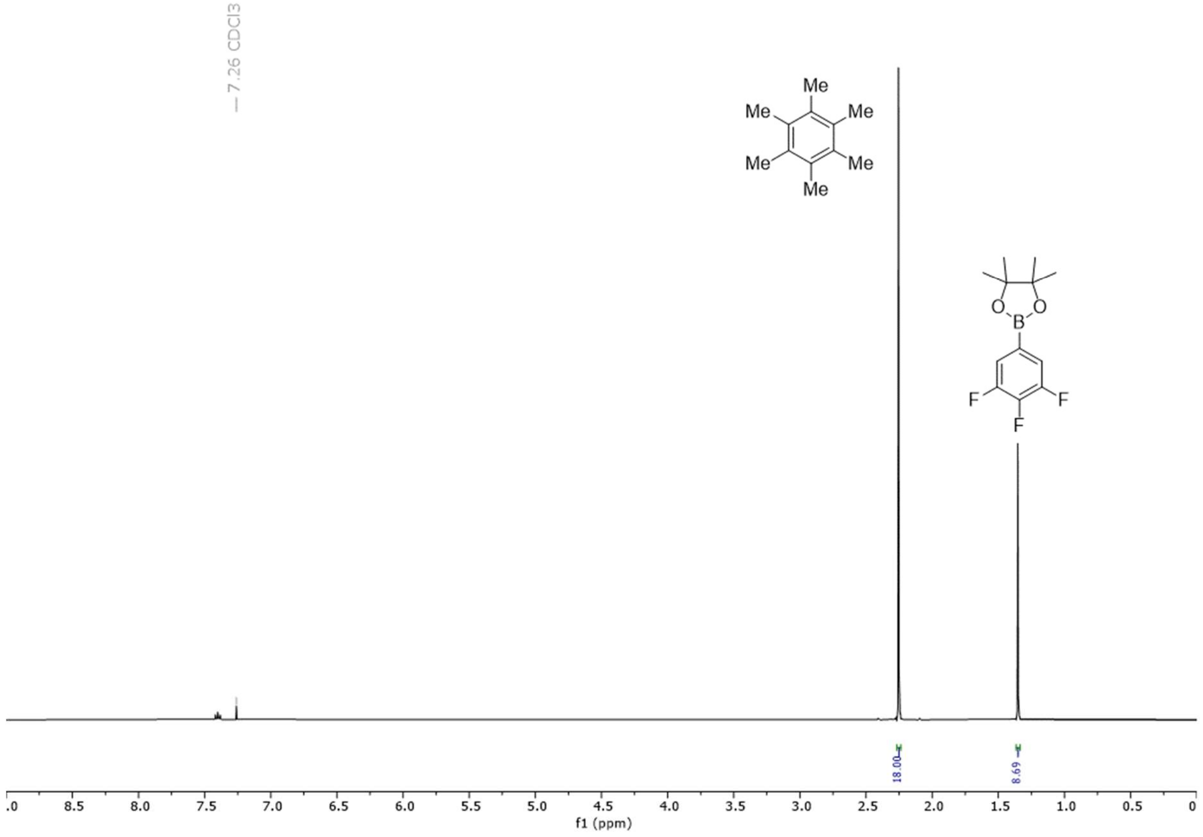

Figure S42: ^{1}H NMR spectrum (400 MHz) of **2** in $CDCl_3$ over excess CsF versus hexamethylbenzene (1 eq) as internal standard.

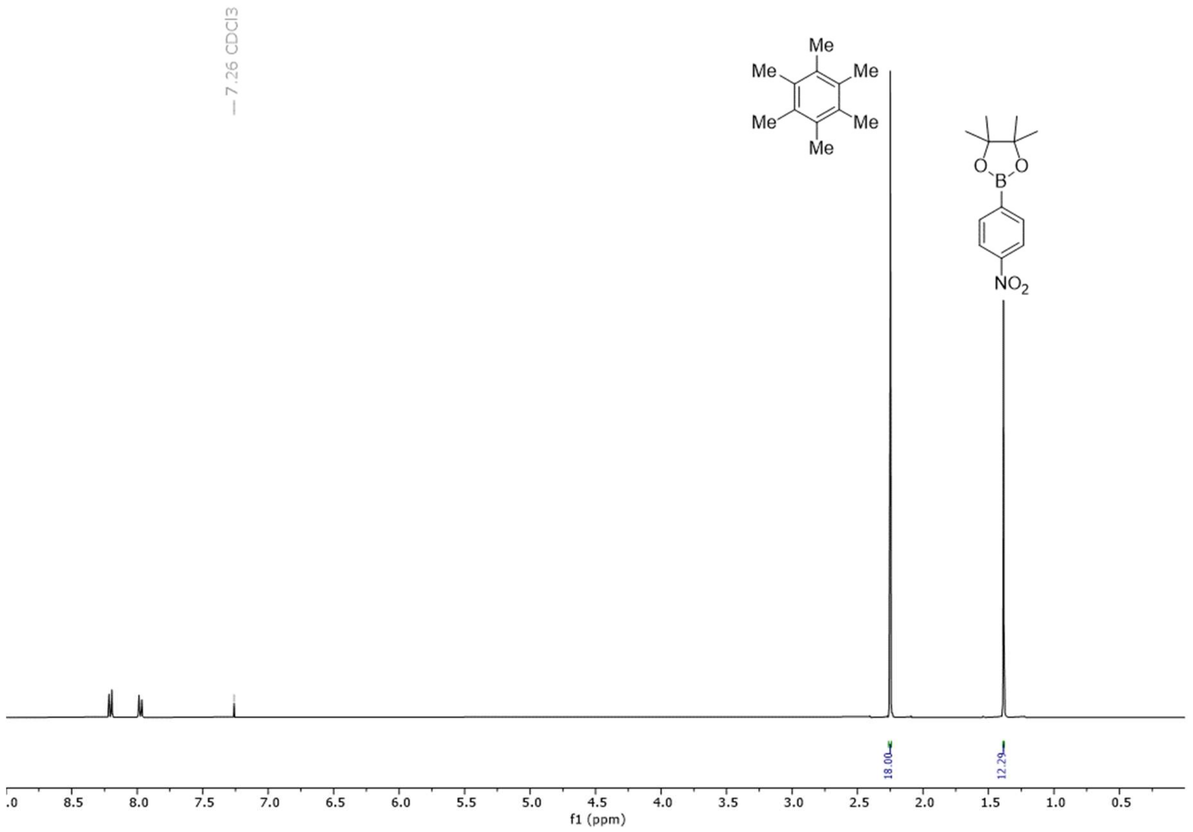

Figure S43: ^{1}H NMR spectrum (400 MHz) of **3** in $CDCl_3$ over excess CsF versus hexamethylbenzene (1 eq) as internal standard.

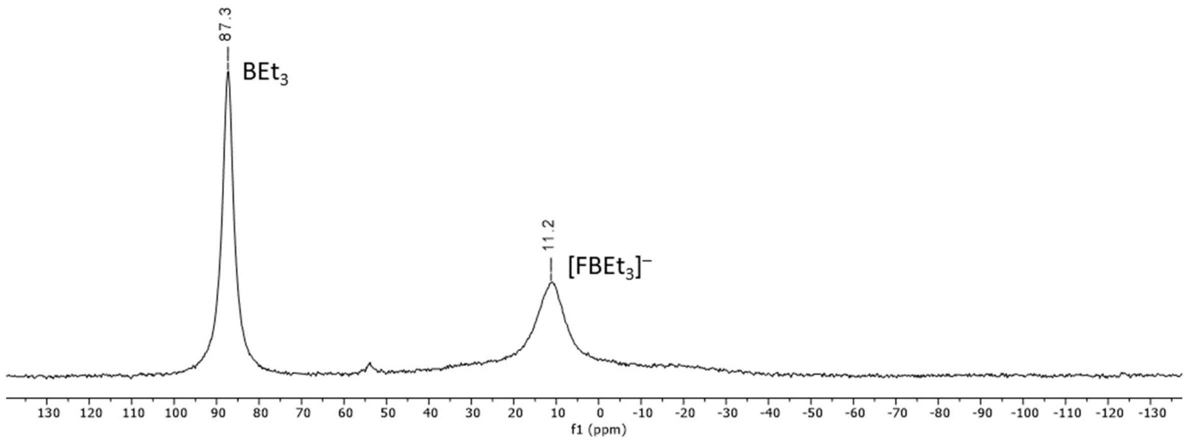

Figure S44: ^{11}B{^{1}H} NMR spectrum (161 MHz) of $Cs[FBEt_3]$ / BEt_3 in $CDCl_3$.

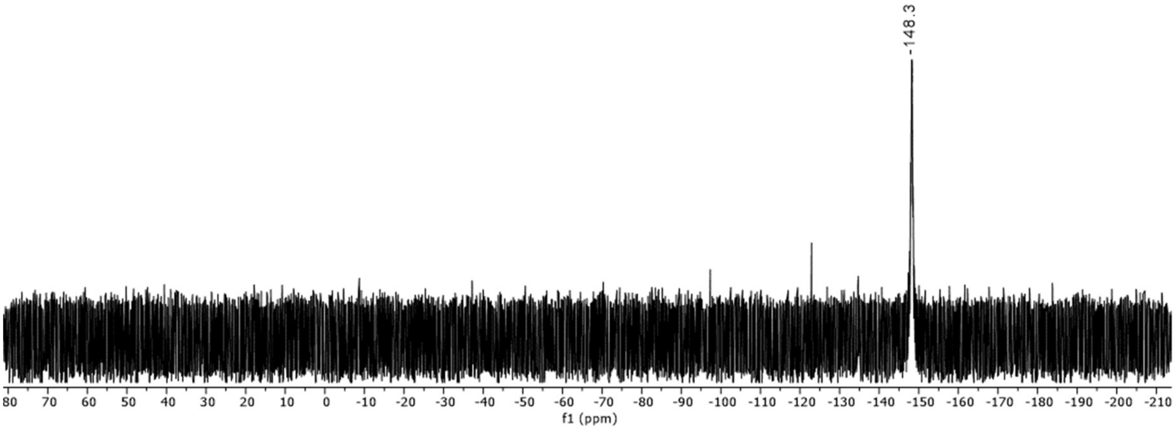

Figure S45: $^{19}F\{^{1}H\}$ NMR spectrum (470 MHz) of $Cs[FBEt_3]$ in $CDCl_3$.

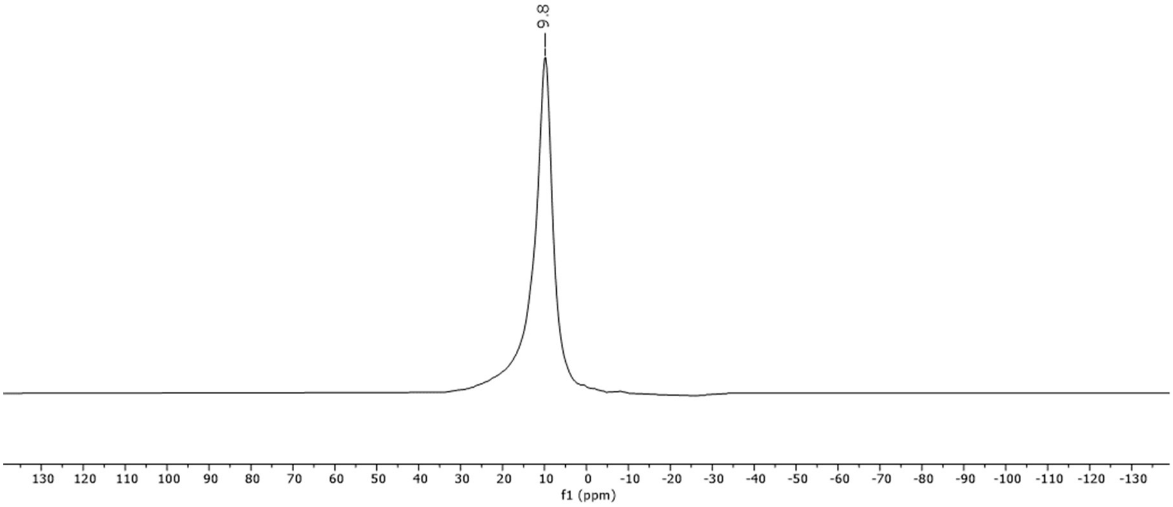

Figure S46: $^{11}B\{^{1}H\}$ NMR spectrum (161 MHz) of $Cs[FBEt_3]$ in CD_2Cl_2.

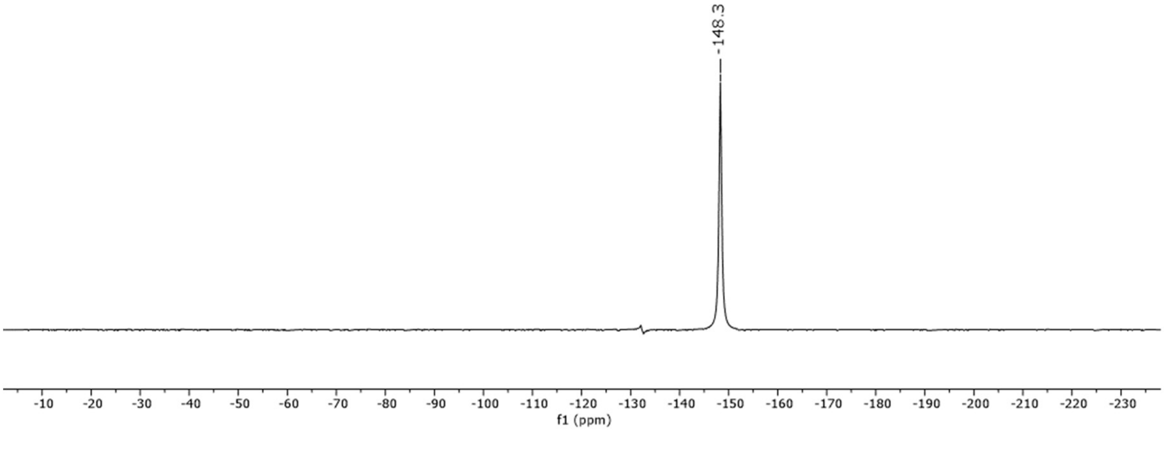

Figure S47: $^{19}F\{^{1}H\}$ NMR spectrum (470 MHz) of $Cs[FBEt_3]$ in CD_2Cl_2.

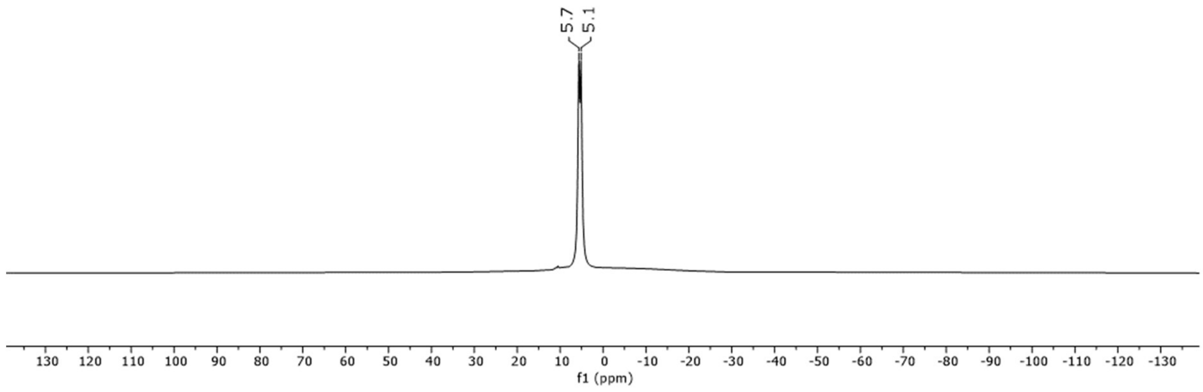

Figure S48: $^{11}B\{^{1}H\}$ NMR spectrum (161 MHz) of Cs[$FBEt_3$] in MeCN.

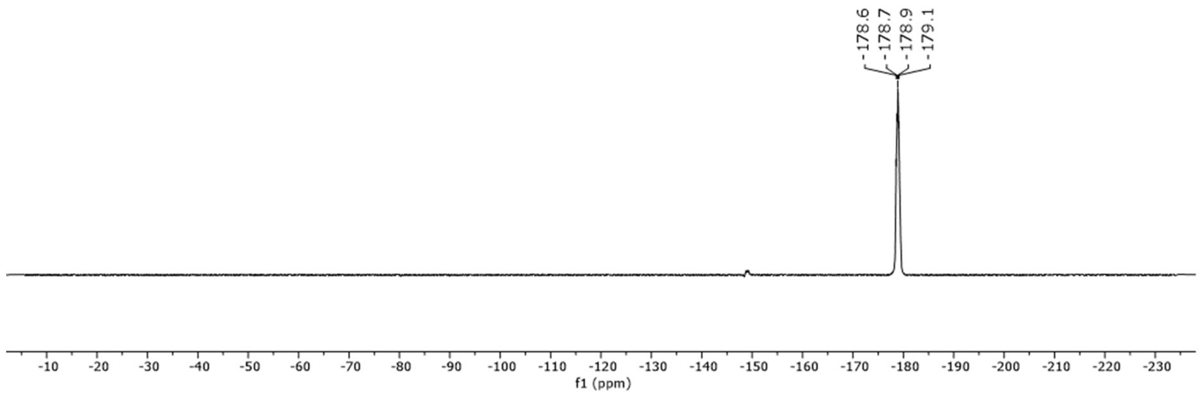

Figure S49: $^{19}F\{^{1}H\}$ NMR spectrum (470 MHz) of Cs[$FBEt_3$] in MeCN..

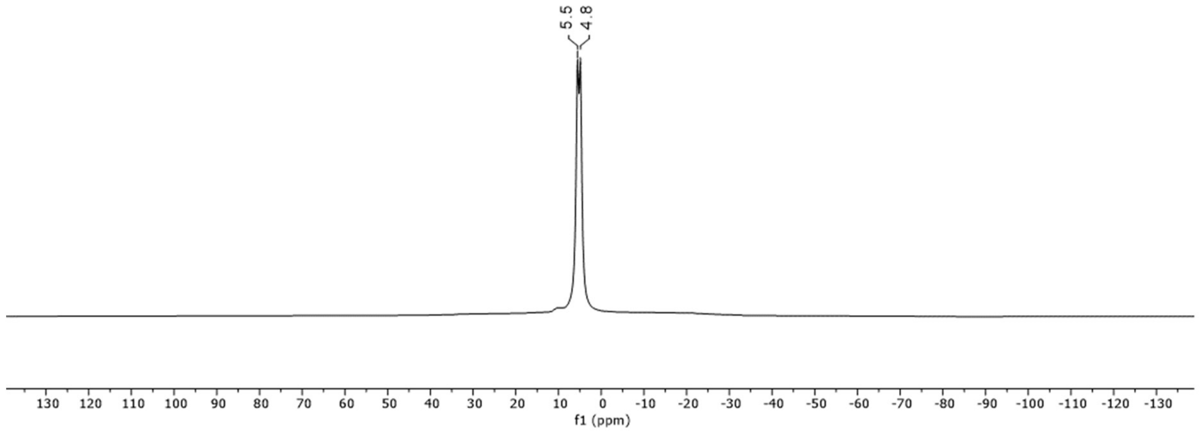

Figure S50: $^{11}B\{^{1}H\}$ NMR spectrum (161 MHz) of Cs·[2.2.2]-cryptand-[$FBEt_3$] in CD_2Cl_2.

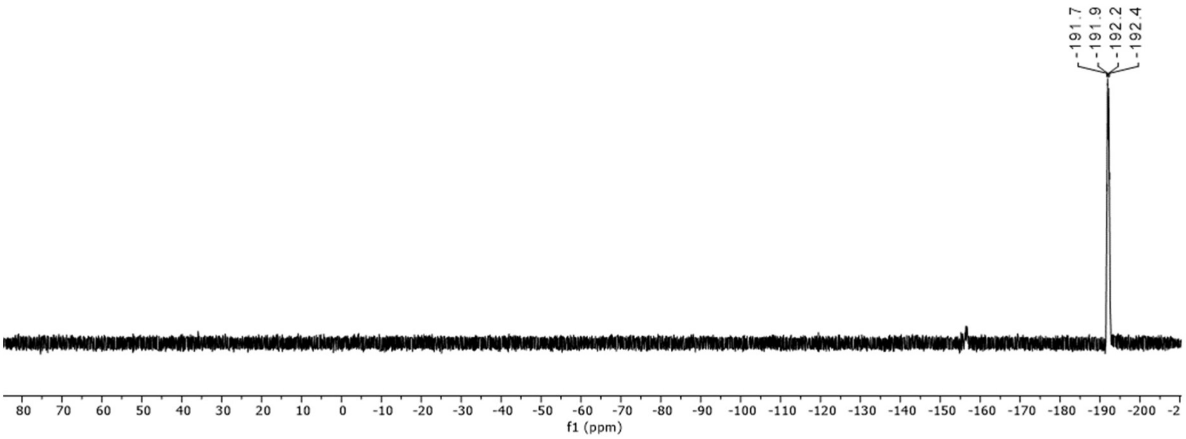

Figure S51: $^{19}F\{^{1}H\}$ NMR spectrum (470 MHz) of Cs·[2.2.2]-cryptand-[$FBEt_3$] in CD_2Cl_2.

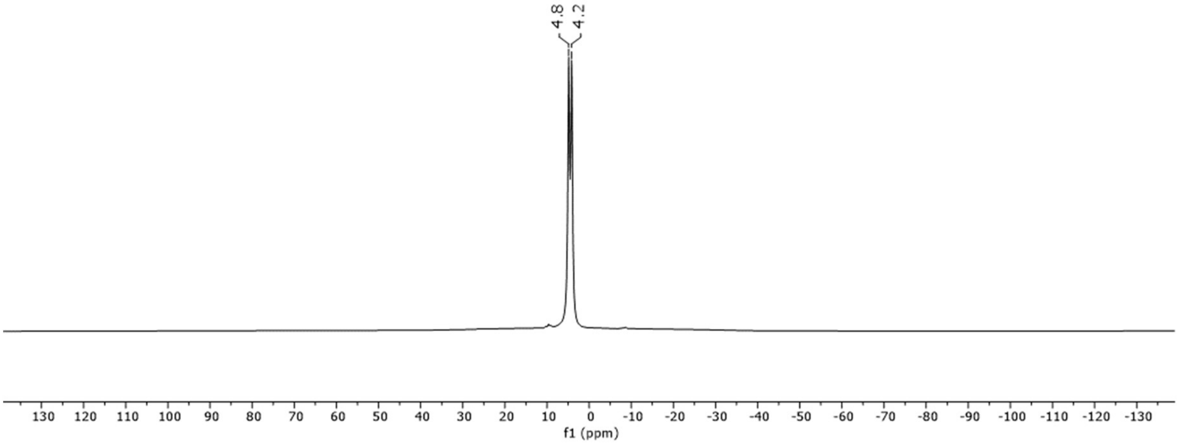

Figure S52: $^{11}B\{^{1}H\}$ NMR spectrum (161 MHz) of Cs·[2.2.2]-cryptand-[$FBEt_3$] in MeCN.

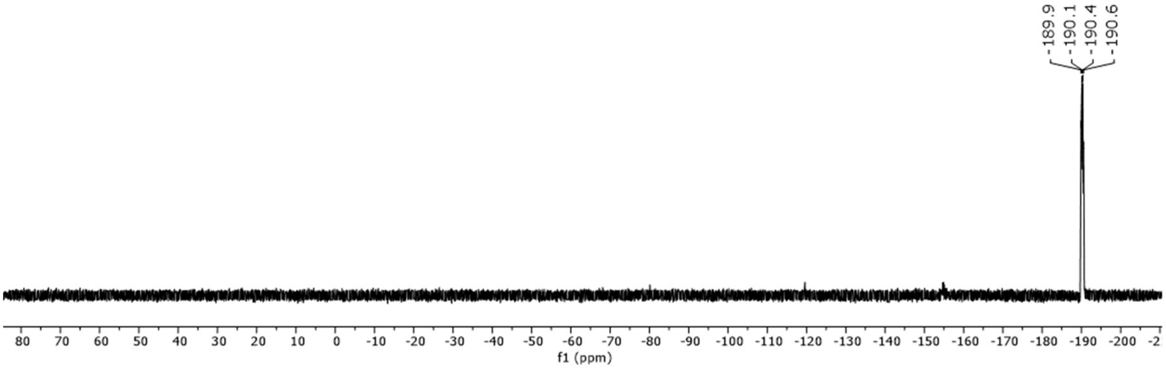

Figure S53: $^{19}F\{^{1}H\}$ NMR spectrum (470 MHz) of Cs·[2.2.2]-cryptand-[$FBEt_3$] in MeCN.

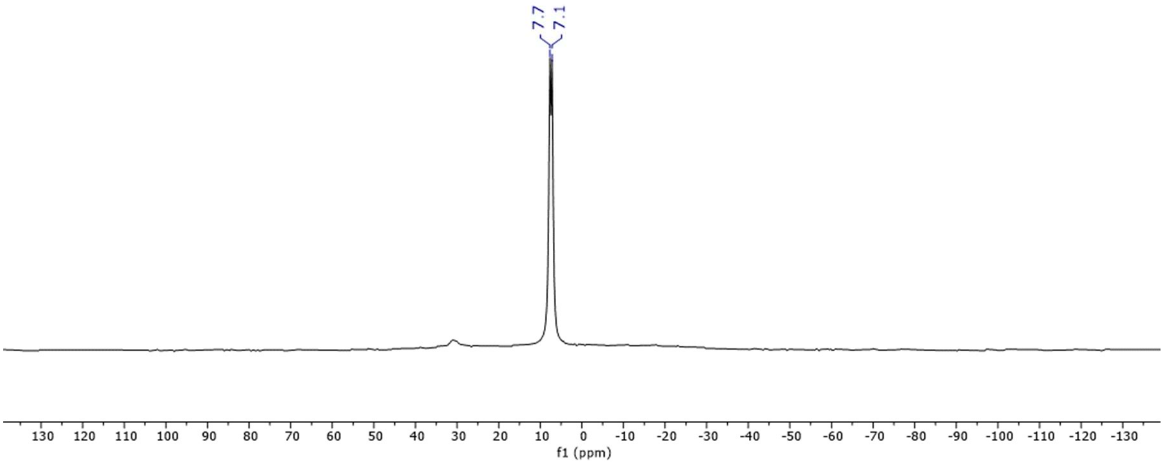

Figure S54: $^{11}B\{^1H\}$ NMR spectrum (161 MHz) of Cs[**1**–F] in MeCN.

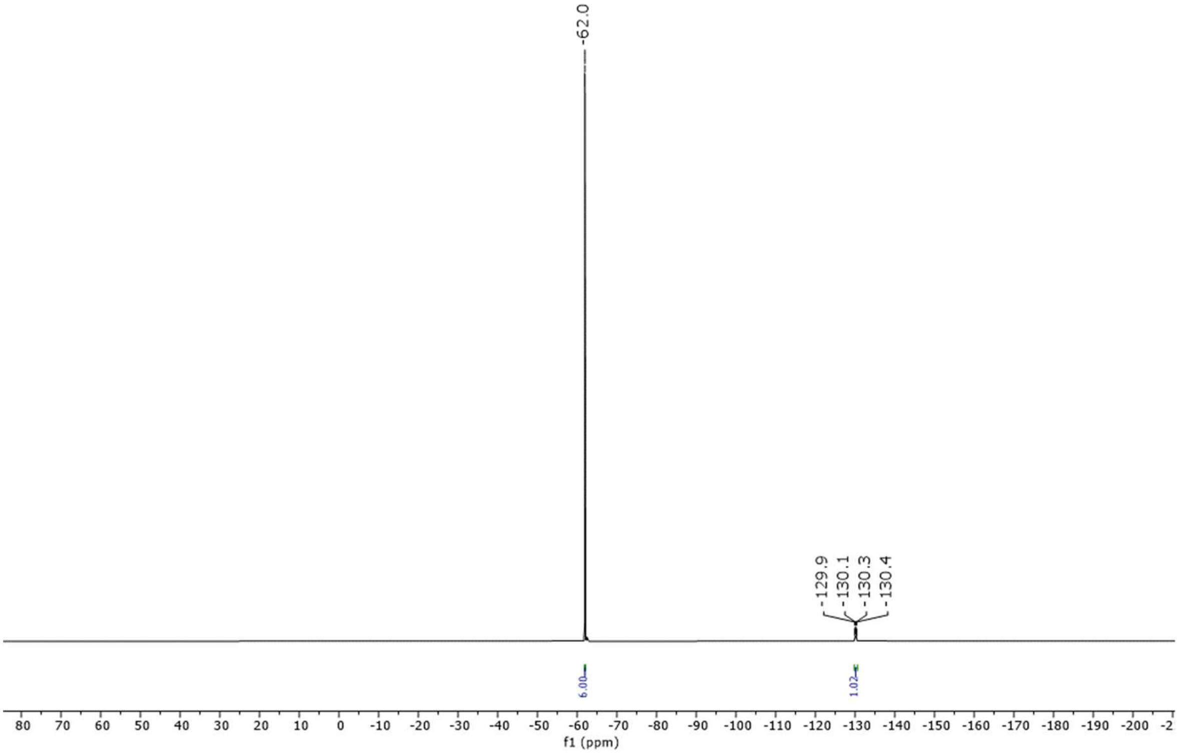

Figure S55: $^{19}F\{^1H\}$ NMR spectrum (470 MHz) of Cs[**1**–F] in MeCN.

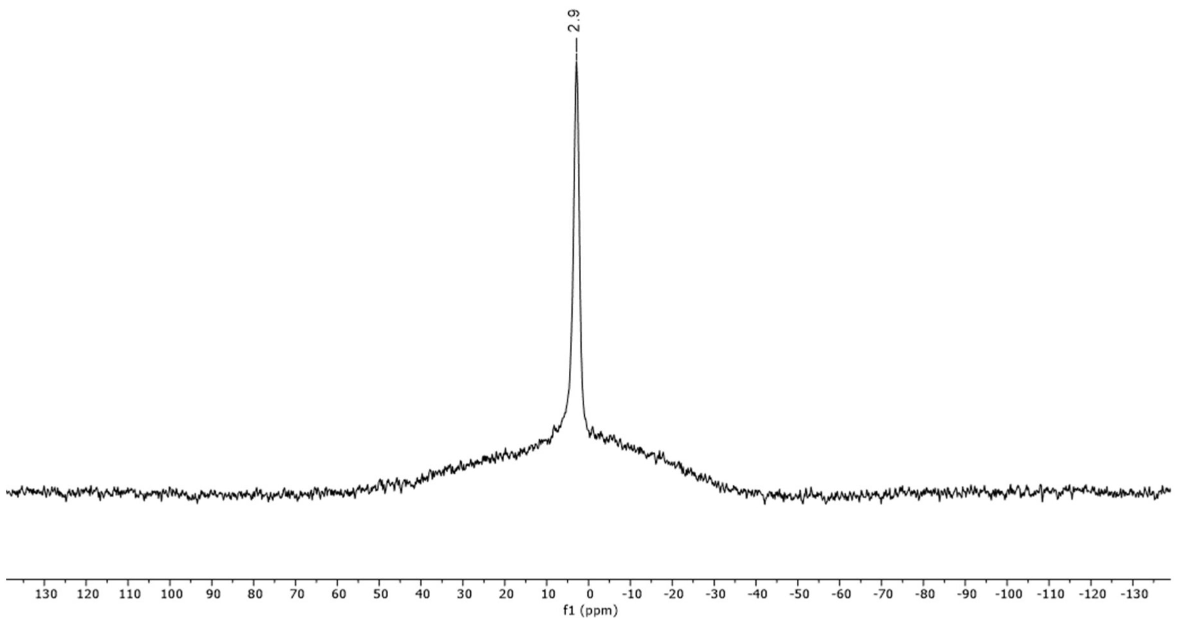

Figure S56: $^{11}B\{^{1}H\}$ NMR spectrum (161 MHz) of Cs·[2.2.2]-cryptand-[**1**–F] in $CDCl_3$.

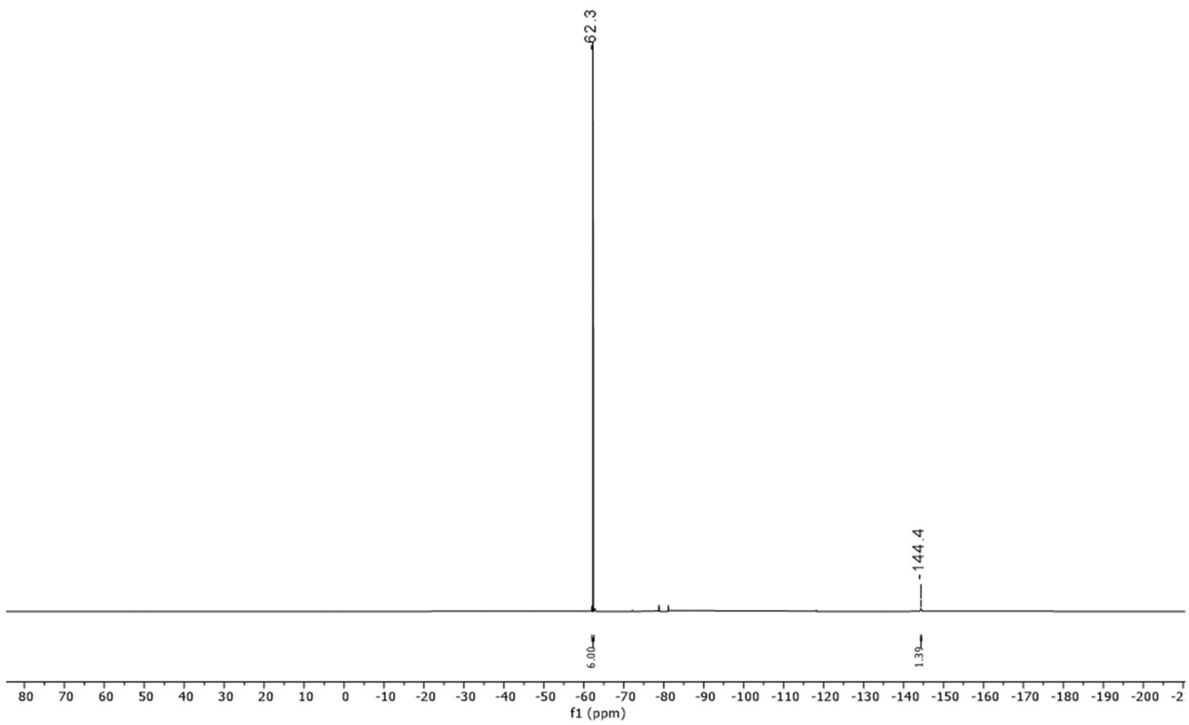

Figure S57: $^{19}F\{^{1}H\}$ NMR spectrum (470 MHz) of Cs·[2.2.2]-cryptand-[**1**–F] in $CDCl_3$.

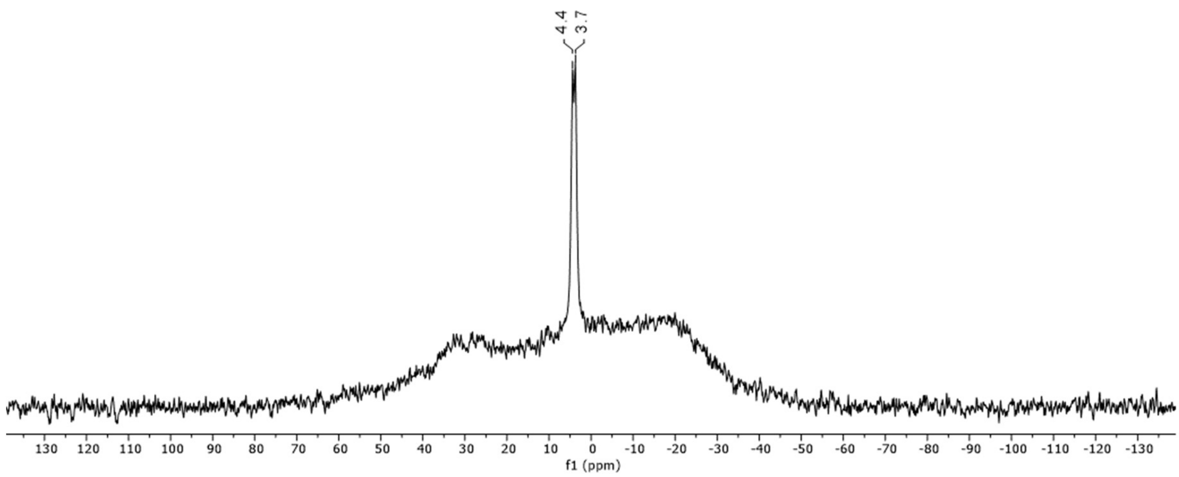

Figure S58: $^{11}B\{^{1}H\}$ NMR spectrum (161 MHz) of Cs[**5**–F] in MeCN.

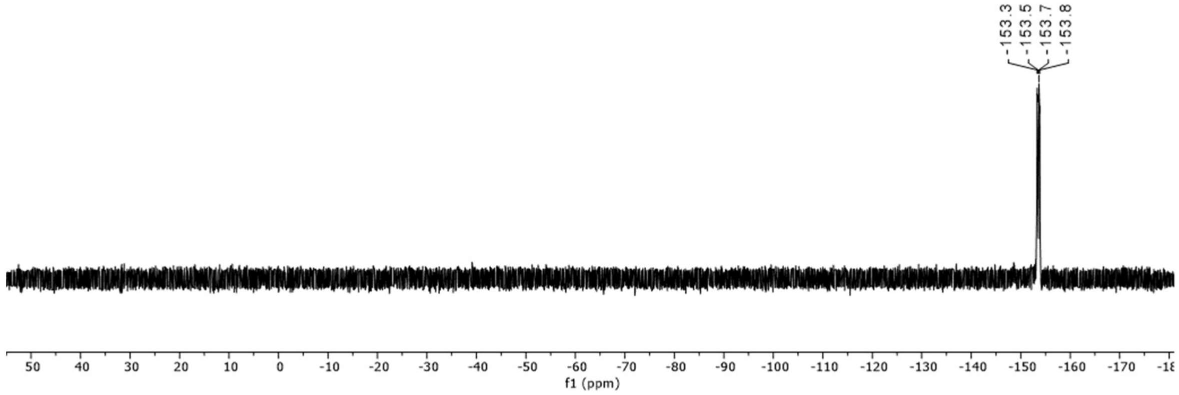

Figure S59: $^{19}F\{^{1}H\}$ NMR spectrum (470 MHz) of Cs[**5**–F] in MeCN.

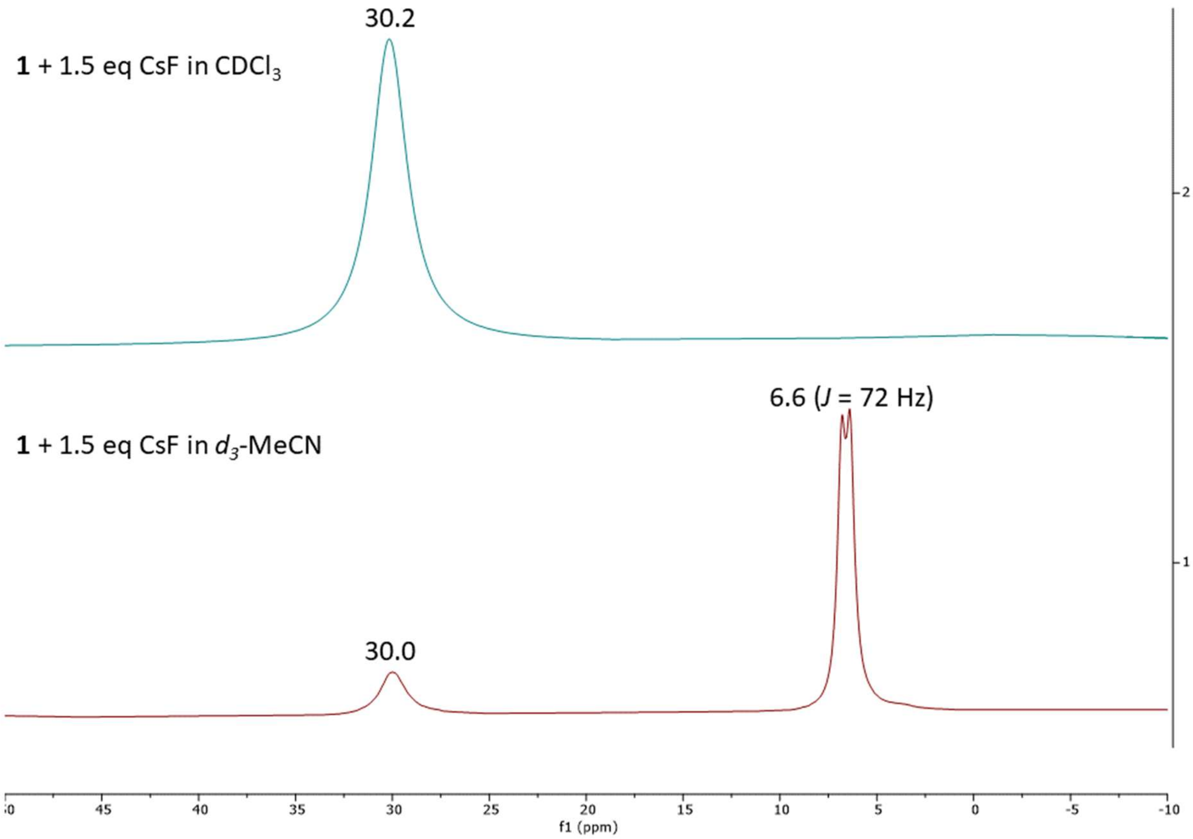

Figure S60: $^{11}B\{^{1}H\}$ NMR spectra of **1** in the presence of 1.5 eq CsF in either $CDCl_3$ (top) or d_3-MeCN (bottom).

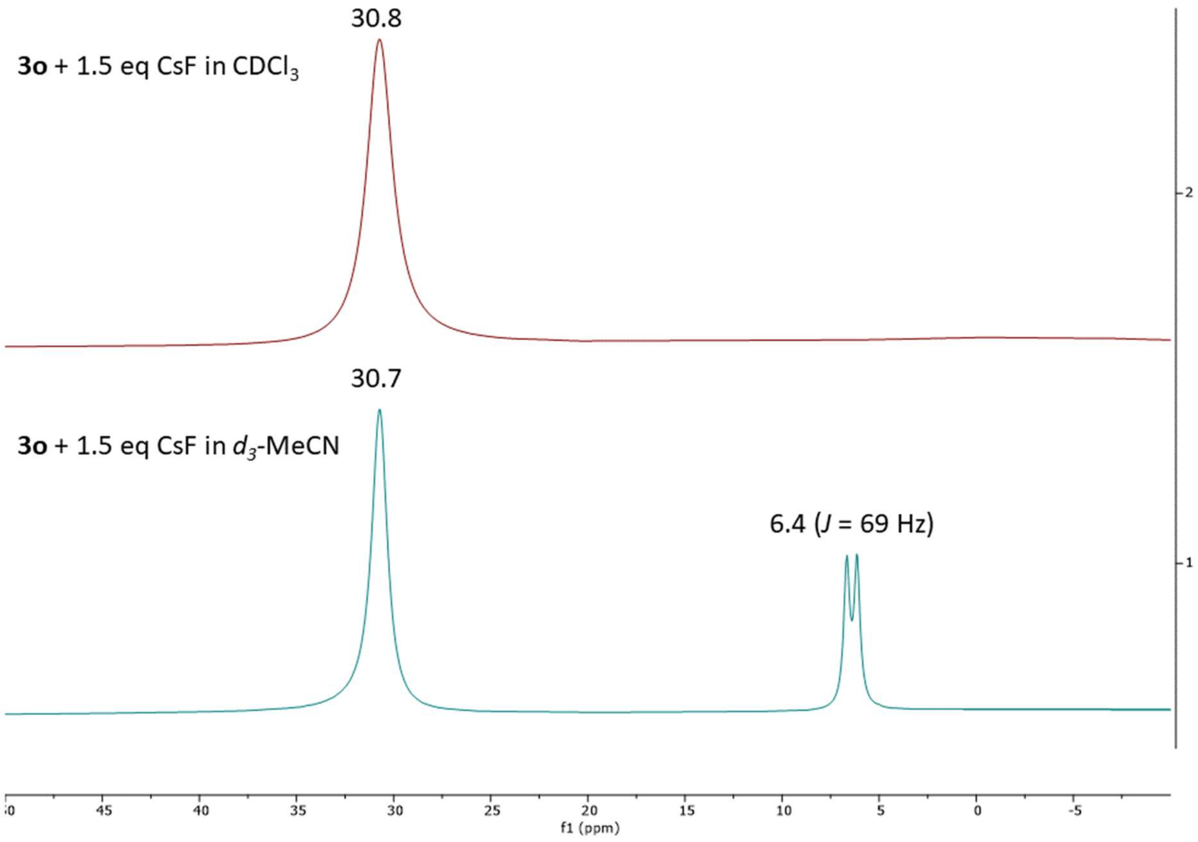

Figure S61: $^{11}B\{^1H\}$ NMR spectra of **3o** in the presence of 1.5 eq CsF in either $CDCl_3$ (top) or *d*$_3$-MeCN (bottom).

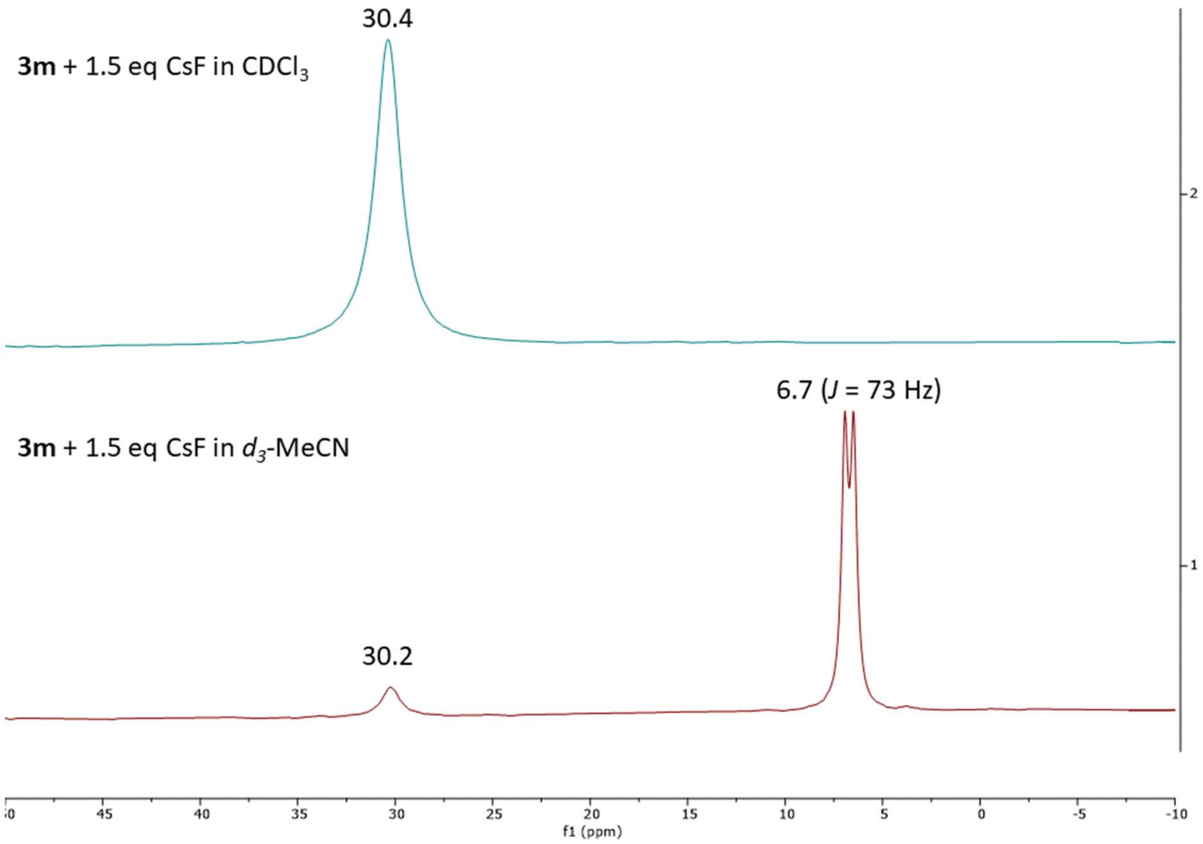

Figure S62: $^{11}B\{^{1}H\}$ NMR spectra of **3m** in the presence of 1.5 eq CsF in either $CDCl_3$ (top) or *d*$_3$-MeCN (bottom).

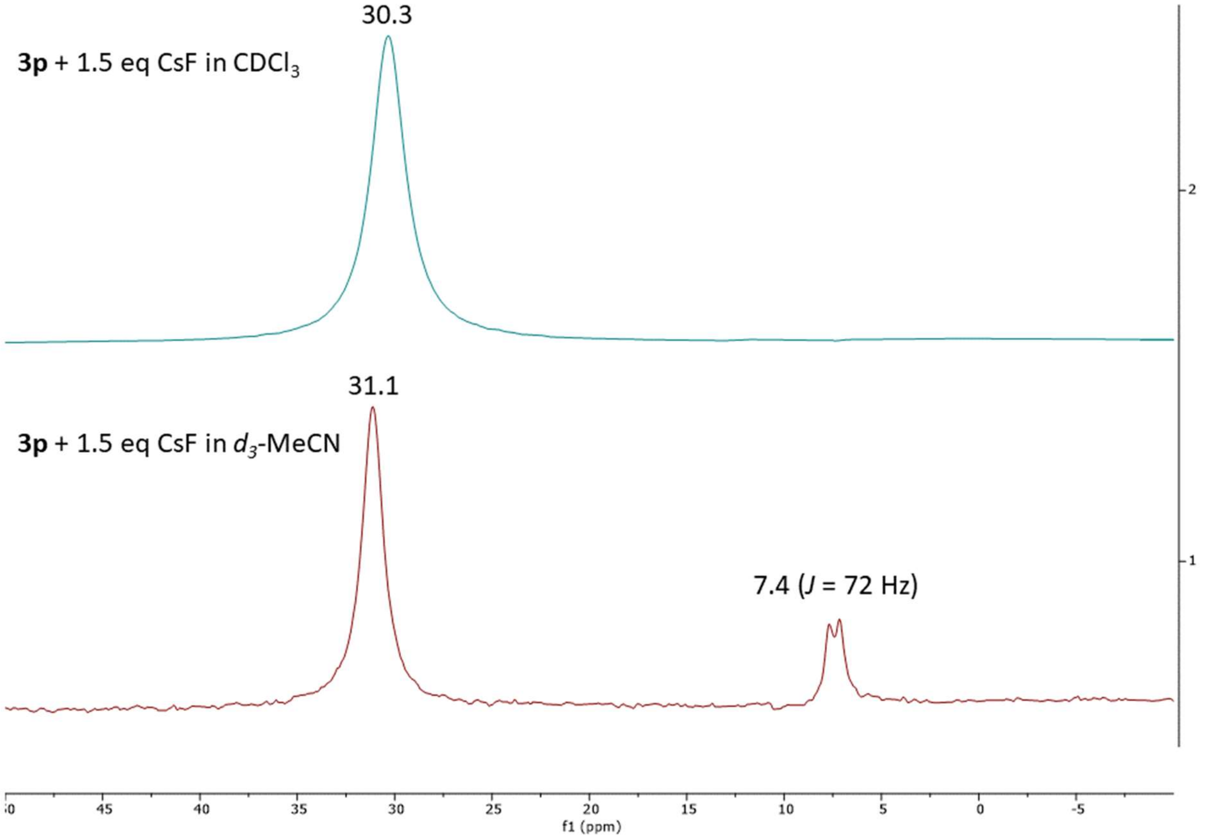

Figure S63: $^{11}B\{^{1}H\}$ NMR spectra of **3p** in the presence of 1.5 eq CsF in either $CDCl_3$ (top) or d_3-MeCN (bottom).

Plots of HPLC traces

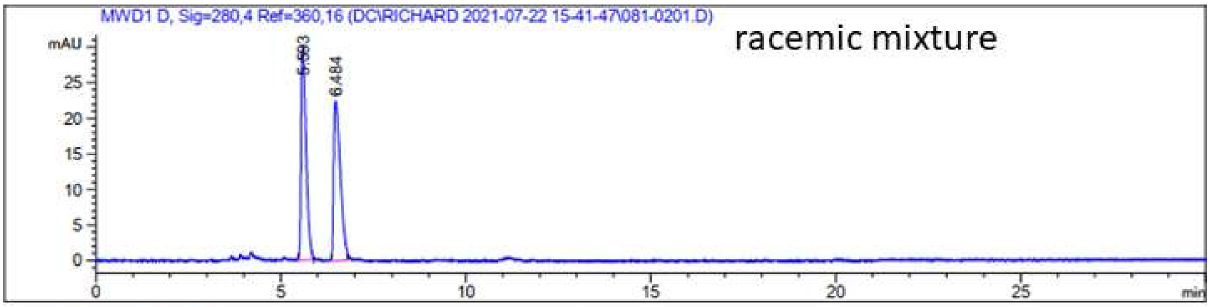

Peak #	RetTime [min]	Type	Width [min]	Area [mAU*s]	Height [mAU]	Area %
1	5.593	VV	0.1302	284.20605	30.22279	50.1279
2	6.484	VV	0.1506	282.75613	22.43319	49.8721

Totals : 566.96219 52.65599

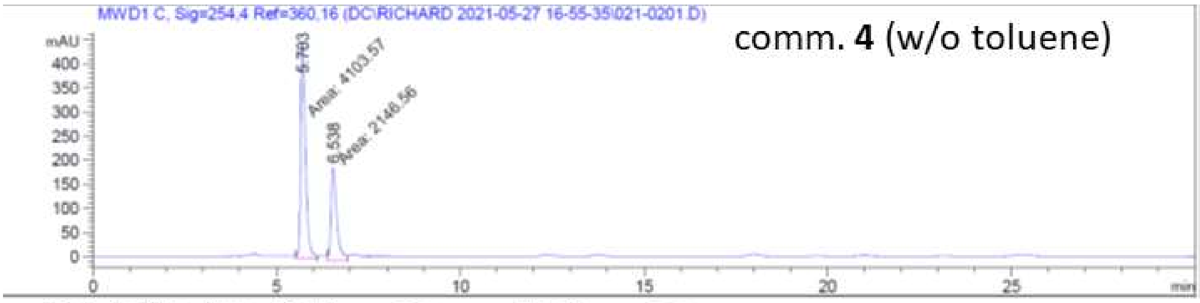

Peak #	RetTime [min]	Type	Width [min]	Area [mAU*s]	Height [mAU]	Area %
1	5.703	MM	0.1526	4103.56738	448.10626	65.6558
2	6.538	MM	0.1858	2146.55664	192.50311	34.3442

Totals : 6250.12402 640.60938

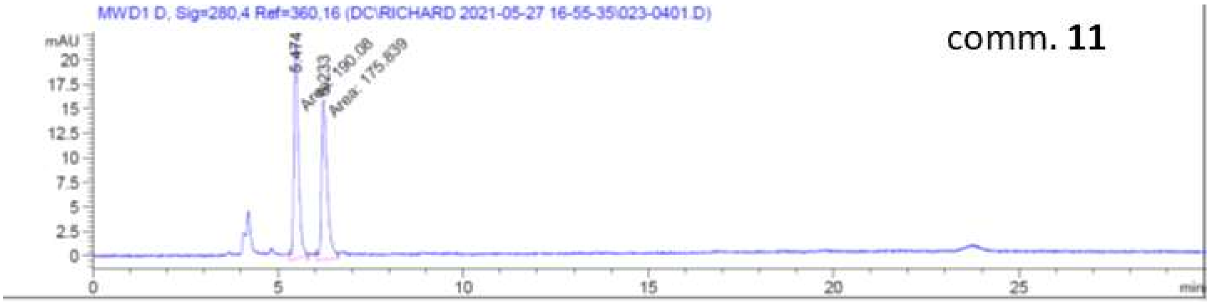

Peak #	RetTime [min]	Type	Width [min]	Area [mAU*s]	Height [mAU]	Area %
1	5.474	MM	0.1437	190.08029	22.04663	51.9460
2	6.233	MM	0.1799	175.83858	16.28855	48.0540

Totals : 365.91887 38.33518

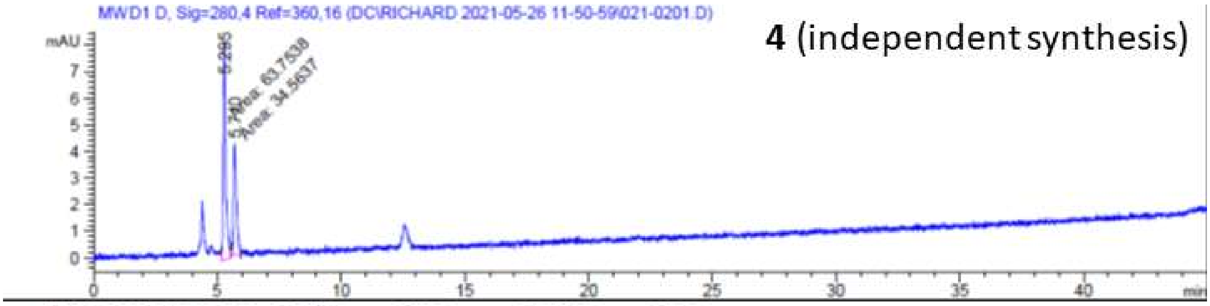

Peak #	RetTime [min]	Type	Width [min]	Area [mAU*s]	Height [mAU]	Area %
1	5.295	MM	0.1302	63.75382	8.15832	64.8448
2	5.710	MM	0.1370	34.56368	4.20481	35.1552

Totals : 98.31750 12.36313

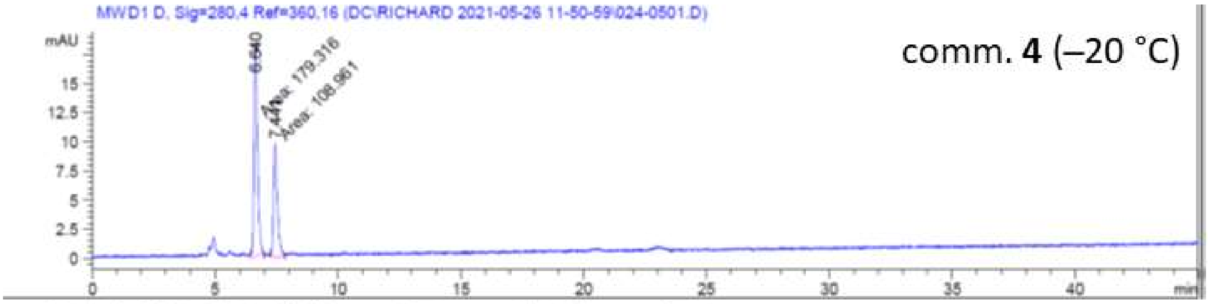

Peak #	RetTime [min]	Type	Width [min]	Area [mAU*s]	Height [mAU]	Area %
1	6.640	MM	0.1623	179.31590	18.41722	62.2027
2	7.441	MM	0.1869	108.96104	9.71661	37.7973

Totals : 288.27694 28.13383

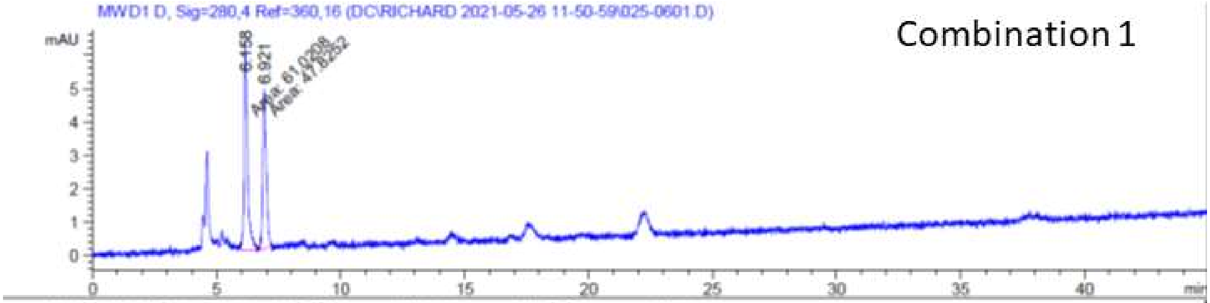

Peak #	RetTime [min]	Type	Width [min]	Area [mAU*s]	Height [mAU]	Area %
1	6.158	MM	0.1625	61.02075	6.25854	56.0616
2	6.921	MM	0.1661	47.82518	4.79758	43.9384

Totals : 108.84593 11.05612

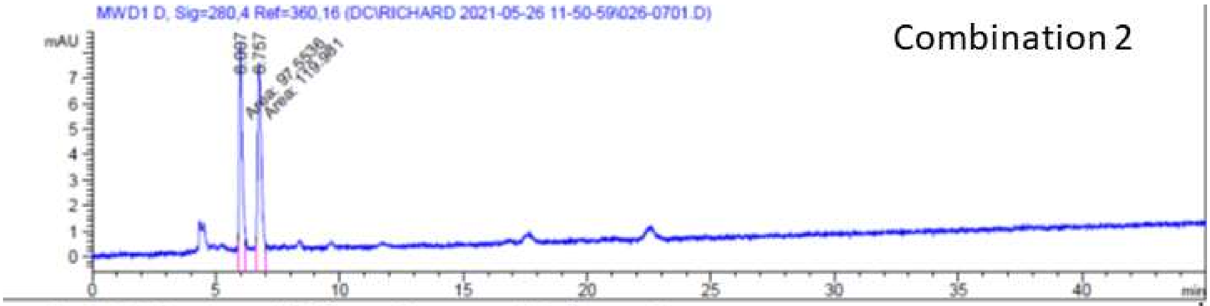

Peak #	RetTime [min]	Type	Width [min]	Area [mAU*s]	Height [mAU]	Area %
1	6.007	MM	0.1699	97.55363	9.57057	44.8451
2	6.757	MM	0.2236	119.98078	8.94354	55.1549
Totals :				217.53441	18.51411	

References

S1 F. J. Weigert, *J. Org. Chem.*, 1980, **45**, 3476–3483.
S2 G. A. Olah, X.-Y. Li, Q. Wang and G. K. Surya Prakash, *Synthesis*, 1993, **1993**, 693–699.
S3 A. Zweig, R. G. Fischer and J. E. Lancaster, *J. Org. Chem.*, 1980, **45**, 3597–3603.
S4 J. E. Power, M. Foroozandeh, R. W. Adams, M. Nilsson, S. R. Coombes, A. R. Phillips and G. A. Morris, *Chem. Commun.*, 2016, **52**, 2916–2919.
S5 G. Pupo, A. C. Vicini, D. M. H. Ascough, F. Ibba, K. E. Christensen, A. L. Thompson, J. M. Brown, R. S. Paton and V. Gouverneur, *J. Am. Chem. Soc.*, 2019, **141**, 2878–2883.
S6 G. Pupo, F. Ibba, D. M. H. Ascough, A. C. Vicini, P. Ricci, K. E. Christensen, L. Pfeifer, J. R. Morphy, J. M. Brown, R. S. Paton and V. Gouverneur, *Science*, 2018, **360**, 638–642.
S7 F. Beaulieu, L. P. Beauregard, G. Courchesne, M. Couturier, F. Laflamme and A. L'Heureux, *Org. Lett.*, 2009, **11**, 5050–5053.
S8 H. C. Brown and G. G. Pai, *J. Org. Chem.*, 1985, **50**, 1384–1394.
S9 B. Hatano, K. Nagahashi and T. Kijima, *J. Org. Chem.*, 2008, **73**, 9188–9191.
S10 S. K. Kristensen, S. L. R. Laursen, E. Taarning and T. Skrydstrup, *Angew. Chemie Int. Ed.*, 2018, **57**, 13887–13891.
S11 J. V. Bhaskar Kanth and M. Periasamy, *Tetrahedron*, 1993, **49**, 5127–5132.
S12 D. J. Mathre, T. K. Jones, L. C. Xavier, T. J. Blacklock, R. A. Reamer, J. J. Mohan, E. T. T. Jones, K. Hoogsteen, M. W. Baum and E. J. J. Grabowski, *J. Org. Chem.*, 1991, **56**, 751–762.
S13 Bruker, Bruker AXS, Inc. SAINT, Version 7.68A; Bruker AXS: Madison WI 2009
S14 G. M. Sheldrick, SADABS, Version 2008/2; University of Göttingen: Germany, 2003.
S15 O. V. Dolomanov, L. J. Bourhis, R. J. Gildea, J. A. K. Howard and H. Puschmann, *J. Appl. Crystallogr.*, 2009, **42**, 339–341.
S16 L. Palatinus and G. Chapuis, *J. Appl. Crystallogr.*, 2007, **40**, 786–790.
S17 L. Palatinus and A. Van Der Lee, *J. Appl. Crystallogr.*, 2008, **41**, 975–984.
S18 L. Palatinus, S. J. Prathapa and S. Van Smaalen, *J. Appl. Crystallogr.*, 2012, **45**, 575–580.
S19 G. M. Sheldrick, *Acta Chryst. Sect. A*, 2008, **64A**, 112–122.
S20 Gaussian 09, Revision E.01, M. J. Frisch, G. W. Trucks, H. B. Schlegel, G. E. Scuseria, M. A. Robb, J. R. Cheeseman, G. Scalmani, V. Barone, B. Mennucci, G. A. Petersson, H. Nakatsuji, M. Caricato, X. Li, H. P. Hratchian, A. F. Izmaylov, J. Bloino, G. Zheng, J. L. Sonnenberg, M. Hada, M. Ehara, K. Toyota, R. Fukuda, J. Hasegawa, M. Ishida, T. Nakajima, Y. Honda, O. Kitao, H. Nakai, T. Vreven, J. A. Montgomery, Jr., J. E. Peralta, F. Ogliaro, M. Bearpark, J. J. Heyd, E. Brothers, K. N. Kudin, V. N. Staroverov, T. Keith, R. Kobayashi, J. Normand, K. Raghavachari, A. Rendell, J. C. Burant, S. S. Iyengar, J. Tomasi, M. Cossi, N. Rega, J. M. Millam, M. Klene, J. E. Knox, J. B. Cross, V. Bakken, C. Adamo, J. Jaramillo, R. Gomperts, R. E. Stratmann, O. Yazyev, A. J. Austin, R. Cammi, C. Pomelli, J. W. Ochterski, R. L. Martin, K. Morokuma, V. G. Zakrzewski, G. A. Voth, P. Salvador, J. J. Dannenberg, S. Dapprich, A. D. Daniels, O. Farkas, J. B. Foresman, J. V. Ortiz, J. Cioslowski, and D. J. Fox, Gaussian, Inc., Wallingford CT, 2013.
S21 A. D. Becke, *J. Chem. Phys.*, 1993, **98**, 1372–1377.
S22 S. Grimme, S. Ehrlich and L. Goerigk, *J. Comput. Chem.*, 2011, **32**, 1456–1465.
S23 A. Schäfer, C. Huber and R. Ahlrichs, *J. Chem. Phys.*, 1994, **100**, 5829–5835.
S24 S. Kozuch, D. Gruzman and J. M. L. Martin, *J. Phys. Chem. C*, 2010, **114**, 20801–20808.
S25 A. V. Marenich, C. J. Cramer and D. G. Truhlar, *J. Phys. Chem. B*, 2009, **113**, 6378–6396.
S26 Y. Zhao, N. E. Schultz and D. G. Truhlar, *J. Chem. Theory Comput.*, 2006, **2**, 364–382.

S27 P. Erdmann, J. Leitner, J. Schwarz and L. Greb, *ChemPhysChem*, 2020, **21**, 987–994.
